# Ultra-deep sequencing of 45S rDNA to discern intragenomic diversity in three *Chrysodeixis* species for molecular identification

**DOI:** 10.1038/s41598-023-39673-7

**Published:** 2023-08-10

**Authors:** Frida A. Zink, Luke R. Tembrock, Alicia E. Timm, Todd M. Gilligan

**Affiliations:** 1 Department of Agricultural Biology, Colorado State University, Fort Collins, CO USA; 2 Pest Identification Technology Laboratory, USDA-APHIS-PPQ-Science and Technology, Fort Collins, CO USA

**Keywords:** Agricultural genetics, Molecular evolution, Entomology

## Abstract

Species identification is necessary to prevent introductions of exotic plant pests through global trade. Many of these pests are understudied and lack publicly available DNA sequence data on which rapid molecular identification methods can be based. One such lineage is the genus *Chrysodeixis,* which includes three species of potential concern for United States trade initiatives: *C. includens*, *C. chalcites*, and *C. eriosoma*. Here we describe a method to generate robust 45S rDNA profiles using long read sequencing in order to clarify evolutionary relationships and develop a real-time PCR identification technique. Such an identification tool will be useful in rapidly differentiating between *Chrysodeixis* species of quarantine concern where traditional morphological identification methods are insufficient. Molecular methods such as this greatly reduce the time spent identifying each specimen, allow for detection of eDNA, vastly increase throughput, and increase the probability of detection. The methods presented here will be generally adaptable to any understudied lepidopteran taxa that necessitates a molecular diagnostic assay and, with adjustment or testing of the primers, could be applied to any group for which development of rDNA profiles in a benchtop setting would prove useful.

## Introduction

Species diagnostics are increasingly important in a globalized world where human-mediated translocation of species (especially plants and insects) is occurring at a rate higher than ever before^1,2^. With increased movement of people and global shipping of agricultural commodities including food crops, cut flowers, and wood packing material, the ability to rapidly detect and identify any associated insects is paramount to limiting the introduction and establishment of invasive pest species^3,4^. In the United States, inspections of cargo and commodities at ports of entry and domestic surveys for exotic pests are used to screen for the presence of insect pests. The identification of these specimens can be difficult and often relies on an accurate record of the host on which the organism was found and origin of the shipment, both of which can be convoluted due to transshipment through multiple locations^5^. In many cases, intercepted pests can only be identified to the family, subfamily, or genus. This is especially true for Lepidoptera, which are almost exclusively intercepted in the larval stage.

Identification of insects captured during domestic surveys is also challenging because cross-attraction to pheromone lures can result in dozens or hundreds of similar non-targets being captured in a single trap. Rapid identification of potential targets is desirable to detect invasive species prior to establishment (i.e., the lag phase^6^), but doing so often requires dissecting each specimen or a molecular approach, such as cytochrome c oxidase 1 (CO1) DNA barcoding^7^. Both approaches are time consuming and may not be reliable for species that are previously undescribed, poorly characterized, or that lack publicly available sequence data. To improve the chances of invasive species detection, rapid or high-throughput molecular assays have been developed for some commonly intercepted and potentially invasive insect pest species (e.g.^8–11^). Not only do high-throughput molecular identification techniques increase the probability of detection, they also dramatically reduce the time and cost needed to complete identifications, especially in the case of multiplexed assays^12,13^. Some species that pose a high potential for invasion are also understudied and lack basic information such as phylogenetic placement and description of sister species, phylogeographic variation within the species, or publicly available sequence data^7,14^.

While a large database of CO1 sequences is available for many species (i.e., BOLD^15^, GenBank^16^), this region can be poorly suited for development of DNA sequence-based techniques like real-time PCR due to high AT content, low interspecies variation, and/or high intraspecies variation resulting from rapid evolution and genetic drift^17^. In these situations, species identification can often be reliably carried out using regions of 45S ribosomal RNA (rRNA). Among the first nucleic acids to be sequenced, the 45S rRNA is highly abundant in the genome and relatively short in length^18^. The rRNA unit is comprised of structurally conserved tandem repeats made up of an external transcribed spacer (ETS), 18S rRNA, internal transcribed spacer (ITS) 1, 5.8S rRNA, ITS2, and 28S rRNA^19^. These properties of rRNA, as well as sequence conservation in the coding regions and high rates of evolution in the non-coding ITS regions in the underlying ribosomal DNA (rDNA), led to the use of this locus for phylogenetic studies across the entire tree of life^20,21^. From these phylogenetic studies, large amounts of rDNA sequence data were made publicly available from which researchers have developed methods for rapid species identification. These loci are ideal for such molecular assays because the ITS regions are relatively conserved within species, highly variable between species (with both point mutations and indels), and present in high copy numbers in the genome. This makes them ideal candidates for developing probe-based PCR assays^8,22,23^ and allows for amplification of these sequences even from degraded DNA or in high-complexity environmental samples^24,25^. Because 18S, 28S, and 5.8S rRNA-coding sequences are highly conserved, the development of universal primers to amplify this entire region is possible even if no rDNA sequence data is available for the species in question. However, due to the high number of copies within a single genome, nucleotide differences exist between copies within an individual. These intraindividual variations are considered ribotypes^26,27^, and such ribotypic variation should be avoided when developing a reliable DNA-based technique for species identification, yet characterization of ribotypic variation is rarely completed. The use of a high-throughput sequencing method is, therefore, useful for characterizing ribotypic diversity, and for differentiation between fixed and transient variations that exist between repeats.

To explore the utility and viability of generating an rDNA profile to develop molecular identification techniques, we considered an economically important group for which little molecular research is available: *Chrysodeixis* (Hübner), a genus of moths in the subfamily Plusiinae (Noctuidae). Within the genus, three species are of economic concern in the U.S. and would benefit from molecular identification. The first is *Chrysodeixis includens* (Walker) which occurs throughout much of the Americas, where it prefers sub-tropical to tropical climates. Although the species likely overwinters only in the southern or southeastern portions of the U.S., migrating as far as Nova Scotia, it is the most common plusiine in agricultural areas in the eastern U.S.^28^. Larvae are polyphagous, having been recorded on 174 plant species in 39 families, and *C. includens* is one of the most important soybean pests in its native range (e.g.^29–31^). The second, *C. eriosoma* (Doubleday), is found in tropical habitats in Asia and Oceania, including populations in the Hawaiian Islands, French Polynesia^32^, and New Zealand where it is considered one of the most important pests of horticultural crops^33^. Larvae are polyphagous and feed on a number of species from families including Asteraceae, Fabaceae, and Solanaceae. Lastly, *C. chalcites* (Esper), is found in subtropical to tropical habitats throughout the Mediterranean, in western Asia, and throughout much of Africa^34^. Like the two previously discussed species, larvae are highly polyphagous, and will feed on plants in Asteraceae, Brassicaceae, Fabaceae, Poaceae, Solanaceae, and many others^35^. Larvae of *C. chalcites* are considered major pests on tomato, alfalfa, potato, cucumber, peppers, and other economically important crops^36^. While none of the three species overwinter in temperate regions, *C. chalcites* has been known to establish year-round populations in greenhouses in Europe, as far north as Norway^34^. It has likewise been discovered in Michigan, Ohio, British Columbia, and Ontario^36–38^. Specimens of *C. chalcites* have been intercepted at federal ports of entry in the United States over 300 times between 1984 and 2014^34^, including 84 times in California alone, and is predicted to have a severe impact if established there^39^.

Because of their morphological and biological similarity, the taxonomy and identification of *C. chalcites* and *C. eriosoma* is complicated. The type specimens of *C. chalcites* and *C. eriosoma* were collected from geographically disparate locations, but they were generally treated as synonyms until Kostrowicki^40^ separated the two species based on minor differences in wing pattern and male genitalia. Later authors (e.g.^41^) accepted or rejected Kostrowiki’s delineation but generally determined more work was needed and that, if they were two species, they could not be reliably separated morphologically^36^. Both species’ ranges overlap in the Middle East and western Asia, and both are routinely found in India and Pakistan during surveys of plusiine diversity^42,43^. Specimens are regularly misidentified and the current method for separation is by CO1 barcoding and consideration of geographic origin^44^. *Chrysodeixis* specimens reported from British Columbia in 2005 were originally described as *C. eriosoma*^38^ but were later determined to be *C. chalcites* by Lafontaine and Schmidt^44^.

Due to the high number of interceptions of plusiine larvae on imported commodities and the recent discoveries of invasive populations of *C. chalcites* in North America, a molecular method to diagnose individuals rapidly and reliably is necessary. Here, we present a real-time PCR method for the identification of economically important *Chrysodeixis* that are routinely encountered during domestic surveys or intercepted at ports of entry, using deep coverage long-read sequencing. Portions of the 45S rDNA profiles for each specimen were used, along with CO1 sequence data, to clarify the relationships between the three species. The 45S rDNA sequencing method described here is universally applicable to all Lepidoptera and adaptable to nearly any lineage with suitable adjustment of the primers.

## Results

### rDNA profile development and analysis

**Figure 1:**
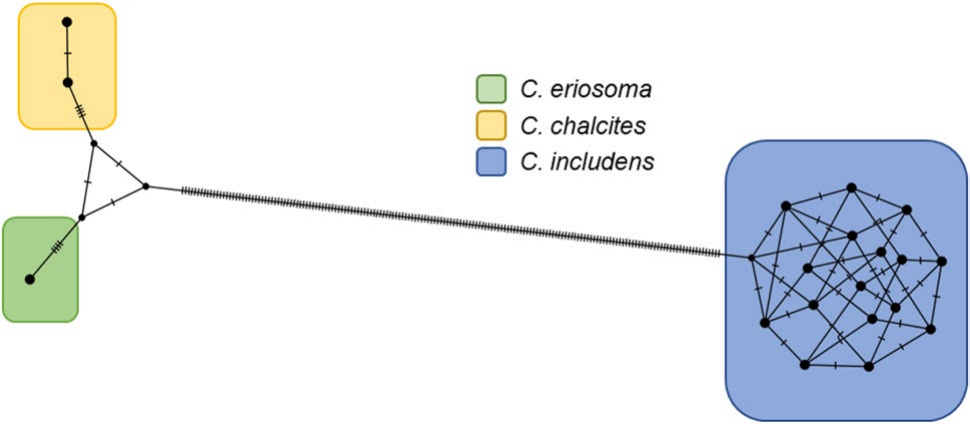
TCS network of detectable ribotypic diversity in *C. eriosoma*, *C. chalcites*, and *C. includens* 45S rDNA final assemblies. Dots represent ribotypes within individuals: 1 ribotype within *C. eriosoma*, two within *C. chalcites*, and 16 possible combinations of four ribotypic loci in *C. includens*. Hatch marks between nodes represent SNVs separating species and ribotypes.

Long-read sequencing (Oxford Nanopore Technologies) was used to develop a 45S rDNA profile from a single individual for each of three *Chrysodeixis* species using amplicons from long-range PCRs. The assembled amplicons were 6474 bp for *C. includens*, 6493 bp for *C. eriosoma*, and 6494 bp for *C. chalcites*. Individual rDNA sequences were variable with both indels and point mutations throughout the region. Most ambiguities were low frequency, possibly due to PCR or sequencing error, and did not affect the final assembly. Within the rDNA profiles for each species sample, fixed ambiguities identified using LoFreq^45^ were considered “ribotypes” (Supplementary Table 1). For both *C. includens* and *C. chalcites*, four loci were selected for investigation using Sanger sequencing. Of these, one ribotype that appears as a fixed W (A or T) was detected in *C. chalcites* at position 4775 using LoFreq and confirmed using Sanger sequencing (Supplementary Fig. 1A), the remaining three were inconclusive or undetectable by Sanger sequencing. Four (at 1916, 2426, 2535, and 4502) of seven ribotypes found in *C. includens* using LoFreq were confirmed by Sanger sequencing (Supplementary Fig. 1B–E). The final assembly of the *C. eriosoma* rDNA segment revealed no fixed variations. A TCS network of ribotypic diversity within and among the three individuals sequenced is shown in Fig. 1. These intragenomic ribotypic variants were avoided during real-time PCR primer and probe design (Supplementary File 1).

### Species delimitation

**Figure 2:**
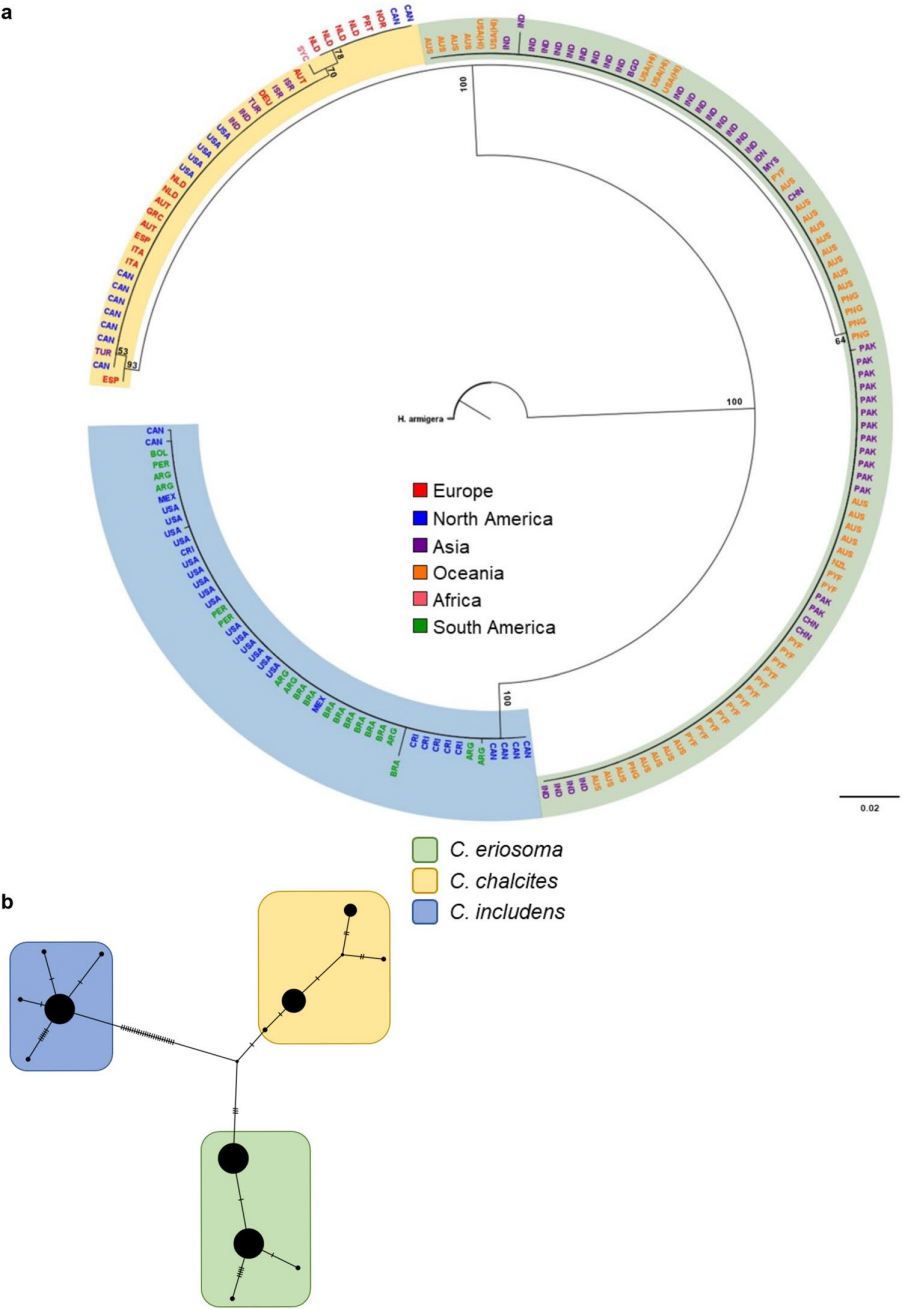
Analysis of *Chrysodeixis* species based on CO1 resolves evolutionary relationships between *C. includens*, *C. chalcites*, and *C. eriosoma*. (**A**) A neighbor-joining tree shows variation within and between species. Each specimen is labeled with a three-letter country code indicating its collection location, and color coded by the continent/region. Branch labels represent jackknife support. *Helicoverpa armigera* was set as an outgroup. Scale bar represents genetic distance. (**B**) A TCS network of the same sequences shows the number of parsimony informative SNVs separating each group. Hatch marks between nodes indicate the number of SNVs and haplotypes are represented by large circles.

In the alignment of the rDNA sequences from *C. chalcites* and *C. eriosoma*, 13 variable locations were found between the two, 11 of which were within the ITS regions but were not flanked by sequences suitable for primer or probe design, and two in 28S (Supplementary File 1). Because of the past debates over the species status of *C. eriosoma*, and these sequencing results, we used available population-wide CO1 barcode data for each species and our own specimen archive to examine the relationship. Using CO1 sequence data from *C. includens*, *C. chalcites*, and *C. eriosoma*, Neighbor-Joining (NJ) and Bayesian Inference (BI) trees both resolved three well supported clades corresponding to each of the three species (Fig. 2A; Supplementary Fig. 2A). The TCS network produced from the same alignment shows haplotypic diversity within each species (Fig. 2B). As shown on the Bayesian CO1 tree (Supplementary Fig. 2A) and the TCS network (Fig. 2B), specimens of *C. eriosoma* were sorted into two haplogroups, which correspond roughly to collection location with specimens from around the Middle East, India, Pakistan, and Southeast Asia grouping together and specimens from Australia, New Zealand, and French Polynesia grouping together (Fig. 2A; Supplementary Fig. 2A). The Hawaiian specimens resolved with those from India and both haplogroups were collected in Papua New Guinea. Conversely, *C. chalcites* showed increased intra-clade branching and longer branch lengths between groups, all with high branch support (Fig. 2A) and one large haplogroup with one smaller haplogroup and two singletons in the TCS network (Fig. 2B). Lastly, *C. includens* showed no location-specific structure as all specimens resolve in a single clade despite the geographically dispersed collections (Fig. 2A) and one large haplogroup that spans the geographic range (Fig. 2B). A total of 28 Single Nucleotide Variations (SNVs) separate *C. includens* from both *C. chalcites* and *C. eriosoma* while only 4 SNVs separate the most closely related *C. chalcites* and *C. eriosoma* haplotypes.

**Figure 3:**
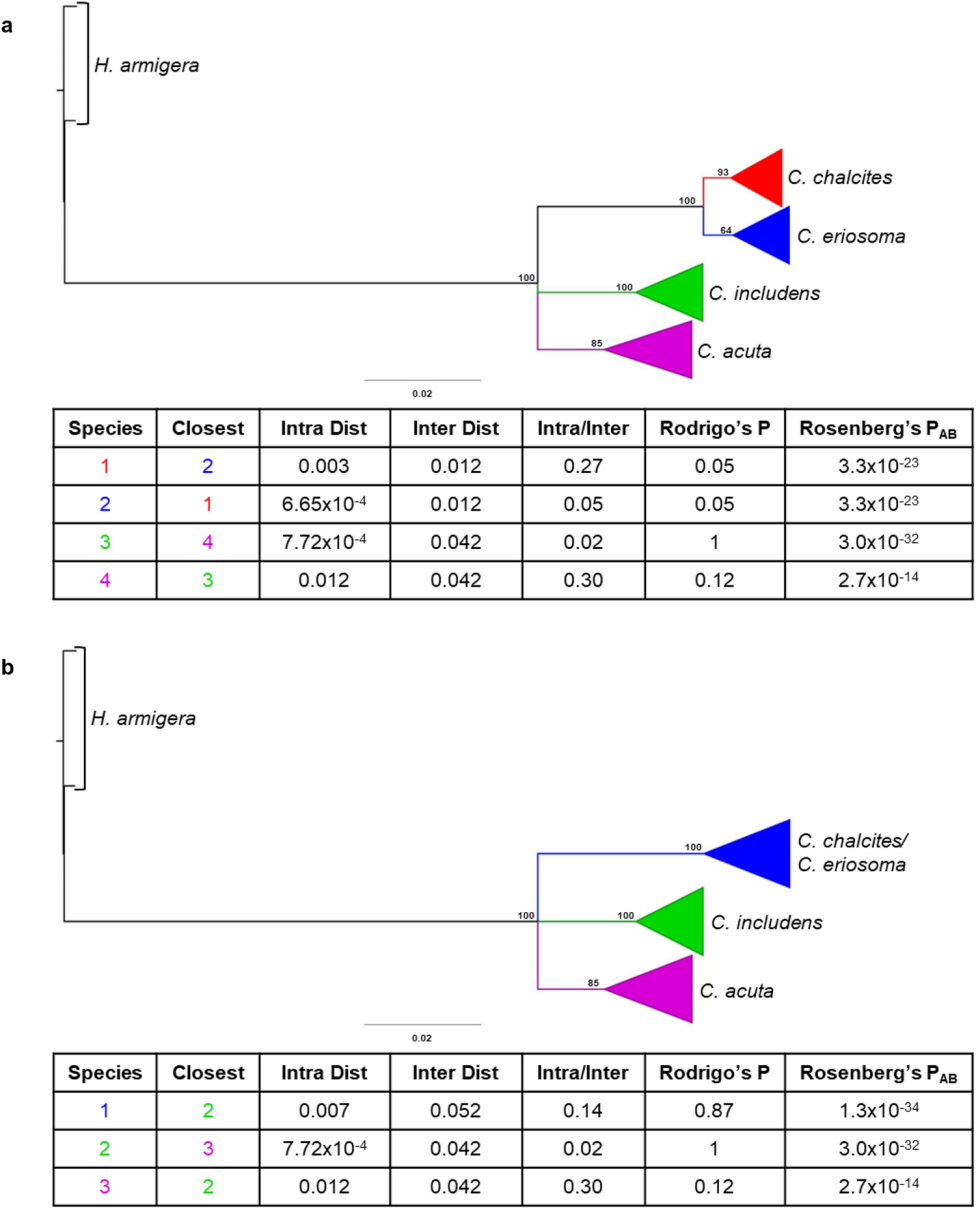
Species delimitation analysis was carried out using CO1 data following two hypotheses. (**A**) A neighbor-joining tree is shown comparing *C. chalcites*, *C. eriosoma*, *C. includens*, and *C. acuta* as 4 individual species. Each clade representing a species has been collapsed and color coded. (**B**) Shows the same tree with *C. chalcites* and *C. eriosoma* treated as a single species, collapsed together at the most recent common ancestor.

Species delimitation analysis was carried out to test the validity of the *C. chalcites*/*C. eriosoma* species split. Using Species Delimitation^46^, two hypotheses were tested on a CO1 NJ tree: *C. chalcites* and *C. eriosoma* as separate species (Fig. 3A) or *C. chalcites* and *C. eriosoma* as a single species (Fig. 3B). In both tests, the lineages are reciprocally monophyletic by Rosenberg’s P_AB_^47^, and under the single species hypothesis (Fig. 3B), the branching pattern is deemed randomly distinct by Rodrigo’s P^48^. However, under the two species hypothesis (Fig. 3A), each lineage has a statistically significant Rodrigo’s P indicating there is selection drive in each branch. Conversely, *C. includens* is randomly distinct by Rodrigo’s P but is reciprocally monophyletic with *C. acuta*. Because there are two well supported clades in the small sample of *C. acuta* included in this analysis, these hypotheses were also tested for *C. acuta* as a comparator (Supplementary Fig. 3). Similarly, Rodrigo’s P indicates branching is random within the clade as a whole, it is reciprocally monophyletic with *C. includens* according to Rosenberg’s P, but the clades are supported as distinct when they are tested separately (Supplementary Fig. 3). The intra-clade distance for *C. acuta* is higher than the intra-clade distance for *C. chalcites* and *C. eriosoma* when they are considered a single species (Fig. 3B, Supplementary Fig. 3).

**Figure 4:**
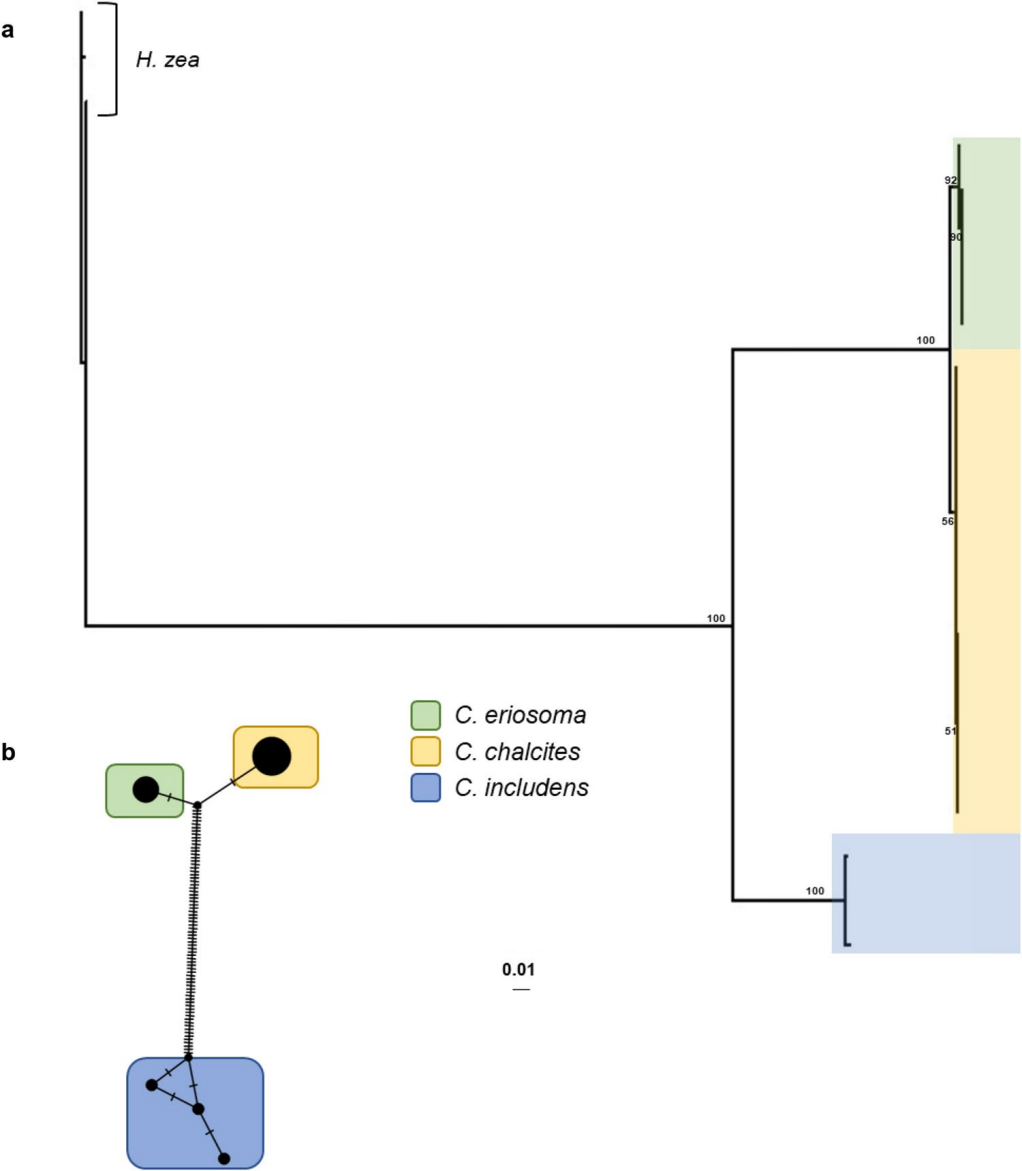
Analysis of *Chrysodeixis* species based on ITS1 sequencing. (**A**) A neighbor joining tree shows variation within and between clades containing each of the species analyzed: *C. includens*, *C. chalcites*, and *C. eriosoma*. Branch labels indicate jackknife support. *Helicoverpa zea* was set as the outgroup. Scale bar represents genetic distance. (**B**) A TCS network of the ITS1 sequences shows the parsimony informative SNVs (hatch marks) separating each group.

Using ITS1, an NJ tree (Fig. 4A) and a Bayesian analysis (Supplementary Fig. 2B) showed lower resolution between *C. chalcites* and *C. eriosoma* as compared to CO1 (Fig. 2A; Supplementary Fig. 2B). Furthermore, ITS1 from individuals of each species revealed less differentiation between them than the rDNA profiles contain (Supplementary File 1), which is supported by the TCS network, which shows more diversity within the small sample of *C. includens* than separates either of the larger samples of *C. eriosoma* and *C. chalcites* from each other (Fig. 4B). The small sample sizes and limited specimen collection locations for the ITS1 sequence data generated for this study hinder its application as a phylogeographic or species delimitation locus, so further analyses were not conducted.

### Real-time PCR assay development and testing

Development of a real-time PCR assay was carried out to run as a triplex probe-based assay with one oligo set to identify *C. includens* (FAM in ITS2), one oligo set to identify both *C. chalcites* and *C. eriosoma* (HEX in ITS1), and one oligo set to serve as a control (Quasar 670 in 18S^23^). All *Chrysodeixis* samples tested returned positive results for the expected probe as well as for the 18S control probe. There was no cross-reactivity between the FAM and HEX probes. The FAM probe had a Cq range of 11.57 to 25.44 (mean 15.77 ± SD 2.59) and the HEX probe had a Cq range of 15.19 to 23.98 (mean 18.77 ± 1.75 SD). The 18S control probe consistently returned a Cq value higher than those from either diagnostic probe for all three species and did not vary meaningfully in efficiency between species. The ΔCq between the FAM and Quasar 670 probes ranged from − 2.22 to 2.86 (mean 2.08 ± SD 0.70) and the ΔCq between the HEX and Quasar 670 probes ranged from 0.66 to 1.39 (mean 1.04 ± SD 0.19) indicating that the sensitivity for both diagnostic probes is higher than that of the control probe.

**Figure 5:**
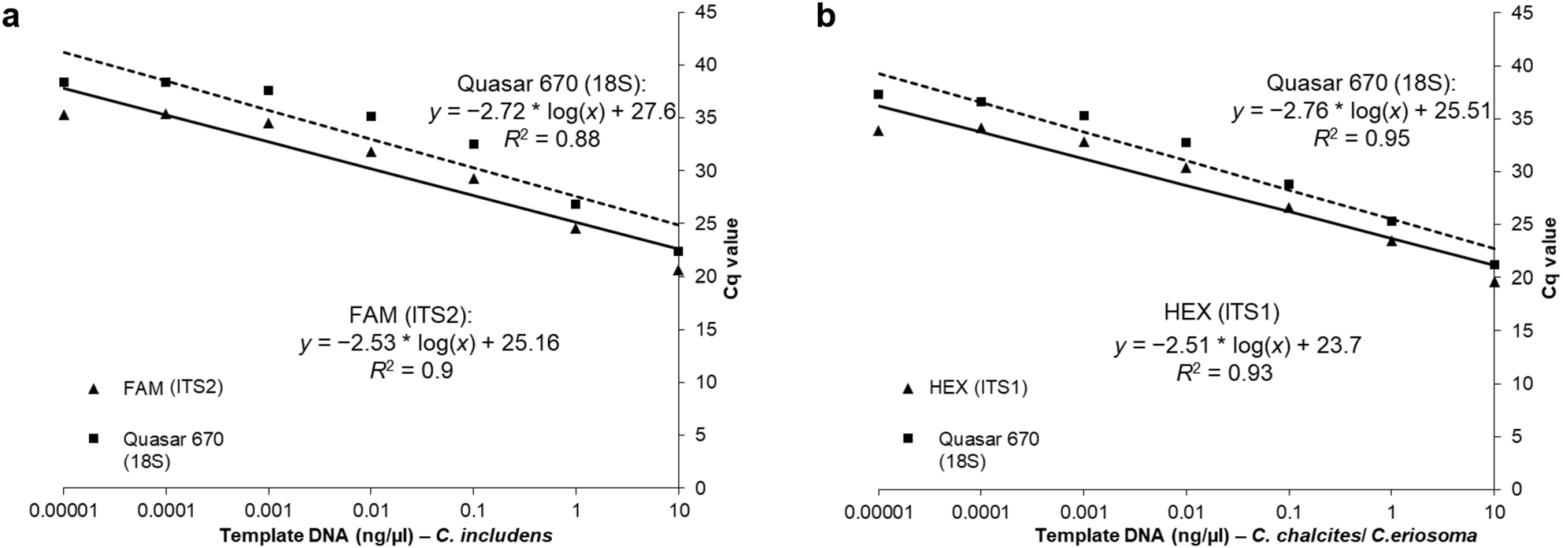
The standard curve of Cq values for the (**A**) *C. includens* (ITS2) and (**B**) *C. chalcites*/*C. eriosoma* (ITS1) primer and probe sets are shown for serial dilutions of purified DNA of each species run in the triplex real-time PCR assay. In each plot the triangles and solid line represent the diagnostic probe and the squares and dashed line represent the 18S control probe.

Standard curves were generated for both the FAM (Fig. 5A) and HEX (Fig. 5B) probes in conjunction with the Quasar 670 control probe. Generally, decreasing DNA concentration resulted in increased Cq for all concentrations tested for all three species. All replicates of the sensitivity assay were run with all three oligo sets present, indicating that multiplexing had little effect on assay sensitivity. Based on the standard curve, the FAM probe can detect *C. includens* DNA at ≥ 0.001 ng/µL (Fig. 5A) while the HEX probe can detect *C. chalcites* or *C. eriosoma* DNA at ≥ 0.0001 ng/µL (Fig. 5B).

False positive testing was carried out using 17 species (n = 67) of non-targets often collected in *Chrysodeixis* surveys or misidentified in interceptions at U.S. ports (Supplementary Table 2). These data were used to empirically determine the RFU threshold for each probe. Of these, 3 false positives occurred for the HEX probe and one false positive occurred for the FAM probe. Based on the end RFU of non-targets, the baseline threshold for all three probes was set to 1000 RFU. This threshold was used for all data reported. For the observed false positives, the ΔCq was > 8 cycles, which eliminated them from consideration as targets.

**Figure 6:**
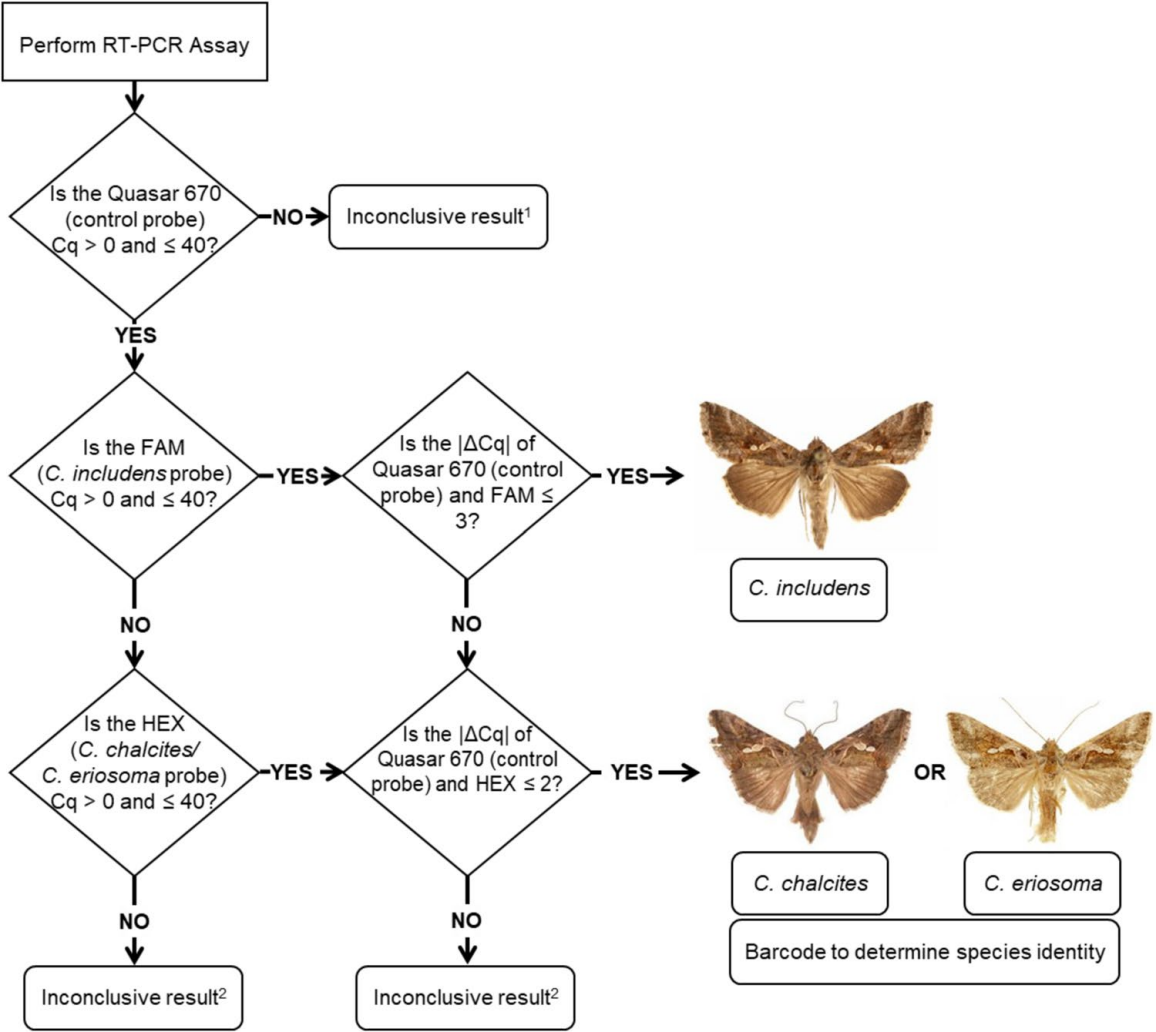
Rules for interpretation of real-time PCR results of suspected *C. includens, C. chalcites,* or *C. eriosoma* samples based on Cq values. ^1^Inconclusive result is likely due to insufficient DNA quantity or poor DNA quality. ^2^Inconclusive result is likely due to contamination, artifacts, or unsampled genotype. ^3^CO1 barcoding should be carried out to delineate between *C. chalcites* and *C. eriosoma* when needed.

Based on the ΔCq for all three probes, false positive testing, and sensitivity testing, a rule set was developed to determine the identity of specimens using the multiplex assay (Fig. 6). In order to separate *C. chalcites* and *C. eriosoma* from these results, the origin of the specimen could be considered in conjunction with CO1 barcoding.

## Discussion

Species delimitation has long been a source of debate among biologists (e.g.^49^). Despite the occasionally esoteric nature of these debates, functional differences are often associated with species designations, including key traits that can make one species more likely to be invasive than sibling lineages (e.g.^50,51^). Previously used biological and morphological methods are insufficient to determine the taxonomic status of *C. chalcites* and *C. eriosoma* as distinct species; however, there appear to be consistent differences in genetic markers. Using a genetic species concept requires discourse about which molecular markers provide the best signal for species recognition^52,53^. Among the most frequently used loci for metazoans are ITS1 and ITS2, with Coleman^22^ suggesting that ITS2 is always unique to species even though it is known to vary within species and even within individuals^26,27^. Between *C. chalcites* and *C. eriosoma* there are five SNVs and one indel in ITS1 and six SNVs in ITS2, both within the observed ribotypic variation for a single species^54^. When ITS1 was sequenced from more individuals, two of the SNVs proved to be variable loci with shared identity between the two species. Murillo et al.^36^ suggested that the molecular variation between CO1 barcodes of *C. chalcites* and *C. eriosoma* was around 1%, perhaps indicating synonymy. We found that variation between both species was around 1% for the ITS1, ITS2, and CO1 loci. This is in comparison to the variation between either of these species and *C. includens*, which was around 2.6% for the same loci. Despite the high level of similarity between *C. chalcites* and *C. eriosoma* in the phylogenetic analyses we conducted, both are monophyletic. Furthermore, the jackknife support separating the two species is 100% for both CO1 and the full 45S rDNA profiles (Fig. 2A, Supplementary Fig. 4), while posterior probability drops to 0.82 when only ITS1 is employed (Fig. 4A). When a single nucleotide polymorphism (SNP) analysis is run on *C. chalcites* and *C. eriosoma* CO1 data with a minimum variant frequency of 0.25, there are 12 SNPs at 6 loci that separate the two. In addition, species delimitation tests support the hypothesis that *C. chalcites* and *C. eriosoma* are separate species as they are both reciprocally monophyletic and appear to have independent evolutionary drive occurring within each grouping. Given these results, *C. chalcites* and *C. eriosoma* can be considered separate species under several different concepts^49^. The relatively small distance separating them may indicate that *C. chalcites* and *C. eriosoma* diverged only recently but have since remained separate as no evidence of incomplete lineage sorting or introgression was found in the rDNA or CO1 data. However, broad geographic sampling remains a challenge for both species as few *C. eriosoma* are available in museum collections or from interceptions and *C. chalcites* is mostly sampled from invasive populations. Conclusive evidence of their phylogenetic status will require more extensive genetic and geographic sampling.

The range of *C. eriosoma* extends from Hawaii to India across the Eastern Hemisphere, while the range of *C. chalcites* encompasses all of Africa to the Mediterranean region of Europe in the North and to India in the East^34,44^. This pattern of distribution is similar to that of other noctuid pests (e.g., *Spodoptera*). *Chrysodeixis chalcites* appears to be more invasive than *C. eriosoma*, as it has established populations outside of its natural range throughout Europe and North America while *C. eriosoma* has no known invasive populations. These populations of *C. chalcites* survive in greenhouses year-round and appear relatively isolated as the intra-species genetic distance is greater when compared to *C. includens* and *C. eriosoma* (Fig. 3A). Because most of the available barcode data for *C. chalcites* comes from specimens in isolated populations, the molecular variation within the natural range of *C. chalcites* is unknown, impacting its utility for species delimitation^55^. This is in contrast to *C. eriosoma*, which separates into two geographic haplogroups and shows low intra-species genetic distance (Figs. 2, 3A), as well as *C. includens*, which shows the lowest intra-species genetic distance of the three. *Chrysodeixis includens* is notedly panmictic because populations migrate north each year^28^. In a study of CO1 and CO2 haplotypes within Brazil, Silva et al.^56^ reinforced the finding of low variability within *C. includens* there, finding one dominant haplogroup throughout the country.

The biological and ecological factors that might be driving divergence between *C. chalcites* and *C. eriosoma* have not been adequately described. Adding to the difficulty in differentiating *C. eriosoma* from *C. chalcites*, Kostrowicki’s^40^ work elevating *C. eriosoma* from a synonym of *C. chalcites* did so based on wing coloration and genital morphology, neither of which are sufficient to reliably diagnose these lineages. More recent work on *C. eriosoma* and *C. chalcites* taxonomy by Twinkle et al.^42^ did little to interrogate the relationship between them. Because the single species hypothesis is supported as reciprocally monophyletic but randomly diverging, the clade could be considered one large Fisher–Wright population^48^. While it appears that the two species are distinct when they are considered separately, the same can be said for the distinct clades of *C. acuta* (Fig. 3; Supplementary Fig. 3). This is of note, because, similar to *C. chalcites*, the collection locations of *C. acuta* used in this analysis are not representative of its full range, a factor that is known to skew species delimitation analyses^55^.

In order to develop a diagnostic assay for these three species without generating complete genomes for each species, we chose to sequence 45S rDNA and use the ITS1 and ITS2 segments for identification. These high copy regions are particularly well-suited for molecular diagnostics because the sequences are abundant even in poorly preserved or environmentally degraded samples^25^. However, the use of PCR amplification to reduce sequencing input complexity and the nature of Nanopore sequencing, basecalling, and mapping all complicate classification of the complete ribotypic diversity of individuals by masking low frequency variation present in the DNA^26,57^. The minimap algorithm used for assembly allows for alignment of sequences that vary up to 15%^58^ which is important for use with noisy ONT data, but mapping reads to a reference sequence excludes low frequency variation from the final assembly. Sequence assembly was further complicated by the need for a progressive alignment technique to develop the *C. includens* and *C. chalcites* 45S rDNA profiles as well as the basecalling software available at the time these specimens were sequenced, both of which lead to errors in the final profiles. The *C. includens* data was basecalled with a high accuracy (HAC) model while *C. chalcites* was basecalled with the lower quality fast model. The poorer quality of the fast algorithm became apparent when *C. eriosoma* was later sequenced using a newer version of the HAC algorithm and revealed the sequencing and basecalling errors present through comparison between the two otherwise highly similar sequences. In addition, Nanopore technology has a known issue with reading and basecalling G bases and especially when sequencing a string (3 or more) of Gs^59^. Unsurprisingly, errors in G repeats were the issue most prevalent in the *C. chalcites* rDNA profile but were present in the profiles of all three species. Fortunately, ONT data allows for reanalysis of sequence data to improve call accuracy. As post processing for ONT improves, better characterization of ribotypic diversity may be possible with super accuracy basecalling models. In the future, the inclusion of more specimens in sequencing efforts should allow for improved detection of inter- and intraspecific rDNA variation and help to identify and eliminate artifacts that may disrupt efficient binding of the oligos designed for species detection when using a method such as ddPCR (see^11^). The advantage of reducing the sequence complexity using PCR, ONT, and the assembly and analysis techniques described here is to identify the most common motifs for species identification. This method allows rapid generation of an rDNA profile in a benchtop setting for poorly studied species for improved species diagnostics. Furthermore, we were able to identify ribotypes using LoFreq (Supplementary Table 1), the most common of which we were able to confirm by Sanger sequencing (Supplementary Fig. 1), that could interfere with efficient primer or probe binding and avoid them when developing the real-time PCR assay.

Due to the threat *C. chalcites* and *C. eriosoma* pose to U.S. agriculture, a probe that identifies specimens of both, as was developed here, is appropriate for screening suspects discovered in traps or intercepted at ports of entry. A specimen identified as either *C. chalcites* or *C. eriosoma* using the real-time PCR test must be verified through CO1 barcoding for reporting purposes. Conversely, *C. includens* is frequently intercepted at ports of entry to the U.S. Although it is present throughout the Americas, larvae are difficult to separate from other Plusiinae, so a probe that detects *C. includens* can be used to provide better identifications and reduce confusion with exotic pest species. Both diagnostic assays will also differentiate *Chrysodeixis* species from other commonly intercepted or trapped plusiines as the multiplex assay shows no off-target amplification of non-target species when the identification rules are followed.

Molecular diagnostics play an increasingly important role in species identification and phytosanitary security, including for species for which little to no validated reference DNA sequence data are available. This rDNA sequencing method can be easily adapted to other lineages by altering the primers due to the conservation of 18S and 28S between close relatives. In most cases, the ITS regions are excellent for species-level diagnostic assays and can be utilized for many types of samples and PCR modalities. More work, including expanded sampling and sequencing, is required to clarify the relationship between *C. chalcites* and *C. eriosoma* but, as they stand currently, our real-time PCR assay is sufficient to diagnose specimens for quarantine purposes, and will be validated for use by other laboratory facilities and also adapted for use in bulk screening for survey samples.

## Materials and methods

### Sample collection and species identification

Adult and larval specimens of *C. includens* and *C. chalcites* were intercepted on commodities at ports of entry and identified through CO1 barcoding^8^ using the primers LepF1 and LepR1 (^60^; Table 1), genitalic dissection^28^, morphological identification keys^61^, or a combination of these methods. Larval specimens comprising 58 *C. includens* and 8 *C. chalcites* individuals from various locations acquired through interceptions and surveys were used in this study. Adult specimens of *C. eriosoma* were collected in Hawaii using pheromone traps baited with *C. chalcites* lures (APHIS TL 17-065a) and preserved in ethanol for transport. Adults were also identified using CO1 barcoding with the primers LepF1 and LepR1 (Table 1). Five specimens of *C. eriosoma* were used for this analysis. Non-target testing was carried out using commonly captured plusiine species from the United States as well as intercepted plusiine larvae from other regions that are often mistaken for *Chrysodeixis* species. All specimens were identified by CO1 barcode, genitalic dissection, or both. Sanger sequencing, including CO1 barcoding, was carried out by the University of Chicago Comprehensive Cancer Center DNA Sequencing Facility using an Applied Biosystems 3730XL DNA sequencer (Applied Biosystems, Foster City, CA, USA). All target and non-target specimens used in this study are listed in Supplementary Table 2.

**Table 1:** Primers and probes used in this study.

Name	Description	Sequence	Tm^1^	Source
Lep_rDNA_F	rDNA forward primer	5′-CGATACCGCGAATGGCTC-3′	58.1	This study
Lep_rDNA_R	rDNA reverse primer	5′-GTTCAGGCATAATCCCGC-3′	55.9	This study
Includens_F	*C. includens* real-time forward primer	5′-CGCATATGACGGTGCTCC-3′	58.4	This study
Includens_P	*C. includens* real-time probe	5′-/56-FAM/ATTGGGTGC/ZEN/TTCGCGTTCGC/3IABkFQ/-3′	62.5	This study
Includens_R	*C. includens* real-time reverse primer	5′-CCACAAGCCGTTCGACAA-3′	56.3	This study
Chalcites_F	*C. chalcites*/*C. eriosoma* real-time forward primer	5′-CGCTGATCGTTCGTCTCG-3′	58.4	This study
Chalcites_P	*C. chalcites*/*C. eriosoma* real-time probe	5′-/5HEX/CGCGTTCGT/ZEN/TAATCCCGTCTCGT/3IABkFQ/-3′	66.6	This study
Chalcites_R	*C. chalcites*/*C. eriosoma* real-time reverse primer	5′-CGTCTACGAACACCGCGT-3′	58.4	This study
Chryso_ITS1_F	*Chrysodeixis* ITS1 forward primer	5′-GTTGCTGGGAAGTTGACCA-3′	57.5	This study
Chryso_ITS1_R	*Chrysodeixis* ITS1 reverse primer	5′-GCCCTCAGACAGGAGTGG-3′	60.8	This study
RT-18S-F2	18S real-time control forward primer	5′-ACCGCCCTAGTTCTAACCGTAAA-3′	62.9	^23^
RT-18S-P2	18S real-time control probe	5′-/Quasar670/TGTCATCTAGCGATCCGCCGA/BHQ/-3′	63.5	^23^
RT-18S-R2	18S real-time control reverse primer	5′-CCGCCGAGCCATTGTAGTAA-3′	63.2	^23^
LepF1	CO1 barcoding forward primer	5′-ATTCAACCAATCATAAAGATATTGG-3′	57.6	^60^
LepR1	CO1 barcoding reverse primer	5′-TAAACTTCTGGATGTCCAAAAAATCA-3′	60.1	^60^

^1^Tm in °C.

### DNA extraction

For applications that did not require high quality and concentration of DNA (e.g., DNA barcoding), a Qiagen DNeasy Blood and Tissue kit (Qiagen, Hilden, Germany) was used following manufacturer’s instructions. For applications that required higher quality and concentration DNA (e.g., long range PCR), the Lucigen MasterPure Complete DNA and RNA Purification Kit (LGC Ltd, Teddington, United Kingdom) was used following manufacturer’s instructions with an added one-hour incubation at − 20 °C prior to precipitation to increase DNA yield (see^10^). All specimens were stored in ethanol before use. For larval specimens, a small section was taken from the posterior end of each and ground in a 1.5 mL tube with a pestle then incubated overnight in lysis buffer with the remaining portion of the extraction techniques carried out the next day. For adult specimens, legs were removed and dried of residual ethanol then ground in a 1.5 mL tube using 1.4 mm silica/zirconia beads in a Mini-Beadbeater (BioSpec Products, Bartelsville, OK, USA). DNA extracted by both methods was eluted in Buffer EB (Qiagen) to ensure compatibility with PCR and sequencing. DNA concentration was quantified using both a NanoDrop 2000 spectrophotometer (Thermo Fisher Scientific, Waltham, MA, USA) and a Qubit 4.0 Fluorometer (Invitrogen, Waltham, MA, USA) using the Qubit dsDNA HS Assay Kit (Invitrogen) following manufacturer’s instructions. All DNA was stored at − 20 °C until use.

### Long-range PCR

Due to the lack of genomic sequence data available for any *Chrysodeixis* species, we designed a set of general primers for lepidopteran rDNA (Table 1) using an alignment (Supplementary File 2) of the sequences listed in Supplementary Table 3. The primers amplify nearly the entire rDNA region from the 5′ end of 18S to the 3′ end of 28S, a length typically between 6500–7000 bp in lepidopteran species (see accessions from Supplementary Table 3). Primers were designed using Primer 3 v0.4.0^62,63^ with the following settings: divalent cations = 3.8 mM; monovalent cations = 50 mM; dNTPs = 0.8 mM with the SantaLucia^64^ formula for salt correction and thermodynamic parameters. Primers were manufactured by IDT (Integrated DNA Technologies Inc., Coralville, IA, USA). OligoCalc^65^ was used to calculate salt-adjusted melting temperatures. Individual, ethanol-preserved, larval specimens of *C. includens* and *C. chalcites* that had not been previously used for DNA extraction were used to obtain DNA for long-range PCR using the Lucigen MasterPure DNA and RNA Purification Kit and identified by CO1 barcoding. The same was done using two legs from an adult *C. eriosoma* specimen. A total of 30 ng of DNA was used for amplification with the Takara LR Taq kit (TAKARA BIO INC., Kusatsu, Shiga, Japan) using 600 nM of each primer (Table 1), 5.0 µL of buffer, 8.0 µL of dNTP mix, and 0.5 µL of Taq. The PCR program used was as follows: 95 °C for 3 min followed by 35 cycles of 98 °C for 30 s, 51 °C for 30 s, 68 °C for 10 min; and final extension step at 72 °C for 10 min. All PCR was carried out using a Bio-Rad C1000 Touch thermocycler (Bio-Rad Laboratories Inc., Hercules, CA, USA). After thermocycling, PCR products were run on a 0.5% agarose gel at 50 V for 3 h and imaged on a UVP GelSolo transilluminator (Analytik Jena AG, Jena, Germany). DNA from *Helicoverpa armigera* and *H. zea* were also used as PCR controls to ensure the primers were working as intended. PCR clean-up was done by bead purification using AMPure XP paramagnetic beads (Beckman Coulter Inc., Brea, CA, USA) following the manufacturer’s workflow. The fragments were quantified on a Qubit 4.0 Fluorometer as outlined above and stored at − 20 °C until use.

### Long-read sequencing and data analysis

Sequencing of the long-range PCR products was carried out using a MinION Mk1C with MinION R9 flow cells (Oxford Nanopore Technologies; Oxford, UK). A Ligation Sequencing kit (SQK-LSK110, Oxford Nanopore Technologies) was used for library preparation following manufacturer’s instructions. Nanopore sequencing reactions were run for 24 h and were live basecalled by the Mk1C or basecalled post-run in Guppy v. 6.1.2 (Oxford Nanopore Technology). Separate library preparations and sequencing reactions were carried out for each species.

Following sequencing, Epi2Me v3.4.2 (Oxford Nanopore Technologies) was used to determine the identity of the DNA sequences in each reaction. Sequences from the pass files generated during basecalling were then imported to Geneious Prime 2021 or 2022 (https://www.geneious.com) as lists of sequences which were broken into 136 pools of 4000 sequences each for *C. includens* and 1825 pools of 4000 sequences each for *C. chalcites* and 1821 pools of 3500–4000 sequences each for *C. eriosoma*. From these, fragments of the predicted size of rDNA from each species (based on gel electrophoresis of the PCR product) were selected, typically accounting for around 10% of the total sequence data for each *C. chalcites* and *C. eriosoma* and around 25–30% of total sequence data for *C. includens*. Size-sorted long-read sequence data for each species is available in GenBank under the BioProject ID PRJNA951779. For *C. chalcites* and *C. includens*, initial sequences were obtained by progressively aligning the highest quality sequences from the first sets of pass files using MUSCLE^66^. The remaining raw sequences were mapped to the MUSCLE-derived consensus sequence using the Minimap2 plug-in^58^ for Geneious Prime using the Pac-Bio/Oxford Nanopore data type and default settings. To obtain a final rDNA profile for each species, the consensus sequences from each pass file were aligned to each other using MUSCLE and MAFFT v 7.450^67,68^ using default settings for comparison. As *C. eriosoma* was sequenced last, pass files from this sequencing run were mapped to the final consensus sequence for *C. chalcites* using Minimap2 with the above settings. Final assemblies of the 45S rDNA region generated for this study can be found in GenBank under the accession numbers OQ829604-OQ829607. Ambiguities remaining in the final alignment were determined to be either sequencing errors or ribotypes based on the quality and type of each ambiguity (for example gaps or Ns were considered sequencing errors, ambiguous calls where sequence variations at a single point were roughly split between two bases across consensus sequences were considered ribotypes). Ambiguities representing ribotypes were confirmed using LoFreq^45^ roughly following the method from Sultanov and Hochwagen^69^. In short, a subset of the size selected sequence data for each species was realigned to its respective final rDNA profile using Minimap2 with the Oxford Nanopore data type and default settings. Alignments were exported as .bam files and variants and indels for each were called with LoFreq. The resultant output.vcf files were imported into Geneious and mapped onto the rDNA profiles for each species.

From the LoFreq results, variants that were present in ≥ 50% of the sequences were verified using Sanger sequencing to look for heterozygous peaks (Supplementary Table 4). In total, four sets of primers were made to investigate four possible ribotype loci in *C. chalcites* and three sets of primers were designed to investigate four possible ribotype loci in *C. includens* (Supplementary Table 1). For each species, a final consensus was generated that included all pass files and ambiguities that represented only biologically inferred ribotypes corrected to reflect Sanger sequencing results where applicable (Supplementary File 1). Final rDNA profiles for each species were aligned with each other for use in designing a real-time PCR assay for each species.

### Phylogenetic analyses

To determine the phylogenetic relationships between the three species of interest, available CO1 data from the Barcode of Life Database (BOLD) were downloaded. Accession numbers and collection locations for the sequence data that was used is given in Supplementary Table 5. Barcodes generated for this study are available in GenBank under the accession numbers OQ732763-OQ732776. After quality control, CO1 sequences were aligned for all three species using MAFFT v7.450 with default settings. All sequences were trimmed to the same length (494 bp). One sequence from *Helicoverpa zea* or three sequences from *H. armigera* (Supplementary Table 5) were also included as outgroup taxa. A Bayesian tree was generated using the MrBayes plugin^70,71^ for Geneious Prime using the GTR substitution model, MCMC chain length of 1,100,000, subsampling frequency of 200, and a 100,000 tree burn-in with an unconstrained branch length prior. In addition, a Neighbor Joining (NJ) tree was resolved using the Tamura–Nei distance model^72^ with no set outgroup and 1000 jackknife replicates to assess branch support.

In addition to CO1, we designed primers (using the above parameters) to amplify the ITS1 region of *Chrysodeixis* to sample the resolution of these clades using the target sequence for the real-time PCR assay to detect *C. chalcites*. The forward primer placement is within the 3′ portion of 18S and the reverse primer placement is within the 3′ portion of 5.8S to allow for some universality of the primers to other Lepidoptera (Table 1). The ITS1 region was then amplified from a subset of our archive of *Chrysodeixis* larvae and adults (3 specimens of *C. includens*, 11 specimens of *C. chalcites*, and 5 specimens of *C. eriosoma*) as well as from *H. zea* to serve as an outgroup. PCR was carried out as described above for CO1 but with 500 nM of primers Chryso_ITS1_F and Chryso_ITS1_R (Table 1) and the following thermocycling program: 95 °C for 3 min; 40 cycles of 95 °C for 30 s, 58 °C for 30 s, 72 °C for 1 min; and 72 °C for 5 min. ITS1 sequences generated for this paper are available in GenBank under the accession numbers OQ780372-OQ780392. A phylogenetic tree was generated from this data as described for CO1 with Bayesian analysis as described above.

### Species delimitation

Relationships within and between the three *Chrysodeixis* species were assessed using both CO1 and ITS1 sequence data. The TCS statistical parsimony analysis was used in PopART v1.7 (Population Analysis with Reticulate Trees^73^) to visualize differences between species using both datasets as well as between species using rDNA profiles including ribotypic diversity. Species delimitation analysis was carried out using the Species Delimitation Plug-In for Geneious Prime^46^ by comparing groupings of all three species along with closely related *C. acuta* due to its similarly broad geographic distribution and abundant available CO1 sequence data. For this analysis, a subset of the previously used CO1 sequences for each species from BOLD and GenBank (Supplementary Table 5) were used to resolve an NJ tree as described above. The size of each clade was reduced to avoid a calculation artefact that exists in the Species Delimitation program (see^46,48^ for details). Each of the resultant internal clades was selected to assess the boundaries of current *Chrysodeixis* species.

### Real-time PCR

A set of primers and a probe were designed for each clade of interest, *C. includens* and *C. chalcites*/*C. eriosoma*, based on the rDNA sequence data generated above (Table 1; Supplementary Fig. 5). Regions of ITS were chosen based on sequence dissimilarity between the two clades and to other Lepidoptera as well as to avoid areas of intragenomic sequence variation within the individuals sequenced. The regions chosen for identification were within ITS2 for *C. includens* (Supplementary Fig. 5A) and within ITS1 for *C. chalcites*/*C. eriosoma* (Supplementary Fig. 5B).

All real-time PCR was performed on a Bio-Rad CFX96 Touch Real-time PCR System (Bio-Rad Laboratories Inc.). The assays were designed to be run in triplex with both diagnostic probes and a control probe (Table 1) to be used simultaneously. The control assay was implemented as described in Barr et al.^23^. After optimization, the final real-time PCR mix included 10 µL iTaq Universal Probes Master Mix (Bio-Rad Laboratories Inc.), 350 nM of *C. includens* primers (includens_F and includens_R), 175 nM of *C. includens* probe (includens_P), 300 nM of *C. chalcites* primers (chalcites_F and chalcites_R), 200 nM *C. chalcites* probe (chalcites_P), 500 nM 18S control primers (RT_18S_F2 and RT_18S_R2) and 400 nM 18S control probe (RT_18S_P2) with water to complete the dilution of the master mix and 1 µL of DNA of varying concentration or additional water for no tissue controls (NTC). The thermocycling program used is as follows: 3 min at 95 °C initial denaturation followed by 40 cycles of 95 °C for 20 s and 59 °C for 30 s with data capture at the end of each cycle. For all reactions, 96-well, thin-walled, white well, hard-shell PCR Plates (Bio-Rad Laboratories Inc.) were used with optically clear Microseal ‘B’ seals (Bio-Rad Laboratories Inc.). Assay specificity was tested using 17 other commonly trapped or intercepted Plusiine species (larvae and adult) that may be visually mistaken for *C. includens*, *C. chalcites*, or *C. eriosoma* (n = 67, Supplementary Table 2). Threshold values for each primer/probe set were determined by running the assay on 54 specimens of *C. includens*, 35 specimens of *C. chalcites*, and 5 specimens of *C. eriosoma*. The baseline threshold was manually set to 1000 RFU for each probe.

### Sensitivity analysis for real-time PCR

Sensitivity of the assay was determined by running 3 technical replicates of serial dilutions of DNA from *C. includens*, *C. chalcites*, and *C. eriosoma* ranging in concentration from 10 ng/µL to 1 × 10^−6^ ng/µL. The assay was performed with the multiplex mix of the *C. includens*, *C. chalcites*, and 18S control primer/probe sets. The results were adjusted to reflect the empirically determined RFU threshold and the Cq values were averaged and compared to DNA concentration on a logarithmic scale to determine the slope, y-intercept, and correlation effects of DNA concentration on assay sensitivity following Barr et al.^23^.

### Supplementary Information

Supplementary Information 1.

Supplementary Information 2.

Supplementary Information 3.

## Data Availability

Size-sorted, basecalled, pass files from ONT sequencing can be found under the BioProject number PRJNA951779 for each species at https://dataview.ncbi.nlm.nih.gov/object/PRJNA951779?reviewer=vrpekj27ut9qv2i5qhe3f6b855. Sanger results for CO1 and ITS1 can be found in GenBank under accessions OQ732763-OQ732776 and OQ780372-OQ780392 respectively and with the Supplementary Information. Electropherograms for Sanger ribotype checks are available as Supplemental Fig. 1. All other datasets generated during the current study are available from the corresponding author on reasonable request.
