# Supplementary material for: Ultra-deep sequencing of 45S rDNA to discern intragenomic diversity in three Chrysodeixis species for molecular identification
Source: Sci Rep. 2023 Aug 10;13:13017. doi: 10.1038/s41598-023-39673-7 (PMC10415407; doi:10.1038/s41598-023-39673-7)
Supplement: Supplementary file 1 — Supplementary Information 1. [file 41598_2023_39673_MOESM1_ESM.pdf]

Ultra-deep sequencing of 45S rDNA to discern intragenomic diversity in three *Chrysodeixis* species for molecular identification

Frida A. Zink[1], Luke R. Tembrock[1,*], Alicia E. Timm[1], Todd M. Gilligan[2]

[1]Department of Agricultural Biology, Colorado State University, Fort Collins, CO, United States
[2]USDA-APHIS-PPQ-Science & Technology, Pest Identification Technology Laboratory, Fort Collins, CO, United States
[*]Corresponding author: tembrock@colostate.edu

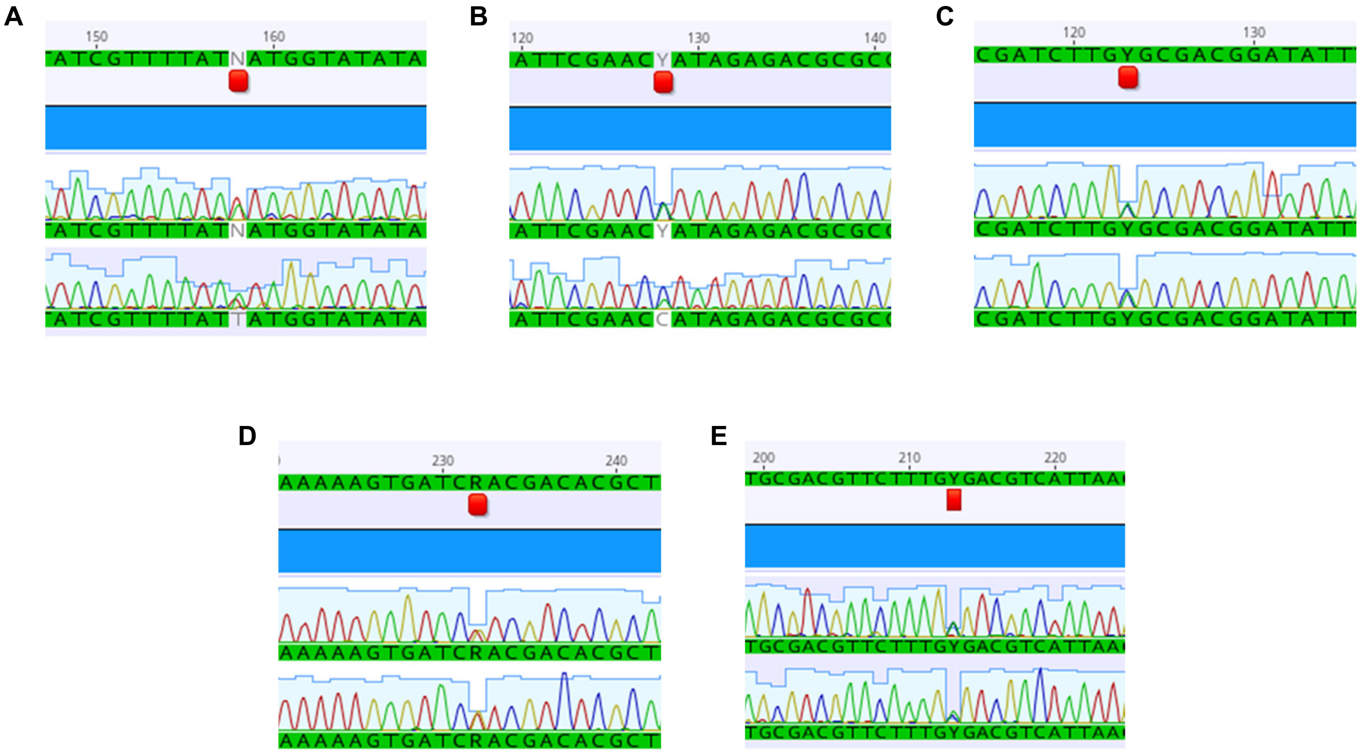

Supplementary Figure 1: Sanger sequencing results confirming ribotypes from ONT data for *C. chalcites* (A) and *C. includens* (B-E). Ribotypes are indicated by red boxes and double peaks are evident in electropharograms below. In all cases, the first sequence at the top is the consensus, the middle sequence is the forward strand, and the bottom sequence is the reverse strand.

A

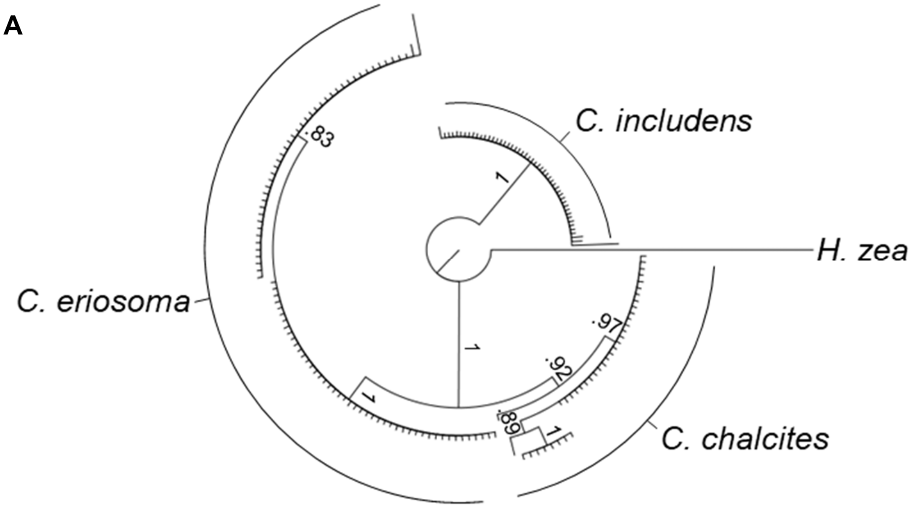

B

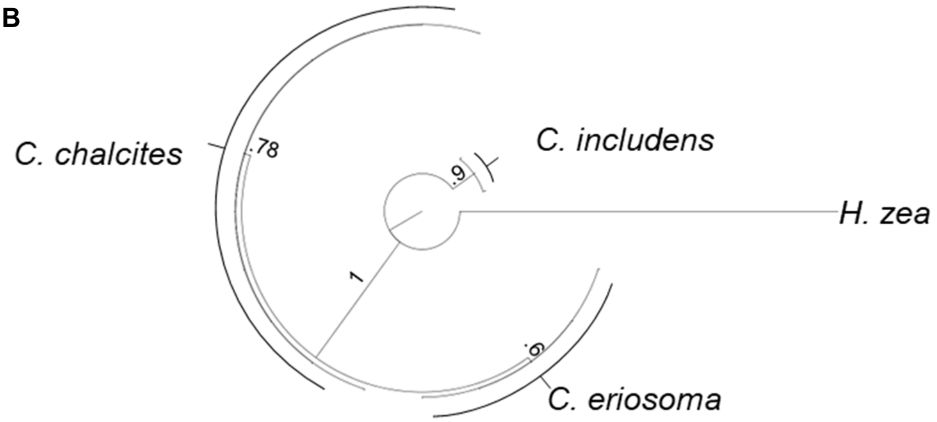

Supplementary Figure 2: Bayesian Inference trees for the three *Chrysodeixis* species show *C. includens* as parent to a clade with both *C. chalcites* and *C. eriosoma*. The same relationships are supported using both CO1 (A) and ITS1 (B) sequence data. Branch labels represent posterior probability. *Helicoverpa zea* is set at the outgroup.

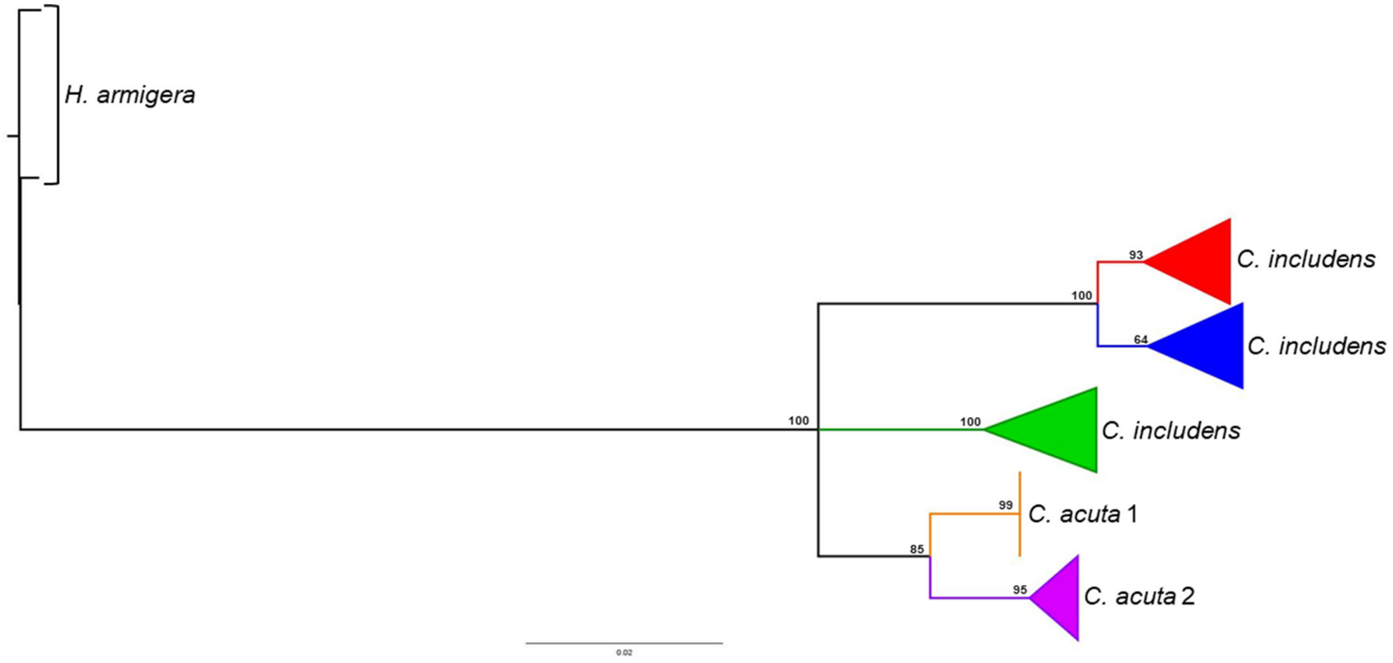

Species	Closest	Intra Dist	Inter Dist	Intra/Inter	Rodrigo's P	Rosenberg's P_{AB}
1	2	0.003	0.012	0.27	0.05	$3.3x10^{-23}$
2	1	$6.65x10^{-4}$	0.012	0.05	0.05	$3.3x10^{-23}$
3	4	$7.72x10^{-4}$	0.042	0.02	1	$3.0x10^{-32}$
4	5	0*	0.023	0	NA*	0.01
5	4	0.005	0.23	0.20	0.05	0.01

Supplementary Figure 3: Species delimitation analysis was carried out following the hypothesis that *C. acuta* is two species based on branch support within the clade. Clades representing putative species are collapsed at the most recent common ancestor. Branch labels indicate jackknife support. *Helicoverpa armigera* was set as the outgroup.

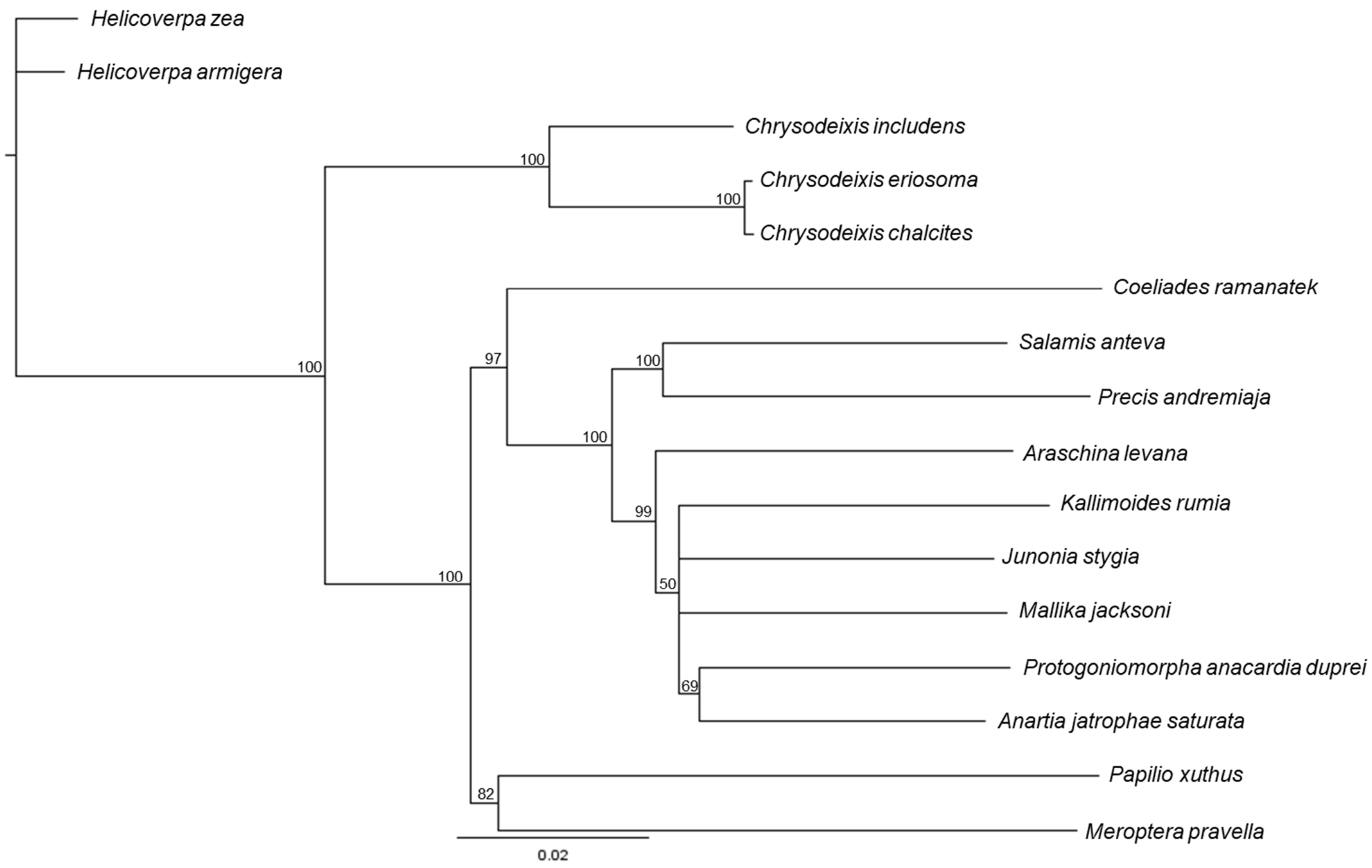

Supplementary Figure 4: Neighbor-Joining Tree of 45S rDNA sequences of Lepidoptera used for primer design along with *Chrysodeixis* rDNA profiles developed for this paper. Branch labels indicate jackknife support. No outgroup was selected.

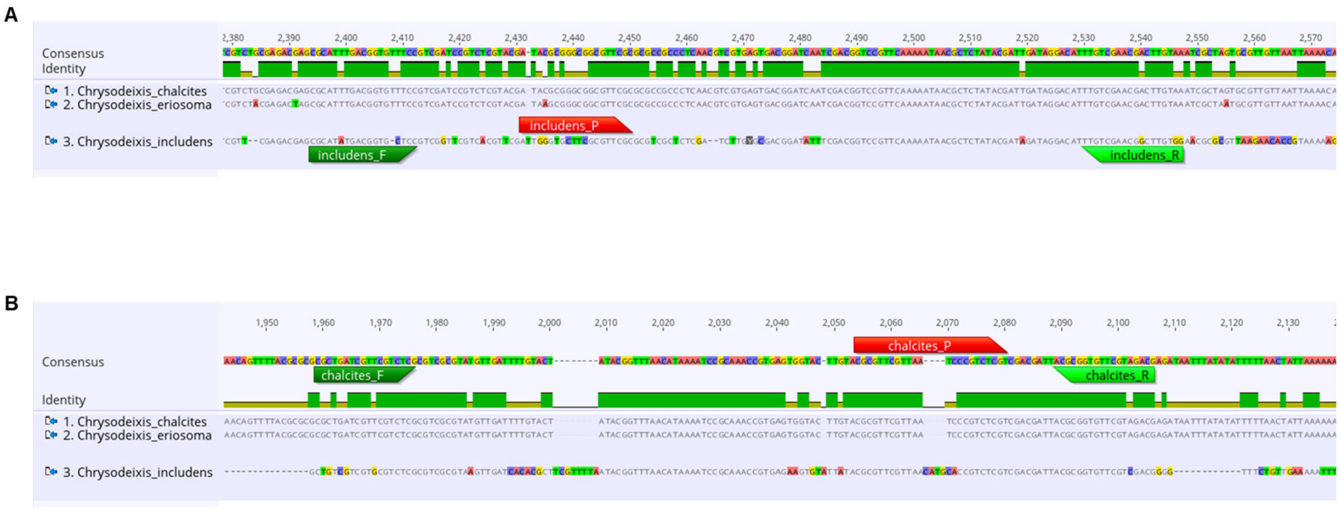

Supplementary Figure 5: A schematic of the alignment of ITS2 (A) showing the primer and probe binding sites for the *C. includens* diagnostic real-time PCR assay and ITS1 (B) showing the primer and probe binding sites for the *C. chalcites*/ *C. eriosoma* diagnostic real-time PCR assay. Forward primer sites are marked with dark green, reverse primer sites are marked with light green, and probe sites are marked with red.

Supplementary Table 1: ambiguities in ONT data, putative ribotypes identified in last column

Species and locus	A	C	G	T	-	N	W	Y	R	K	Column1
C. includens 585					135	1					
C. includens 1127						136					
C. includens 1946		17						119			*C. includens* putative ribotype 1
C. includens 2121	135								1		
C. includens 2125					2	134					
C. includens 2126				134			2				
C. includens 2456				6				130			*C. includens* putative ribotype 2
C. includens 2565			51						85		*C. includens* putative ribotype 3
C. includens 3050					135	1					
C. includens 4533								136			*C. includens* putative ribotype 4
C. includens 6320					135	1					
C. includens 6372					135	1					
C. chalcites 109			1797						1		
C. chalcites 321	1797				1						
C. chalcites 322		1797						1			
C. chalcites 330			1796			1			1		
C. chalcites 424			1796			2					
C. chalcites 894			148		999	651					
C. chalcites 1114					1573	225					
C. chalcites 1609		301			999	498					
C. chalcites 1628		126			574	1082		16			*C. chalcites* putative ribotype 1
C. chalcites 2900				1136	374	288					
C. chalcites 2925			1792			6					
C. chalcites 3534			1797			1					
C. chalcites 3556		1785				13					
C. chalcites 3557			1791			7					
C. chalcites 3807				1796				2			
C. chalcites 3938						712				1086	*C. chalcites* putative ribotype 2
C. chalcites 4364	1797					1					
C. chalcites 4802	1						1797				*C. chalcites* putative ribotype 3
C. chalcites 4894		1797				1					
C. chalcites 4921			1779			19					
C. chalcites 5222		1746				52					
C. chalcites 5340			1784						14		*C. chalcites* putative ribotype 4
C. chalcites 5593			1796			2					
C. chalcites 6063	1797						1				
C. chalcites 6064			1797						1		
C. chalcites 6143			1797			1					
C. eriosoma 1120			1		1572	228					
C. eriosoma 3550		1			1419	373					
C. eriosoma 4874		1800						1			
C. eriosoma 5335			1800						1		
C. eriosoma 5732			1800						1		

Supplementary Table 2: Specimens used in this study

Taxon	Location	Trapping method	Life stage	n
Chrysodeixis includens	South America, North America	Interception	Larvae	57
Chrysodeixis chalcites	Europe, North America	Interception, pheromone	Larvae, adult	36
Chrysodeixis eriosoma	North America	Pheromone	Adult	5
Ctenoplusia oxygramma	North America	Interception, pheromone	Larvae, adult	43
Rachiplusia ou	North America, South America	Interception	Larvae	2
Autoplusia egena	North America	Pheromone	Adult	1
Autoplusia metallica	North America	Pheromone	Adult	1
Syngrapha viridisigma	North America	Pheromone	Adult	1
Syngrapha sackenii	North America	Pheromone	Adult	1
Syngrapha celsa	North America	Pheromone	Adult	1
Plusia putnami	North America	Pheromone	Adult	1
Trichoplusia ni	North America	Pheromone	Adult	2
Autographa ampla	North America	Pheromone	Adult	2
Autographa pseudogamma	North America	Pheromone	Adult	2
Autographa mappa	North America	Pheromone	Adult	2
Autographa gamma	Europe	Pheromone	Adult	2
Autographa precationis	North America	Pheromone	Adult	1
Anagrapha falcifera	North America	Pheromone	Adult	2
Syngrapha angulidens	North America	Pheromone	Adult	2
Autographa californica	North America	Pheromone	Adult	2
Spodoptera albula	North America	Interception	Larvae	2
Rachiplusia nu	South America	Interception	Larvae	1
Helicoverpa zea	North America	Pheromone	Adult	3

Supplementary Table 3: Sequence data used for rDNA alignment and primer design

Species	Location	Sequence	GenBank Accession
Helicoverpa zea	Lab reared	Genome	PRJNA788876
Helicoverpa armigera	Lab reared	rDNA	OQ829604
Anartia jatrophae saturata	Dominican Republic	rDNA	MT742579.1
Junonia stygia	Central African Republic	rDNA	MN623382.1
Protogoniomorpha anacardii duprei	Madagascar	rDNA	MT702383.1
Mallika jacksoni	Uganda	rDNA	MT704831.1
Kallimoides rumia	Nigeria	rDNA	MT704380.1
Salamis anteva	Madagascar	rDNA	MH917709.1
Coeliades ramanatek	Madagascar	rDNA	MT859413.1
Papilio xuthus	Japan	rDNA	AB674749.1
Araschnia levana	Belgium	rDNA	MT750296.1
Precis andremiaja	Madagascar	rDNA	MH917708.1
Meroptera pravella	Canada	rDNA	MF073208.1

Supplementary Table 4: Sanger sequencing primers to confirm ribotypes for C. chalcites and C. includens

Name	Description	Sequence	Tm1	Source
chalcites_1628F	Forward primer for *C. chalcites* putative ribotype 1	5’-TGGTGGAGCGATTTGTCTG	55.2	This study
chalcites_1628R	Reverse primer for *C. chalcites* putative ribotype 1	5’-TCCCCTACGGAAACCTTGT	55.7	This study
chalcites_3938F	Forward primer for *C. chalcites* putative ribotype 2	5’-CCAACGTCTACGCCACAG	55.9	This study
chalcites_3938R	Reverse primer for *C. chalcites* putative ribotype 2	5’-TTCAAATCGACGCCAGCT	54.8	This study
chalcites_4802F	Forward primer for *C. chalcites* putative ribotype 3	5’-CGGTGCAGATCTTGGTGG	56.2	This study
chalcites_4802R	Reverse primer for *C. chalcites* putative ribotype 3	5’-GCTGCGGATATGGGTACG	55.7	This study
chalcites_5340F	Forward primer for *C. chalcites* putative ribotype 4	5’-ATTGGCTCTGAGGACCGG	57.1	This study
chalcites_5340R	Reverse primer for *C. chalcites* putative ribotype 4	5’-CCATTCATGCGCGTCACT	56.1	This study
includens_1946F	Forward primer for *C. includens* putative ribotype 1	5’-AGTCGTAACAAGGTTTCCGT	54	This study
includens_1946R	Reverse primer for *C. includens* putative ribotype 1	5’-TCACACTATGACGCGCAG	55	This study
includens_2456F	Forward primer for *C. includens* putative ribotypes 2 and 3	5’-CTGAGGGCCGGCTGTATA	56.5	This study
includens_2565R	Reverse primer for *C. includens* putative ribotypes 2 and 3	5’-CCGCCTACTCGACCCTTA	56.1	This study
includens_4533F	Forward primer for *C. includens* putative ribotype 4	5’-CGCTGTGGGATGAACCAA	55.5	This study
includens_4533R	Reverse primer for *C. includens* putative ribotype 4	5’-AAACACGCCACATCGACA	55	This study

Supplementary Table 5: BOLD sequences used for phylogenetics and phylogeography

Species	Location	3-letter code	Gene	BOLD Accession
Chrysodeixis includens	Canada	CAN	CO1	SMTPO4162-15, SMTPR191-16, SMTPR7089-16, XAB463-04, XAH608-05, RDLQ426-07
	Argentina	ARG	CO1	ARMOT129-12, ARMOT187-12, ECPD519-14, LEPPA1129-14, LEPPA1141-14, MOTAR192-12, MOTAR197-12
	Costa Rica	CRI	CO1	BLPAA312-06, BLPAA7798-17, BLPAA727-06, BLPAA3862-17, BLPAA848-06, LOCRA264-06
	Brazil	BRA	CO1	CRYFC001-19, LEMMZ135-10, LEMMZ136-10, GBGL12665-13, GBGL12666-13, GBGL12667-13, GBGL12668-13
	USA	USA	CO1	LGSMG611-07, LGSMG612-07, LGSMG613-07, LNC849-06, LNCB071-06, LILLA916-11, LILLA958-11, LILLB064-11, LOCBF3743-14, LOCBF4241-14, LOCBF4242-14, LOFLB770-06, LPOKA1032-09, LPOKA343-08, LPOKA567-09
	Peru	PER	CO1	LNAUP106-13, LNAUP107-13, NOCJB052-08
	Mexico	MEX	CO1	LPYPB275-08, GMMCS002-15
	Bolivia	BOL	CO1	NOCJB053-08
Chrysodeixis chalcites	Austria	AUT	CO1	ABOLB389-15, LEASS10091-17, NOENO063-17
	Israel	ISR	CO1	BCMI041-11, BCMI449-11
	Germany	DEU	CO1	GBLAC213-13
	Turkey	TUR	CO1	GBMNA46352-19, TRLEP109-13
	India	IND	CO1	GBMNC56439-20, GBMNC56440-20
	Spain	ESP	CO1	GWORR870-10, IBLAO1037-14
	Italy	ITA	CO1	GWOSA330-10, GWOTF359-12
	Greece	GRC	CO1	LEASW1143-20
	Canada	CAN	CO1	LMHRG044-06, LMHRG045-06, RDNMH238-09, RDNMK153-11, RDNMK154-11, RDNMK155-11, RDNMK156-11, TZBCA102-06
	Norway	NOR	CO1	LONG3858-16
	Portugal	PRT	CO1	PHLPM056-11, RDNMK227-11
	Seychelles	SYC	CO1	PMANL4409-15
Chrysodeixis eriosoma	India	IND	CO1	GBGL34876-19, GBGL34877-19, GBMNC56451-20, GBMNC56452-20, GBMNC56453-20, GBMNC56454-20, GBMNC56455-20, GBMNC56456-20, GBMNC56457-20, GBMNC56458-20, GBMNC56459-20, GBMNC56460-20, GBMNC56461-20,
	Australia	AUS	CO1	ANIC596-06, ANICL315-10, ANICL316-10, ANICL317-10, LOQ259-04, LOQC055-05, LOQC397-05, LOQC500-05, LOQT511-06, LOQTE019-09, WALPI765-18, NSWBB637-08, NSWBB738-08, NSWBB917-08, NSWHH459-09, NSWHM625-11, LNSWB397-05, LOTSD356-08, WALPA5781-13, WALPA5783-13, WALPA5784-13, WALPA5785-13, WALPA5787-13
	USA	USA	CO1	CNCLA570-13, GBGL17356-15
	Bangladesh	BNG	CO1	GBMCE3787-15
	Indonesia	IDN	CO1	LEPKB156-10
	Malaysia	MYS	CO1	LEPMY1417-15
	French Polynesia	PYF	CO1	PMANL4352-15, LNAUY2100-19, PMANL4350-15, SYC2452-14, SYC3530-14, SYC4757-14, SYC4759-14, SYC2649-14, SYC4424-14, SYC4593-14, SYC4973-14, SYC4984-14, SYC5146-14, SYC5790-14, SYC5858-14, SYC5859-14
	China	CHN	CO1	LON4586-16, QMA531-13, QMA3113-13
	Papua New Guinea	PNG	CO1	LPNGD696-06, LPNGD699-06, LPNGD700-06, LPNGD698-06, YAWAN982-14
	Pakistan	PAK	CO1	MAMOT111-10, MAMOT2830-12, MAMOT2846-12, MAMOT2817-12, MAMOT2787-12, MAMOT2788-12, MAMOT2834-12, MAMOT112-10, MAMOT491-10, MAMOT493-10, MAMOT686-10, MAMOT488-10, PMNHD1397-15, PMNHD1399-15
	New Zealand	NZL	CO1	NZLEP347-20
Chrysodeixis acuta	India	IND	CO1	NC_066154.1*, OL892047.1
	Australia	AUS	CO1	ANICL306-10, ANICL307-10
	Kenya	KEN	CO1	GMKMW195-15
	South Africa	ZAF	CO1	GWOTG677-12

	Nigeria	NGA	CO1	PMANK908-08
	Gabon	GAB	CO1	MGABA104-10
Helicoverpa armigera	Peru	PER	CO1	MK779611.1*
	Germany	DEU	CO1	GU654969.1*
	Finland	FIN	CO1	JF853867.1*

*indicates Genbank accession instead of BOLD accession
