# Supplementary material for: Ultra-deep sequencing of 45S rDNA to discern intragenomic diversity in three Chrysodeixis species for molecular identification
Source: Sci Rep. 2023 Aug 10;13:13017. doi: 10.1038/s41598-023-39673-7 (PMC10415407; doi:10.1038/s41598-023-39673-7)
Supplement: Supplementary file 2 — Supplementary Information 2. [file 41598_2023_39673_MOESM2_ESM.pdf]

```
Consensus          CGATACCGCGAATGGCTCAATATATCAGTTTTGGTTCCTTAGATCTTACTCAGTTACTTG    60
C. eriosoma rDNA   ............................................................    60
C. chalcites rDNA  ............................................................    60
C. includens rDNA  ............................................................    60

Consensus          GATAACTGTGGTAATTCTAGAGCTAATACATGCAATCAGAACTCTGACCAGTGATGGGAT   120
C. eriosoma rDNA   ............................................................   120
C. chalcites rDNA  ............................................................   120
C. includens rDNA  ............................................................   120

Consensus          GAGTGCTTTTATTAGATCAAAACCAATCGACGGAGGGCCACGCGTCYKAAGTCGTTAATT   180
C. eriosoma rDNA   ..............................................CT............   180
C. chalcites rDNA  ..............................................CT............   180
C. includens rDNA  ..............................................TG............   180

Consensus          TTGATGAATCTGGATAACTTTTGCCGATCGCATGGTCCAGTACCGGCGACGCATCTTTCA   240
C. eriosoma rDNA   ............................................................   240
C. chalcites rDNA  ............................................................   240
C. includens rDNA  ............................................................   240

Consensus          AATGTCTGCCTTATCAACTTTCGATGGTAGTTTCTGCGACTACCATGGTTGTCACGGGTA   300
C. eriosoma rDNA   ............................................................   300
C. chalcites rDNA  ............................................................   300
C. includens rDNA  ............................................................   300

Consensus          ACGGGGAATCAGGGTTCGATTCCGGAGAGGGAGCCTGAGAAACGGCTACCACATCCAAGG   360
C. eriosoma rDNA   ............................................................   360
C. chalcites rDNA  ............................................................   360
C. includens rDNA  ............................................................   360

Consensus          AAGGCAGCAGGCGCGCAAATTACCCACTCCCGGCACGGGGAGGTAGTGACGAAAAATAAC   420
C. eriosoma rDNA   ............................................................   420
C. chalcites rDNA  ............................................................   420
C. includens rDNA  ............................................................   420

Consensus          GATACGGGACTCTTACGAGGCCTCGTAATCGGAATGAGTACACTTTAAATATTTTAACGA   480
C. eriosoma rDNA   ............................................................   480
C. chalcites rDNA  ............................................................   480
C. includens rDNA  ............................................................   480

Consensus          GGAACAATTGGAGGGCAAGTCTGGTGCCAGCAGCCGCGGTAATTCCAGCTCCAATAGCGT   540
C. eriosoma rDNA   ............................................................   540
C. chalcites rDNA  ............................................................   540
C. includens rDNA  ............................................................   540

Consensus          ATACTAAAATTGTTGCGGTTAAAAAGCTCGTAGTTGCATTTGTGCGCCGCGCTGTCGGTG   600
C. eriosoma rDNA   ............................................................   600
C. chalcites rDNA  ............................................................   600
C. includens rDNA  ............................................................   600

Consensus          CACCGCATCCGCGGTGATACTGACACGTCTGCGGAGCATATCGTCGGTGAGCCGGCGGTA   660
C. eriosoma rDNA   ............................................................   660
C. chalcites rDNA  ............................................................   660
C. includens rDNA  ............................................................   660

Consensus          ATACGCCGGTTCAATATCAAAATCCTATCGCGGTGCTCTTCGGTGAGTGTCGAGGTGGGC   720
```

```
C. eriosoma rDNA  ............................................................   720
C. chalcites rDNA ............................................................   720
C. includens rDNA ............................................................   720

Consensus         CGACAATTTTACTTTGAACAAATTAGAGTGCTCAAAGCGGGCTCAAAATGCTGCTTGAAT   780
C. eriosoma rDNA  ............................................................   780
C. chalcites rDNA ............................................................   780
C. includens rDNA ............................................................   780

Consensus         ATTTCGTGCATGGAATAATAGAATATGATCTCGGTTCTATTTTGTTGGTTTTCAGAACTC   840
C. eriosoma rDNA  ............................................................   840
C. chalcites rDNA ............................................................   840
C. includens rDNA ............................................................   840

Consensus         CGAGGTAATGATTAATAGGGATAACTGGGGGCATTCGTATTGCGACGTTAGAGGTGAAAT   900
C. eriosoma rDNA  ............................................................   900
C. chalcites rDNA ............................................................   900
C. includens rDNA ............................................................   900

Consensus         TCTTGGATCGTCGCAAGACGAACATCAGCGAAAGCATTTGCCAAAGGTGTTTTCATCAAT   960
C. eriosoma rDNA  ............................................................   960
C. chalcites rDNA ............................................................   960
C. includens rDNA ............................................................   960

Consensus         CAAGAACGAAAGTTAGAGGTTCGAAGGCGATTAGATACCGCCCTAGTTCTAACCGTAAAT  1020
C. eriosoma rDNA  ............................................................  1020
C. chalcites rDNA ............................................................  1020
C. includens rDNA ............................................................  1020

Consensus         ATGTCATCTAGCGATCCGCCGACGTTACTACAATGGCTCGGCGGGCAGCTTCCGGGAAAC  1080
C. eriosoma rDNA  ............................................................  1080
C. chalcites rDNA ............................................................  1080
C. includens rDNA ............................................................  1080

Consensus         CAAAGATTTTGGACTCCGGGGGAGTATGGTTGCAAAGCTGAAACTTAAAGGAATTGACGG  1140
C. eriosoma rDNA  ............................................................  1140
C. chalcites rDNA ............................................................  1140
C. includens rDNA ............................................................  1140

Consensus         AAGGGCACCACCAGGAGTGGAGCCTGCGGCTTAATTTGACTCAACACGGGAAATCTCACC  1200
C. eriosoma rDNA  ............................................................  1200
C. chalcites rDNA ............................................................  1200
C. includens rDNA ............................................................  1200

Consensus         AGGCCCGGACACCGGAAGGATTGACAGATTAACAGCTCTTTCTTGATTCGGTGGGTGGTG  1260
C. eriosoma rDNA  ............................................................  1260
C. chalcites rDNA ............................................................  1260
C. includens rDNA ............................................................  1260

Consensus         GTGCATGGCCGTTCTTAGTTGGTGGAGCGATTTGTCTGGTTAATTCCGGTAACGAACGAG  1320
C. eriosoma rDNA  ............................................................  1320
C. chalcites rDNA ............................................................  1320
C. includens rDNA ............................................................  1320

Consensus         ACTCTAGCCTGCTAAATAGGCGTCGTCATTTAGGTGTGCGTGGCTCCGTCACGCAACTCA  1380
C. eriosoma rDNA  ............................................................  1380
```

```
C. chalcites rDNA ............................................................  1380
C. includens rDNA ............................................................  1380

Consensus         CTGGCGACGTATTAAAATTCTTCTTAGAGGGACCGGCGGCTTCGAGCCGCACGAGATTGA  1440
C. eriosoma rDNA  ............................................................  1440
C. chalcites rDNA ............................................................  1440
C. includens rDNA ............................................................  1440

Consensus         GCAATAACAGGTCTGTGATGCCCTTAGATGTCCTGGGCCGCACGCGCGCTACACTGAAGG  1500
C. eriosoma rDNA  ............................................................  1500
C. chalcites rDNA ............................................................  1500
C. includens rDNA ............................................................  1500

Consensus         AATCAGCATGTTCTCCCTGGCCTAGAGGCCCGGGCAACCCGCTGAAACTCCTTCGTGCTG  1560
C. eriosoma rDNA  ............................................................  1560
C. chalcites rDNA ............................................................  1560
C. includens rDNA ............................................................  1560

Consensus         GGGATTGGGGTTTGCAATTATCCCCCATAAACGAGGAATTCCTAGTAAGCGCGAGTCATA  1620
C. eriosoma rDNA  ............................................................  1620
C. chalcites rDNA ............................................................  1620
C. includens rDNA ............................................................  1620

Consensus         AGCTCGCGTTGATTACGTCCCTGCCCTTTGTACACACCGCCCGTCGCTACTACCGATTGA  1680
C. eriosoma rDNA  ............................................................  1680
C. chalcites rDNA ............................................................  1680
C. includens rDNA ............................................................  1680

Consensus         ATGATTTAGTGAGGTCTTCGGACCGACACGCGGTGGCTTCACGGCCGTCGGCGTTGCTGG  1740
C. eriosoma rDNA  ............................................................  1740
C. chalcites rDNA ............................................................  1740
C. includens rDNA ............................................................  1740

Consensus         GAAGTTGACCAAACTTGATCATTTAGAGGAAGTAAAAGTCGTAACAAGGTTTCCGTAGGG  1800
C. eriosoma rDNA  ............................................................  1800
C. chalcites rDNA ............................................................  1800
C. includens rDNA ............................................................  1800

Consensus         GAACCTGCGGAAGGATCATTAACGTGTTACCGTTNNNNRCGYGCGCDMRCGCNNNCGSGA  1860
C. eriosoma rDNA  ..................................----G..C....ACA...---..C..  1853
C. chalcites rDNA ..................................----G..C....GAA...---..C..  1853
C. includens rDNA ..................................TTCAA..T....TCG...ATA..G..  1860

Consensus         AACGATGATACATTYATGTACYCCRAACAMAMTWWYRWATRWWAYASAKWCSRAYHMKRR  1920
C. eriosoma rDNA  ..............T......C..G....A.C.TTTAT..AAA.T.G.GA.CG.TACGGA  1913
C. chalcites rDNA ..............T......C..G....A.C.TTTAT..AAA.T.G.GA.CG.TACGGA  1913
C. includens rDNA ..............C......T..A....C.A.AACGA..GTT.C.C.TT.GA.CYATAG  1920

Consensus         ASRCGMGCGNNNNNNNNNNNNNNNNNNNNNNNNNNNNGCKSTSRTCGTKCGTCTCGCGTC  1980
C. eriosoma rDNA  .CG..A...TGTAAGAGCTTTAAACAGTTTTACGCGC..GC.GA....T...........  1973
C. chalcites rDNA .CG..A...TGTAAGAGCTTGAAACAGTTTTACGCGC..GC.GA....T...........  1973
C. includens rDNA .GA..C...----------------------------..TG.CG....G...........  1952

Consensus         GCGTAWGTTGATYWYRYRCTNNNNNNNNATACGGTTTAACATAAAATCCGCAAACCGTGA  2040
C. eriosoma rDNA  .....T......TTTGTA..--------................................  2025
C. chalcites rDNA .....T......TTTGTA..--------................................  2025
```

```
C. includens rDNA .....A......CACACG..TCGTTTTA................................  2012

Consensus         GWRGTRYNTTRTACGCGTTCGTTAANNNNYMCCGTCTCGTCGACGATTACGCGGTGTTCG  2100
C. eriosoma rDNA  .TG..AC-..G..............----TC.........................  2080
C. chalcites rDNA .TG..AC-..G..............----TC.........................  2080
C. includens rDNA .AA..GTA..A..............CATGCA.............................  2072

Consensus         TMGACGRGRNNNNNNNNNNNNNNNNTWWCTRTTRAAAAANWTWTWTWMANNNTHATATACT  2160
C. eriosoma rDNA  .A....A.ATAATTTATATATTTT.AA..A..A.....-A.A.A.AC.---.T.......  2136
C. chalcites rDNA .A....A.ATAATTTATATATTTT.AA..A..A.....TA.A.A.AC.---.A.......  2137
C. includens rDNA .C....G.G---------------.TT..G..G.....TT.T.T.TA.CAT.C.......  2117

Consensus         GTAACATGGGTTAATANNTWAAAACTATTACCCTGGACGGTGGATCACTTGGCTCGCGGG  2220
C. eriosoma rDNA  ................--.T........................................  2194
C. chalcites rDNA ................--.T........................................  2195
C. includens rDNA ................AT.A........................................  2177

Consensus         TCGATGAAGAACGCAGTTAACTGCGCGTCATAGTGTGAACTGCAGGACACATTTGAACAT  2280
C. eriosoma rDNA  ............................................................  2254
C. chalcites rDNA ............................................................  2255
C. includens rDNA ............................................................  2237

Consensus         CGACATTTCGAACGCACATTGCGGTCCGTGGAGACACATCCAGGACCACTCCTGTCTGAG  2340
C. eriosoma rDNA  ............................................................  2314
C. chalcites rDNA ............................................................  2315
C. includens rDNA ............................................................  2297

Consensus         GGCCGGCTGTATAAAGTAAACATGCCACATTGCGCASGTCTCGTYNNCGAGACKAGCGCA  2400
C. eriosoma rDNA  ....................................G.......CTA......T......  2374
C. chalcites rDNA ....................................G.......CTG......G......  2375
C. includens rDNA ....................................C.......T--......G......  2355

Consensus         TWTGACGGTGNYTCCGTCGRTYCGTCWCGTWCGANTRVGYGSKYSGCGTTCGCGCGYCGC  2460
C. eriosoma rDNA  .T........TT.......A.C....T...A...-.AA.C.GGCG...........C...  2433
C. chalcites rDNA .T........TT.......A.C....T...A...-.AC.C.GGCG...........C...  2434
C. includens rDNA .A........-C.......G.T....A...T...T.GG.T.CTTC...........T...  2414

Consensus         YCTCRANNTCKTGHGYGACGGATMWWTCGACGGTCCGTTCAAAAATAACGCTCTATACGA  2520
C. eriosoma rDNA  C...A.CG..G..A.T.......CAA..................................  2493
C. chalcites rDNA C...A.CG..G..A.T.......CAA..................................  2494
C. includens rDNA T...G.--..T..Y.C.......ATT..................................  2472

Consensus         TWGATAGGACATTTGTCGAACGRCTTGTRRAWCGCNNNNGCGTTRWKAAYNNNNTAAAAM  2580
C. eriosoma rDNA  .T....................A.....AA.T...TAAT.....GTT..T----.....C  2549
C. chalcites rDNA .T....................A.....AA.T...TAGT.....GTT..T----.....C  2550
C. includens rDNA .A....................G.....GG.A...----.....AAG..CACCG.....A  2528

Consensus         RWKAWCRWMKAYRCGYTTNNNNMMTMGACGGACGTACAAWGATCGCGAGKATGCGTWTAA  2640
C. eriosoma rDNA  AAT.A.GTAT.TG..T..----AC.C.............A.........G......T...  2605
C. chalcites rDNA AAT.A.GTAT.TG..T..----AC.C.............A.........G......T...  2606
C. includens rDNA GTG.T..ACG.CA..C..TACACA.A.............T.........T......A...  2588

Consensus         YGCTYYYYNNRKYWAGAGAMGCGTTTGCGCGATCGCGACCCTTCGGTCTAAATAACCAGM  2700
C. eriosoma rDNA  T...TCTC--GTCA.....C.......................................A  2663
C. chalcites rDNA T...TCTC--GTCA.....A.......................................A  2664
C. includens rDNA C...CTCTCAAGTT.....C.......................................C  2648
```

```
Consensus          GRGGGTCGAGARCTCWAGGTCTGMRTRTATCGTATNTCGACATAATNNCGTGTCCGMTAN  2760
C. eriosoma rDNA   .A.........A...A.......AA.A........A..........AC........A..-  2722
C. chalcites rDNA  .A.........A...A.......AA.G........A..........AC........A..-  2723
C. includens rDNA  .G.........G...T.......CG.A........-..........--........C..T  2705

Consensus          ATCGTTARRGRYYKAGTAGGCGGACTCGACGTCCGAAGAGCGCATCGACGCCGTAGTCGT  2820
C. eriosoma rDNA   .......GA.ACTT..............................................  2782
C. chalcites rDNA  .......GA.ACTT..............................................  2783
C. includens rDNA  .......AG.GTCG..............................................  2765

Consensus          TCRTGCGAGTCTAGCCGCGTTCNNAACGCGTNWYTATTCTCGTGTCCGATTGCGTCGTCG  2880
C. eriosoma rDNA   ..A...................TG.......AAC..........................  2842
C. chalcites rDNA  ..A...................TG.......AAC..........................  2843
C. includens rDNA  ..G...................--.......-TT..........................  2822

Consensus          TAACGCTGACGGATATCGCGTCTGCCTCATTTTTTTATCGTTGGCCTCAGATCAGGGAGG  2940
C. eriosoma rDNA   ............................................................  2902
C. chalcites rDNA  ............................................................  2903
C. includens rDNA  ............................................................  2882

Consensus          ATCACCCGCCGAATTTAAGCATATTAGTAAGCGGAGGAAAAGAAACTAACCAGGATTTCC  3000
C. eriosoma rDNA   ............................................................  2962
C. chalcites rDNA  ............................................................  2963
C. includens rDNA  ............................................................  2942

Consensus          TTAGTAGCGGCGAGCGAACAGGAAAGAAGCCCAGCACTGAATCCCGCCGTTGTTCAGGCG  3060
C. eriosoma rDNA   ............................................................  3022
C. chalcites rDNA  ............................................................  3023
C. includens rDNA  ............................................................  3002

Consensus          GCGGGAGATGTGGTGTTCGGGAGGTTCCGCTTTCTCGTCGCGATCGCTCCTGTCCAAGTT  3120
C. eriosoma rDNA   ............................................................  3082
C. chalcites rDNA  ............................................................  3083
C. includens rDNA  ............................................................  3062

Consensus          CGTCTTGAACGGGGCCGTTTTCCCGTAGAGGGTGCCAGGCCCGTAGCGACGGAGGATTGC  3180
C. eriosoma rDNA   ............................................................  3142
C. chalcites rDNA  ............................................................  3143
C. includens rDNA  ............................................................  3122

Consensus          GGCGAGAGGGACTCTCCTTAGAGTCGGGTTGCTTGAGAGTGCAGCCCTAAGTGGGTGGTA  3240
C. eriosoma rDNA   ............................................................  3202
C. chalcites rDNA  ............................................................  3203
C. includens rDNA  ............................................................  3182

Consensus          AACTCCATCTAAGGCTAAATATTACCGCGAGACCGATAGCGAACAAGTACCGTGAGGGAA  3300
C. eriosoma rDNA   ............................................................  3262
C. chalcites rDNA  ............................................................  3263
C. includens rDNA  ............................................................  3242

Consensus          AGTTGAAAAGAACTTTGAAGAGAGAGTTCAAGAGTACGTGAAACCGTTCAGGGGTAAACC  3360
C. eriosoma rDNA   ............................................................  3322
C. chalcites rDNA  ............................................................  3323
C. includens rDNA  ............................................................  3302
```

```
Consensus          TGCGAAACTCGAATGAACGAACGGAGAGATTCATCGTCATTCCYCGGCGTACGGACGCGC  3420
C. eriosoma rDNA   ...........................................C................  3382
C. chalcites rDNA  ...........................................C................  3383
C. includens rDNA  ...........................................T................  3362

Consensus          GCCTCGATGTCGCCGATCTCGATCGGCYGGCACGGGTCGCGTCCGTCGACGTCCGAGGAC  3480
C. eriosoma rDNA   ...........................T................................  3442
C. chalcites rDNA  ...........................T................................  3443
C. includens rDNA  ...........................C................................  3422

Consensus          GGCGYGCACTTCTCTCTTAGTAAATACATCGCGACCCGTTCGATGTCGGTCTAAGCGCCG  3540
C. eriosoma rDNA   ....T.......................................................  3502
C. chalcites rDNA  ....T.......................................................  3503
C. includens rDNA  ....C.......................................................  3482

Consensus          TACGGGAGCCCCGTTGCCCCTTCACGGGGGTAGTGGGACCGCGACGGTGGCCGACCGGCC  3600
C. eriosoma rDNA   ............................................................  3562
C. chalcites rDNA  ............................................................  3563
C. includens rDNA  ............................................................  3542

Consensus          GTCGGACGGTAGTTCTGACGAATCGCGCACGCGTTTTAGACGCGTCCGGCCCGACGCAAG  3660
C. eriosoma rDNA   ............................................................  3622
C. chalcites rDNA  ............................................................  3623
C. includens rDNA  ............................................................  3602

Consensus          TCAACGTCGTATCCAACGTCTACGCCACAGTGCGGACGTAGGTGCGGCGCGTCTGYMGTC  3720
C. eriosoma rDNA   .......................................................TC...  3682
C. chalcites rDNA  .......................................................TC...  3683
C. includens rDNA  .......................................................CA...  3662

Consensus          GCMGCCGTGTTGTCACGGACTGTGCGCGTCTCTGTCTGCGATGATTCAGTTTCGGGCACT  3780
C. eriosoma rDNA   ..A.........................................................  3742
C. chalcites rDNA  ..A.........................................................  3743
C. includens rDNA  ..C.........................................................  3722

Consensus          CGCAGGACCCGTCTTGAAACACGGACCAAGGAGTCTAGCATGTATGCGAGTCATTGAGAT  3840
C. eriosoma rDNA   ............................................................  3802
C. chalcites rDNA  ............................................................  3803
C. includens rDNA  ............................................................  3782

Consensus          AATAAAACTGAAAGGCGCAACGAAAGTGAAGGCGCGCGCTAGCCGCGTGCTCAGGGAGGA  3900
C. eriosoma rDNA   ............................................................  3862
C. chalcites rDNA  ............................................................  3863
C. includens rDNA  ............................................................  3842

Consensus          TGGAGCGTCGATCTAGGTCGATCTCTCGCACTCCCGAGGCGTCTCGTTTCCAATCCGTGA  3960
C. eriosoma rDNA   ............................................................  3922
C. chalcites rDNA  ............................................................  3923
C. includens rDNA  ............................................................  3902

Consensus          ATGCAGGCGCGCTCTGAGCATAAATGCTGGGACCCGAAAGATGGTGAACTATGCCTGGTC  4020
C. eriosoma rDNA   ............................................................  3982
C. chalcites rDNA  ............................................................  3983
C. includens rDNA  ............................................................  3962

Consensus          AGGTCGAAGTCAGGGGAAACCCTGATGGAGGACCGTAGCGATTCTGACGTGCAAATCGAT  4080
```

```
C. eriosoma rDNA  ............................................................  4042
C. chalcites rDNA ............................................................  4043
C. includens rDNA ............................................................  4022

Consensus         CGTCGGAACTGGGTATAGGGGCGAAAGACTAATCGAACCATCTAGTAGCTGGTTCCGTCC  4140
C. eriosoma rDNA  ............................................................  4102
C. chalcites rDNA ............................................................  4103
C. includens rDNA ............................................................  4082

Consensus         GAAGTTTCCCTCAGGATAGCTGGCGTCGATTTGAACAGTCTCATCCGGTAAAGCGAATGA  4200
C. eriosoma rDNA  ............................................................  4162
C. chalcites rDNA ............................................................  4163
C. includens rDNA ............................................................  4142

Consensus         TTAGAGGCATTGGGGCCGAAACGACCTCAACCTATTCTCAAACTTTAAATGGGTGAGTAC  4260
C. eriosoma rDNA  ............................................................  4222
C. chalcites rDNA ............................................................  4223
C. includens rDNA ............................................................  4202

Consensus         TCCGGCTTACTCGAACGATGAAGCCGGAGATCTGATGACGGTGCCAAGTGGGCCAATTTT  4320
C. eriosoma rDNA  ............................................................  4282
C. chalcites rDNA ............................................................  4283
C. includens rDNA ............................................................  4262

Consensus         GGTAAGCAGAACTGGCGCTGTGGGATGAACCAAACGTAGTGTTAAGGCGCCTAAAAAACG  4380
C. eriosoma rDNA  ............................................................  4342
C. chalcites rDNA ............................................................  4343
C. includens rDNA ............................................................  4322

Consensus         CTCATGGGACACCATGAAAGGCGTTGGTCGCTCATGACAGCAGGACGGTGGCCATGGAAG  4440
C. eriosoma rDNA  ............................................................  4402
C. chalcites rDNA ............................................................  4403
C. includens rDNA ............................................................  4382

Consensus         TCGGAATCCGCTAAGGAGTGTGCAACGACTCACCTGCCGAAGCAACCAGCCCTGAAAATG  4500
C. eriosoma rDNA  ............................................................  4462
C. chalcites rDNA ............................................................  4463
C. includens rDNA ............................................................  4442

Consensus         GATGGCGCTGAAGCGTTTTGCCTATACACTACCGTTACGGGCATGTGCGACGTTCTTTGY  4560
C. eriosoma rDNA  ...........................................................T  4522
C. chalcites rDNA ...........................................................T  4523
C. includens rDNA ............................................................  4502

Consensus         GACGTCATTAAGCCGTAACGAGTAGGACGTGCGCGGCGGAGAGCGCAGAAGGGTCTGGGC  4620
C. eriosoma rDNA  ............................................................  4582
C. chalcites rDNA ............................................................  4583
C. includens rDNA ............................................................  4562

Consensus         GTGAGCCCGCTTGGAGCCTCCGTCGGTGCAGATCTTGGTGGTAGTAGCAAATACTCCAGC  4680
C. eriosoma rDNA  ............................................................  4642
C. chalcites rDNA ............................................................  4643
C. includens rDNA ............................................................  4622

Consensus         GAGGCCCTGGAGGACTGACGTGGAGAAGGGTTTCGCGTGAACAGTAGTTGCTCGCGAGTC  4740
C. eriosoma rDNA  ............................................................  4702
```

```
C. chalcites rDNA ............................................................  4703
C. includens rDNA ............................................................  4682

Consensus         AGTCGATCCTAAGCTCAAGGAGAAATCTTATGTCGATGTGGCGTGTTTTTTTCAATRATG  4800
C. eriosoma rDNA  ........................................................A...  4762
C. chalcites rDNA ........................................................A...  4763
C. includens rDNA ........................................................G...  4742

Consensus         TATCRTTTTATWATGGTATATAATGATAAATAACGCCCTTTGAGCGAAAGGGAATCCGGT  4860
C. eriosoma rDNA  ....A......T................................................  4822
C. chalcites rDNA ....G.......................................................  4823
C. includens rDNA ....G......T................................................  4802

Consensus         TCCTATTCCGGAACCCGGCAGCGGAACCGTTTCAATAATCGTTCCCTCGTTTTTACAGCG  4920
C. eriosoma rDNA  ............................................................  4882
C. chalcites rDNA ............................................................  4883
C. includens rDNA ............................................................  4862

Consensus         AGTGTTCGACGGGGTAACCCAAAGTGGCCTGAAGACGCCGCCGAGAGGTCCGGGAAGAGT  4980
C. eriosoma rDNA  ............................................................  4942
C. chalcites rDNA ............................................................  4943
C. includens rDNA ............................................................  4922

Consensus         TTTCTTTTCTGCCTGAGCGTTCGAGTTCCATGGAATCCTATAGAAGGGAGATATGGTTCG  5040
C. eriosoma rDNA  ............................................................  5002
C. chalcites rDNA ............................................................  5003
C. includens rDNA ............................................................  4982

Consensus         GAACGCGAAGAGCACCGCATTTGCGGCGGTGTCCGGATACTCTCTGCGGACCTTGAAAAT  5100
C. eriosoma rDNA  ............................................................  5062
C. chalcites rDNA ............................................................  5063
C. includens rDNA ............................................................  5042

Consensus         TCAGGTGAGGGATGTACGTGGAGATGTCGCGCCGGTTCGTACCCATATCCGCAGCAGGTC  5160
C. eriosoma rDNA  ............................................................  5122
C. chalcites rDNA ............................................................  5123
C. includens rDNA ............................................................  5102

Consensus         TCCAAGGTGAAGAGCCTCTAGTCGATAGAATAATGTAGGTAAGGGAAGTCGGCAAATTGG  5220
C. eriosoma rDNA  ............................................................  5182
C. chalcites rDNA ............................................................  5183
C. includens rDNA ............................................................  5162

Consensus         ATCCGTAACTTCGGAATAAGGATTGGCTCTGAGGACCGGGGCGTGTCGGGTTTGGACGGG  5280
C. eriosoma rDNA  ............................................................  5242
C. chalcites rDNA ............................................................  5243
C. includens rDNA ............................................................  5222

Consensus         AAGCGGATGCGGCCGGTGCCGGGCCTGGTCGATGCTCTMGCGTCTTTCGGGGCGCGAGGG  5340
C. eriosoma rDNA  ......................................C.....................  5302
C. chalcites rDNA ......................................C.....................  5303
C. includens rDNA ......................................A.....................  5282

Consensus         CGGAATCCGGACCCGCGTTCCGGCCTTCCGCGGATCTTCCTAGCCGTAAGGCCGTGTCKG  5400
C. eriosoma rDNA  ..........................................................G.  5362
C. chalcites rDNA ..........................................................G.  5363
```

```
C. includens rDNA ..........................................................T.  5342

Consensus         TCTCGACTCGTGCGCGATCGGCGCGGTTCTGTACGACCGCCGTTCAACGGTCAGCTCAGA  5460
C. eriosoma rDNA  ............................................................  5422
C. chalcites rDNA ............................................................  5423
C. includens rDNA ............................................................  5402

Consensus         ACTGGCACGGACAAGGGGAATCCGACTGTCTAATTAAAACAAAGCATTGCGATGGCCCTC  5520
C. eriosoma rDNA  ............................................................  5482
C. chalcites rDNA ............................................................  5483
C. includens rDNA ............................................................  5462

Consensus         GCGGGTGTTGACGCAATGTGATTTCTGCCCAGTGCTCTGAATGTCAACGTGAAGAAATTC  5580
C. eriosoma rDNA  ............................................................  5542
C. chalcites rDNA ............................................................  5543
C. includens rDNA ............................................................  5522

Consensus         AAGCAAGCGCGGGTAAACGGCGGGAGTAACTATGACTCTCTTAAGGTAGCCAAATGCCTC  5640
C. eriosoma rDNA  ............................................................  5602
C. chalcites rDNA ............................................................  5603
C. includens rDNA ............................................................  5582

Consensus         GTCATCTAATTAGTGACGCGCATGAATGGATTAACGAGATTCCCACTGTCCCTATCTACT  5700
C. eriosoma rDNA  ............................................................  5662
C. chalcites rDNA ............................................................  5663
C. includens rDNA ............................................................  5642

Consensus         ATCTAGCGAAACCACAGCCAAGGGAACGGGCTTGGGAGAATCAGCGGGGAAAGAAGACCC  5760
C. eriosoma rDNA  ............................................................  5722
C. chalcites rDNA ............................................................  5723
C. includens rDNA ............................................................  5702

Consensus         TGTTGAGCTTGACTCTAGTCTGGCATTGTAAGGAGACATGAGAGGTGTAGCATAAGTGGG  5820
C. eriosoma rDNA  ............................................................  5782
C. chalcites rDNA ............................................................  5783
C. includens rDNA ............................................................  5762

Consensus         AGATCGTTCGCGCGATCGTCGCTGAAAAACCACTACTTTCATTGTTTCATTACTTACTCG  5880
C. eriosoma rDNA  ............................................................  5842
C. chalcites rDNA ............................................................  5843
C. includens rDNA ............................................................  5822

Consensus         GTTGGGCGGAAGCGGTGCGCGGTCGATATYAATCGGCGGGCGCACGGTGTTTCGTTCCAA  5940
C. eriosoma rDNA  .............................T..............................  5902
C. chalcites rDNA .............................T..............................  5903
C. includens rDNA .............................C..............................  5882

Consensus         GCGTGCAGAGTGGCGACGTGGCGGCAACGCTCGTCGCCGTAAAACTCCCGCGTGATCCGG  6000
C. eriosoma rDNA  ............................................................  5962
C. chalcites rDNA ............................................................  5963
C. includens rDNA ............................................................  5942

Consensus         TTCGAGGACACTGCCAGGCGGGTAGTTTGACTGGGGCGGTACATCTGTCAAAGAATAACG  6060
C. eriosoma rDNA  ............................................................  6022
C. chalcites rDNA ............................................................  6023
C. includens rDNA ............................................................  6002
```

```
Consensus          CAGGTGTCCTAAGGCCAGCTCAGCGAGGACAGAAACCTCGCGTGGAGCAAAAGGGCAAAA  6120
C. eriosoma rDNA   ............................................................  6082
C. chalcites rDNA  ............................................................  6083
C. includens rDNA  ............................................................  6062

Consensus          GCTGGCTTGATCCAGATGTTCAGTACGCATAGGGACTGCGAAAGCACGGCCTATCGATCC  6180
C. eriosoma rDNA   ............................................................  6142
C. chalcites rDNA  ............................................................  6143
C. includens rDNA  ............................................................  6122

Consensus          TTTAGTATAAAGAGTTTTTAGCAAGAGGTGCCAGAAAAGTTACCACAGGGATAACTGGCT  6240
C. eriosoma rDNA   ............................................................  6202
C. chalcites rDNA  ............................................................  6203
C. includens rDNA  ............................................................  6182

Consensus          TGTGGCAGCCAAGCGTTCATAGCGACGTTGCTTTTTGATCCTTCGATGTCGGCTCTTCCT  6300
C. eriosoma rDNA   ............................................................  6262
C. chalcites rDNA  ............................................................  6263
C. includens rDNA  ............................................................  6242

Consensus          ATCATTGCGAAGCAAAATTCGCCAAGCGTTGGATTGTTCACCCATCAAAAGGGAACGTGA  6360
C. eriosoma rDNA   ............................................................  6322
C. chalcites rDNA  ............................................................  6323
C. includens rDNA  ............................................................  6302

Consensus          GCTGGGTTTAGACCGTCGTGAGACAGGTTAGTTTTACCCTACTGATGGCTTGTCGTTGCG  6420
C. eriosoma rDNA   ............................................................  6382
C. chalcites rDNA  ............................................................  6383
C. includens rDNA  ............................................................  6362

Consensus          ATAGTAATACTGCTCAGTACGAGAGGAACCGCAGTTTCGGACATTTGGTTCATGCACTCG  6480
C. eriosoma rDNA   ............................................................  6442
C. chalcites rDNA  ............................................................  6443
C. includens rDNA  ............................................................  6422

Consensus          GCCGAGCGGCCGGTGGTGCGAAGCTACCATCCGCGGGATTATGCCTGAACG           6531
C. eriosoma rDNA   ...................................................           6493
C. chalcites rDNA  ...................................................           6494
C. includens rDNA  ...................................................           6473
```

C. includens diagnostic primer loci

C. includens diagnostic probe locus

C. chalcites/ C. eriosoma diagnostic primer loci

C. chalcites/ C. eriosoma diagnostic probe locus
