# Supplementary material for: Ultra-deep sequencing of 45S rDNA to discern intragenomic diversity in three Chrysodeixis species for molecular identification
Source: Sci Rep. 2023 Aug 10;13:13017. doi: 10.1038/s41598-023-39673-7 (PMC10415407; doi:10.1038/s41598-023-39673-7)
Supplement: Supplementary file 3 — Supplementary Information 3. [file 41598_2023_39673_MOESM3_ESM.pdf]

```
Consensus               TAGCTCCCTGGTTGATCCTGCCAGTAGTTATATGCTTGTCTCAAAGATTAAGCCATGCAT   60
Papilio xuthus          ............................................................   60
Pro. anacardii duprei   ............................................................   60
Kallimoides rumia       ............................................................   60
Araschina levana        ............................................................   60
Junonia stygia          ............................................................   60
A. jatrophae saturate   ............................................................   60
Mallika jacksoni        ............................................................   60
Helicoverpa zea         A...........................................................   60
Helicoverpa armigera    A...........................................................   60
Chrysodeixis includens  ------------------------------------------------------------
Chrysodeixis eriosoma   ------------------------------------------------------------
Chrysodeixis chalcites  ------------------------------------------------------------
Coeliades ramanatek     C...........................................................   60
Meroptera pravella      ............................................................   60
Salamis anteva          ............................................................   60
Precis andremiaja       ............................................................   60

Consensus               GTCTCAGTGCAAGCCGTATTAAGGCGATACCGCGAATGGCTCAATATATCAGTTTTGGTT  120
Papilio xuthus          ............................................................  120
Pro. anacardii duprei   ............................................................  120
Kallimoides rumia       ............................................................  120
Araschina levana        ............................................................  120
Junonia stygia          ............................................................  120
A. jatrophae saturata   ............................................................  120
Mallika jacksoni        ............................................................  120
Helicoverpa zea         ............................................................  120
Helicoverpa armigera    ............................................................  120
Chrysodeixis includens  ------------------------....................................   36
Chrysodeixis eriosoma   ------------------------....................................   36
Chrysodeixis chalcites  ------------------------....................................   36
Coeliades ramanatek     ............................................................  120
Meroptera pravella      ............................................................  120
Salamis anteva          ............................................................  120
Precis andremiaja       ............................................................  120

Consensus               CCTTAGATCTTAC-TCAGTTACTTGGATAACTGTGGTAATTCTAGAGCTAATACATGCAA  179
Papilio xuthus          .............C..G...........................................  180
Pro. anacardii duprei   .............-..............................................  179
Kallimoides rumia       .............-..............................................  179
Araschina levana        .............-..............................................  179
Junonia stygia          .............-..............................................  179
A. jatrophae saturata   ....R........-..............................................  179
Mallika jacksoni        .............-..............................................  179
Helicoverpa zea         .............-..............................................  179
Helicoverpa armigera    .............-..............................................  179
Chyrsodeixis includens  .............-..............................................   95
Chrysodeixis eriosoma   .............-..............................................   95
Chrysodeixis chalcites  .............-..............................................   95
Coeliades ramanatek     .............-..............................................  179
Meroptera pravella      .............-..............................................  179
Salamis anteva          .............-..............................................  179
Precis andremiaja       .............-..............................................  179

Consensus               TCAGAACTCTGACCAGTGATGGGATGAGTGCTTTTATTAGATCAAAACCAATCGACGGAG  239
Papilio xuthus          ............................................................  240
```

```
Pro. anacardii duprei     .A..........................................................   239
Kallimoides rumia         .A..........................................................   239
Araschina levana          ............................................................   239
Junonia stygia            .A..........................................................   239
A. jatrophae saturata     .A..........................................................   239
Mallika jacksoni          ............................................................   239
Helicoverpa zea           ............................................................   239
Helicoverpa armigera      ............................................................   239
Chrysodeixis includens    ............................................................   155
Chrysodeixis eriosoma     ............................................................   155
Chrysodeixis chalcites    ............................................................   155
Coeliades ramanatek       ............................................................   239
Meroptera pravella        ............................................................   239
Salamis anteva            .A..........................................................   239
Precis andremiaja         .A..........................................................   239

Consensus                 GGCCTCGCGTCCGAAGTCGTTAATTTTGATGAATCTGGATAACTTTTGCCGATCGCATGG   299
Papilio xuthus            ............................................................   300
Pro. anacardii duprei     ...G.TT.....................................................   299
Kallimoides rumia         ...G..T..........................................A..........   299
Araschina levana          ...A..C.....................................................   299
Junonia stygia            ...G..T....T................................................   299
A. jatrophae saturata     ...G..T.....................................................   299
Mallika jacksoni          ...G.TT.....................................................   299
Helicoverpa zea           ............................................................   299
Helicoverpa armigera      ............................................................   299
Chrysodeixis includens    ....A......T................................................   215
Chrysodeixis eriosoma     ....A.......T...............................................   215
Chrysodeixis chalcites    ....A.......T...............................................   215
Coeliades ramanatek       ............................................................   299
Meroptera pravella        ............................................................   299
Salamis anteva            ...G.TT.....................................................   299
Precis andremiaja         ...GC.T.....................................................   299

Consensus                 TCCAGTACCGGCGACGCATCTTTCAAATGTCTGCCTTATCAACTTTCGATGGTAGTTTCT   359
Papilio xuthus            .....C......................................................   360
Pro. anacardii duprei     ............................................................   359
Kallimoides rumia         ............................................................   359
Araschina levana          ............................................................   359
Junonia stygia            ............................................................   359
A. jatrophae saturata     ............................................................   359
Mallika jacksoni          ............................................................   359
Helicoverpa zea           ............................................................   359
Helicoverpa armigera      ............................................................   359
Chrysodeixis includens    ............................................................   275
Chrysodeixis eriosoma     ............................................................   275
Chrysodeixis chalcites    ............................................................   275
Coeliades ramanatek       ............................................................   359
Meroptera pravella        ............................................................   359
Salamis anteva            ............................................................   359
Precis andremiaja         ............................................................   359

Consensus                 GCGACTACCATGGTTGTCACGGGTAACGGGGAATCAGGGTTCGATTCCGGAGAGGGAGCC   419
Papilio xuthus            ............................................................   420
Pro. anacardii duprei     ............................................................   419
Kallimoides rumia         ............................................................   419
```

```
Araschina levana          ............................................................   419
Junonia stygia            ............................................................   419
A. jatrophae saturate     ............................................................   419
Mallika jacksoni          ............................................................   419
Helicoverpa zea           ............................................................   419
Helicoverpa armigera      ............................................................   419
Chrysodeixis includens    ............................................................   335
Chyrsodeixis eriosoma     ............................................................   335
Chrysodeixis chalcites    ............................................................   335
Coeliades ramanatek       ............................................................   419
Meroptera pravella        ............................................................   419
Salamis anteva            ............................................................   419
Precis andremiaja         ............................................................   419

Consensus                 TGAGAAACGGCTACCACATCCAAGGAAGGCAGCAGGCGCGCAAATTACCCACTCCCGGCA   479
Papilio xuthus            ............................................................   480
Pro. anacardii duprei     ............................................................   479
Kallimoides rumia         ............................................................   479
Araschina levana          ............................................................   479
Junonia stygia            ............................................................   479
A. jatrophae saturata     ............................................................   479
Mallika jacksoni          ............................................................   479
Helicoverpa zea           ............................................................   479
Helicoverpa armigera      ............................................................   479
Chrysodeixis includens    ............................................................   395
Chrysodeixis eriosoma     ............................................................   395
Chrysodeixis chalcites    ............................................................   395
Coeliades ramanatek       ............................................................   479
Meroptera pravella        ............................................................   479
Salamis anteva            ............................................................   479
Precis andremiaja         ............................................................   479

Consensus                 CGGGGAGGTAGTGACGAAAAATAACGATACGGGACTCTTWCGAGGCCTCGTAATCGGAAT   539
Papilio xuthus            .......................................A....................   540
Pro. anacardii duprei     .......................................T....................   539
Kallimoides rumia         .......................................T....................   539
Araschina levana          .......................................T....................   539
Junonia stygia            .......................................T....................   539
A. jatrophae saturata     .......................................T....................   539
Mallika jacksoni          .......................................T....................   539
Helicoverpa zea           .......................................A....................   539
Helicoverpa armigera      .......................................A....................   539
Chrysodeixis includens    .......................................A....................   455
Chrysodeixis eriosoma     .......................................A....................   455
Chrysodeixis chalcites    .......................................A....................   455
Coeliades ramanatek       .......................................A....................   539
Meroptera pravella        .......................................A....................   539
Salamis anteva            .......................................T....................   539
Precis andremiaja         .......................................T....................   539

Consensus                 GAGTACACTTTAAATATTTTAACGAGGAACAATTGGAGGGCAAGTCTGGTGCCAGCAGCC   599
Papilio xuthus            ............................................................   600
Pro. anacardii duprei     ............................................................   599
Kallimoides rumia         ............................................................   599
Araschina levana          ............................................................   599
Junonia stygia            ............................................................   599
```

```
A. jatrophae saturata  ............................................................   599
Mallika jacksoni       ............................................................   599
Helicoverpa zea        ............................................................   599
Helicoverpa armigera   ............................................................   599
Chrysodeixis includens ............................................................   515
Chrysodeixis eriosoma  ............................................................   515
Chrysodeixis chalcites ............................................................   515
Coeliades ramanatek    ............................................................   599
Meroptera pravella     ............................................................   599
Salamis anteva         ............................................................   599
Precis andremiaja      ............................................................   599

Consensus              GCGGTAATTCCAGCTCCAATAGCGTATACTAAAATTGTTGCGGTTAAAAAGCTCGTAGTT   659
Papilio xuthus         .......C....................................................   660
Pro. anacardii duprei  ............................................................   659
Kallimoides rumia      ............................................................   659
Araschina levana       ............................................................   659
Junonia stygia         ............................................................   659
A. jatrophae saturata  ............................................................   659
Mallika jacksoni       ............................................................   659
Helicoverpa zea        ............................................................   659
Helicoverpa armigera   ............................................................   659
Chrysodeixis includens ............................................................   575
Chrysodeixis eriosoma  ............................................................   575
Chrysodeixis chalcites ............................................................   575
Coeliades ramanatek    ............................................................   659
Meroptera pravella     ............................................................   659
Salamis anteva         ............................................................   659
Precis andremiaja      ............................................................   659

Consensus              GCATTTGTGCGCCGCGCTGTCGGTGCACCGCATCCGCGGTGATACTGACACGTCTGCGGA   719
Papilio xuthus         ............................................................   720
Pro. anacardii duprei  ............................................................   719
Kallimoides rumia      ............................................................   719
Araschina levana       ............................................................   719
Junonia stygia         ............................................................   719
A. jatrophae saturata  ............................................................   719
Mallika jacksoni       ............................................................   719
Helicoverpa zea        ............................................................   719
Helicoverpa armigera   ............................................................   719
Chrysodeixis includens ............................................................   635
Chrysodeixis eriosoma  ............................................................   635
Chrysodeixis chalcites ............................................................   635
Coeliades ramanatek    ............................................................   719
Meroptera pravella     .....................................................T......   719
Salamis anteva         ............................................................   719
Precis andremiaja      ............................................................   719

Consensus              GCATATCGTCGGTGAGCCGGCGGTAAAACGCCGGTTCAATATCAAAATCCTATCGCGGTG   779
Papilio xuthus         ...................T......T...G.............................   780
Pro. anacardii duprei  ............................................................   779
Kallimoides rumia      ............................................................   779
Araschina levana       ............................................................   779
Junonia stygia         ............................................................   779
A. jatrophae saturata  ............................................................   779
Mallika jacksoni       ............................................................   779
```

```
Helicoverpa zea         ..........................T.................................   779
Helicoverpa armigera    ..........................T.................................   779
Chrysodeixis includens  ..........................T.................................   695
Chrysodeixis eriosoma   ..........................T.................................   695
Chrysodeixis chalcites  ..........................T.................................   695
Coeliades ramanatek     ....................TT......T...............................   779
Meroptera pravella      ............................................................   779
Salamis anteva          ............................................................   779
Precis andremiaja       ............................................................   779

Consensus               CTCTTCGGTGAGTGTCGAGATGGGCCGACAATTTTACTTTGAACAAATTAGAGTGCTCAA   839
Papilio xuthus          ............................................................   840
Pro. anacardii duprei   ............................................................   839
Kallimoides rumia       ............................................................   839
Araschina levana        ............................................................   839
Junonia stygia          ............................................................   839
A. jatrophae saturata   ............................................................   839
Mallika jacksoni        ............................................................   839
Helicoverpa zea         ...................G........................................   839
Helicoverpa armigera    ...................G........................................   839
Chrysodeixis includens  ...................G........................................   755
Chyrsodeixis eriosoma   ...................G........................................   755
Chrysodeixis chalcites  ...................G........................................   755
Coeliades ramanatek     ......A.....................................................   839
Meroptera pravella      ......A............G........................................   839
Salamis anteva          ............................................................   839
Precis andremiaja       ............................................................   839

Consensus               AGCGGGCTCAAAATGCYGCTTGAATATTTCGTGCATGGAATAATAGAATATGATCTCGGT   899
Papilio xuthus          ..............TGT...........................................   900
Pro. anacardii duprei   ................C...........................................   899
Kallimoides rumia       .........T..G...C...........................................   899
Araschina levana        ................C...........................................   899
Junonia stygia          ................C...........................................   899
A. jatrophae saturata   ................C...........................................   899
Mallika jacksoni        ................C...........................................   899
Helicoverpa zea         ................T...........................................   899
Helicoverpa armigera    ................T...........................................   899
Chrysodeixis includens  ................T...........................................   815
Chrysodeixis eriosoma   ................T...........................................   815
Chrysodeixis chalcites  ................T...........................................   815
Coeliades ramanatek     ................T...........................................   899
Meroptera pravella      ................T...........................................   899
Salamis anteva          ................C...........................................   899
Precis andremiaja       ................C...........................................   899

Consensus               TCTATTTTGTTGGTTTTCAGAACTCCGAGGTAATGATTAATAGGGATAACTGGGGGCATT   959
Papilio xuthus          ............................................................   960
Pro. anacardii duprei   ............................................................   959
Kallimoides rumia       ............................................................   959
Araschina levana        ............................................................   959
Junonia stygia          ............................................................   959
A. jatrophae saturata   ............................................................   959
Mallika jacksoni        ............................................................   959
Helicoverpa zea         ............................................................   959
Helicoverpa armigera    ............................................................   959
```

```
Chrysodeixis includens ............................................................  875
Chrysodeixis eriosoma  ............................................................  875
Chrysodeixis chalcites ............................................................  875
Coeliades ramanatek    ............................................................  959
Meroptera pravella     ............................................................  959
Salamis anteva         ............................................................  959
Precis andremiaja      ............................................................  959

Consensus              CGTATTGCGACGTTAGAGGTGAAATTCTTGGATCGTCGCAAGACGAACATCAGCGAAAGC  1019
Papilio xuthus         ............................................................  1020
Pro. anacardii duprei  ............................................................  1019
Kallimoides rumia      ............................................................  1019
Araschina levana       ............................................................  1019
Junonia stygia         ............................................................  1019
A. jatrophae saturata  ............................................................  1019
Mallika jacksoni       ............................................................  1019
Helicoverpa zea        ............................................................  1019
Helicoverpa armigera   ............................................................  1019
Chrysodeixis includens ............................................................  935
Chrysodeixis eriosoma  ............................................................  935
Chrysodeixis chalcites ............................................................  935
Coeliades ramanatek    ............................................................  1019
Meroptera pravella     ............................................................  1019
Salamis anteva         ............................................................  1019
Precis andremiaja      ............................................................  1019

Consensus              ATTTGCCAAAGGTGTTTTCATCAATCAAGAACGAAAGTTAGAGGTTCGAAGGCGATTAGA  1079
Papilio xuthus         ............................................................  1080
Pro. anacardii duprei  ............................................................  1079
Kallimoides rumia      ............................................................  1079
Araschina levana       ............................................................  1079
Junonia stygia         ............................................................  1079
A. jatrophae saturata  ............................................................  1079
Mallika jacksoni       ............................................................  1079
Helicoverpa zea        ............................................................  1079
Helicoverpa armigera   ............................................................  1079
Chrysodeixis includens ............................................................  995
Chrysodeixis eriosoma  ............................................................  995
Chrysodeixis chalcites ............................................................  995
Coeliades ramanatek    ............................................................  1079
Meroptera pravella     ............................................................  1079
Salamis anteva         ............................................................  1079
Precis andremiaja      ............................................................  1079

Consensus              TACCGCCCTAGTTCTAACCGTAAATATGTCATCTAGCGATCCGCCGACGTTACTACAATG  1139
Papilio xuthus         ........................................................T...  1140
Pro. anacardii duprei  ............................................................  1139
Kallimoides rumia      ............................................................  1139
Araschina levana       ............................................................  1139
Junonia stygia         ............................................................  1139
A. jatrophae saturata  ............................................................  1139
Mallika jacksoni       ............................................................  1139
Helicoverpa zea        ............................................................  1139
Helicoverpa armigera   ............................................................  1139
Chrysodeixis includens ............................................................  1055
Chrysodeixis eriosoma  ............................................................  1055
```

```
Chrysodeixis chalcites ............................................................  1055
Coeliades ramanatek    ............................................................  1139
Meroptera pravella     ............................................................  1139
Salamis anteva         ............................................................  1139
Precis andremiaja      ............................................................  1139

Consensus              GCTCGGCGGGCAGCTTCCGGGAAACCAAAGATTTTGGACTCCGGGGGGAGTATGGTTGCA  1199
Papilio xuthus         ............................................................  1200
Pro. anacardii duprei  ............................................................  1199
Kallimoides rumia      ............................................................  1199
Araschina levana       ............................................................  1199
Junonia stygia         ............................................................  1199
A. jatrophae saturata  ............................................................  1199
Mallika jacksoni       ............................................................  1199
Helicoverpa zea        ............................................................  1199
Helicoverpa armigera   ............................................................  1199
Chrysodeixis includens ............................................................  1114
Chrysodeixis eriosoma  ............................................................  1114
Chrysodeixis chalcites ............................................................  1114
Coeliades ramanatek    ............................................................  1199
Meroptera pravella     ............................................................  1199
Salamis anteva         ............................................................  1199
Precis andremiaja      ............................................................  1199

Consensus              AAGCTGAAACTTAAAGGAATTGACGGAAGGGCACCACCAGGAGTGGAGCCTGCGGCTTAA  1259
Papilio xuthus         ............................................................  1260
Pro. anacardii duprei  ............................................................  1259
Kallimoides rumia      ............................................................  1259
Araschina levana       ............................................................  1259
Junonia stygia         ............................................................  1259
A. jatrophae saturata  ............................................................  1259
Mallika jacksoni       ............................................................  1259
Helicoverpa zea        ............................................................  1259
Helicoverpa armigera   ............................................................  1259
Chrysodeixis includens ............................................................  1174
Chrysodeixis eriosoma  ............................................................  1174
Chrysodeixis chalcites ............................................................  1174
Coeliades ramanatek    ............................................................  1259
Meroptera pravella     ............................................................  1259
Salamis anteva         ............................................................  1259
Precis andremiaja      ............................................................  1259

Consensus              TTTGACTCAACACGGGAAATCTCACCAGGCCCGGACACCGGAAGGATTGACAGATTAACA  1319
Papilio xuthus         ............................................................  1320
Pro. anacardii duprei  ............................................................  1319
Kallimoides rumia      ............................................................  1319
Araschina levana       ............................................................  1319
Junonia stygia         ............................................................  1319
A. jatrophae saturata  ............................................................  1319
Mallika jacksoni       ............................................................  1319
Helicoverpa zea        ............................................................  1319
Helicoverpa armigera   ............................................................  1319
Chrysodeixis includens ............................................................  1234
Chrysodeixis eriosoma  ............................................................  1234
Chrysodeixis chalcites ............................................................  1234
Coeliades ramanatek    ............................................................  1319
```

```
Meroptera pravella        ............................................................  1319
Salamis anteva            ............................................................  1319
Precis andremiaja         ............................................................  1319

Consensus                 GCTCTTTCTTGATTCGGTGGGTGGTGGTGCATGGCCGTTCTTAGTTGGTGGAGCGATTTG  1379
Papilio xuthus            ............................................................  1380
Pro. anacardii duprei     ............................................................  1379
Kallimoides rumia         ............................................................  1379
Araschina levana          ............................................................  1379
Junonia stygia            ............................................................  1379
A. jatrophae saturata     ............................................................  1379
Mallika jacksoni          ............................................................  1379
Helicoverpa zea           ............................................................  1379
Helicoverpa armigera      ............................................................  1379
Chrysodeixis includens    ............................................................  1294
Chrysodeixis eriosoma     ............................................................  1294
Chrysodeixis chalcites    ............................................................  1294
Coeliades ramanatek       ............................................................  1379
Meroptera pravella        ............................................................  1379
Salamis anteva            ............................................................  1379
Precis andremiaja         ............................................................  1379

Consensus                 TCTGGTTAATTCCGGTAACGAACGAGACTCTAGCCTGCTAAATAGGCGTCGTCATTTAGG  1439
Papilio xuthus            ..........................................................T.  1440
Pro. anacardii duprei     ............................................................  1439
Kallimoides rumia         ............................................................  1439
Araschina levana          ............................................................  1439
Junonia stygia            ............................................................  1439
A. jatrophae saturata     ............................................................  1439
Mallika jacksoni          ............................................................  1439
Helicoverpa zea           ............................................................  1439
Helicoverpa armigera      ............................................................  1439
Chrysodeixis includens    ............................................................  1354
Chrysodeixis eriosoma     ............................................................  1354
Chrysodeixis chalcites    ............................................................  1354
Coeliades ramanatek       ...................................................C......T.  1439
Meroptera pravella        ............................................................  1439
Salamis anteva            ............................................................  1439
Precis andremiaja         ............................................................  1439

Consensus                 TGTGCCCGGCTT-CGGTCGAGCAACTCACTGGCGGCGTATTAAAATTCTTCTTAGAGGGA  1498
Papilio xuthus            .....GT.....-......C........................................  1499
Pro. anacardii duprei     ........AT..-...............................................  1498
Kallimoides rumia         ........AT..-...............................................  1498
Araschina levana          ........AT..-...............................................  1498
Junonia stygia            ............-...............................................  1498
A. jatrophae saturata     ........AT..-...............................................  1498
Mallika jacksoni          .........T..-..A............................................  1498
Helicoverpa zea           .....GT...CACGT...AC..............A.........................  1499
Helicoverpa armigera      .....GT...CACGT...AC..............A.........................  1499
Chrysodeixis includens    .....GT....CC--...AC..............A.........................  1412
Chrysodeixis eriosoma     .....GT....CC--...AC..............A.........................  1412
Chrysodeixis chalcites    .....GT....CC--...AC..............A.........................  1412
Coeliades ramanatek       .....T..A...-......T..............A.........................  1498
Meroptera pravella        .....GT.....-.....TC........................................  1498
Salamis anteva            ........AT..-...............................................  1498
```

```
Precis andremiaja        ........AT..-...............................................  1498

Consensus                CCGGCGGCTTCGAGCCGCACGAGATTGAGCAATAACAGGTCTGTGATGCCCTTAGATGTC  1558
Papilio xuthus           ............................................................  1559
Pro. anacardii duprei    ............................................................  1558
Kallimoides rumia        ............................................................  1558
Araschina levana         ............................................................  1558
Junonia stygia           ............................................................  1558
A. jatrophae saturata    ............................................................  1558
Mallika jacksoni         ............................................................  1558
Helicoverpa zea          ............................................................  1559
Helicoverpa armigera     ............................................................  1559
Chrysodeixis includens   ............................................................  1472
Chrysodeixis eriosoma    ............................................................  1472
Chrysodeixis chalcites   ............................................................  1472
Coeliades ramanatek      ......T.....................................................  1558
Meroptera pravella       ............................................................  1558
Salamis anteva           ............................................................  1558
Precis andremiaja        ............................................................  1558

Consensus                CTGGGCCGCACGCGCGCTACACTGAAGGAATCAGCATGTTCTCCCTGGCCTAGAGGCCCG  1618
Papilio xuthus           ............................................................  1619
Pro. anacardii duprei    ............................................................  1618
Kallimoides rumia        ............................................................  1618
Araschina levana         ............................................................  1618
Junonia stygia           ............................................................  1618
A. jatrophae saturata    ............................................................  1618
Mallika jacksoni         ............................................................  1618
Helicoverpa zea          ............................................................  1619
Helicoverpa armigera     ............................................................  1619
Chrysodeixis includens   ............................................................  1532
Chrysodeixis eriosoma    ............................................................  1532
Chrysodeixis chalcites   ............................................................  1532
Coeliades ramanatek      ............................................................  1618
Meroptera pravella       ............................................................  1618
Salamis anteva           ............................................................  1618
Precis andremiaja        ............................................................  1618

Consensus                GGCAACCCGTTGAAACTCCTTCGTGCTGGGGATTGGGGTTTGCAATTATCCCCCATAAAC  1678
Papilio xuthus           ............................................................  1679
Pro. anacardii duprei    ............................................................  1678
Kallimoides rumia        ............................................................  1678
Araschina levana         ............................................................  1678
Junonia stygia           ............................................................  1678
A. jatrophae saturata    ............................................................  1678
Mallika jacksoni         ............................................................  1678
Helicoverpa zea          .........C..................................................  1679
Helicoverpa armigera     .........C..................................................  1679
Chrysodeixis includens   .........C..................................................  1592
Chrysodeixis eriosoma    .........C..................................................  1592
Chrysodeixis chalcites   .........C..................................................  1592
Coeliades ramanatek      ............................................................  1678
Meroptera pravella       .........C..................................................  1678
Salamis anteva           ............................................................  1678
Precis andremiaja        ............................................................  1678
```

```
Consensus               GAGGAATTCCTAGTAAGCGCGAGTCATAAGCTCGCGTTGATTACGTCCCTGCCCTTTGTA  1738
Papilio xuthus          ............................................................  1739
Pro. anacardii duprei   ............................................................  1738
Kallimoides rumia       ............................................................  1738
Araschina levana        ............................................................  1738
Junonia stygia          ............................................................  1738
A. jatrophae saturata   ............................................................  1738
Mallika jacksoni        ............................................................  1738
Helicoverpa zea         ............................................................  1739
Helicoverpa armigera    ............................................................  1739
Chrysodeixis includens  ............................................................  1652
Chrysodeixis eriosoma   ............................................................  1652
Chrysodeixis chalcites  ............................................................  1652
Coeliades ramanatek     ...........................................A................  1738
Meroptera pravella      ...........................................A................  1738
Salamis anteva          ............................................................  1738
Precis andremiaja       ............................................................  1738

Consensus               CACACCGCCCGTCGCTACTACCGATTGAATGATTTAGTGAGGTCTTCGGACCGACACGCG  1798
Papilio xuthus          ............................................................  1799
Pro. anacardii duprei   ............................................................  1798
Kallimoides rumia       ............................................................  1798
Araschina levana        ............................................................  1798
Junonia stygia          ............................................................  1798
A. jatrophae saturata   ............................................................  1798
Mallika jacksoni        ............................................................  1798
Helicoverpa zea         ............................................................  1799
Helicoverpa armigera    ............................................................  1799
Chrysodeixis includens  ............................................................  1712
Chrysodeixis eriosoma   ............................................................  1712
Chrysodeixis chalcites  ............................................................  1712
Coeliades ramanatek     ............................................................  1798
Meroptera pravella      ............................................................  1798
Salamis anteva          ............................................................  1798
Precis andremiaja       ............................................................  1798

Consensus               GTGGCTTCACGGCCG--TCGGCGTTGCTGGGAAGTTGACCAAACTTGATCATTTAGAGGA  1856
Papilio xuthus          .......A.......--.......C...................................  1857
Pro. anacardii duprei   T..............--...........................................  1856
Kallimoides rumia       A......A.T.....TG..T........................................  1858
Araschina levana        ...............--...........................................  1856
Junonia stygia          ...............--...........................................  1856
A. jatrophae saturata   ...............--...........................................  1856
Mallika jacksoni        ...............--...........................................  1856
Helicoverpa zea         ...............--...........................................  1857
Helicoverpa armigera    ...............--...........................................  1857
Chrysodeixis includens  ...............--...........................................  1770
Chrysodeixis eriosoma   ...............--...........................................  1770
Chrysodeixis chalcites  ...............--...........................................  1770
Coeliades ramanatek     ........G......--...........................................  1856
Meroptera pravella      ...............--...........................................  1856
Salamis anteva          ...............--...........................................  1856
Precis andremiaja       ...............--...........................................  1856

Consensus               AGTAAAAGTCGTAACAAGGTTTCCGTAGGGGAACCTGCGGAAGGATCATTAACGTGTTAC  1916
Papilio xuthus          .......................................................AA..T  1917
```

```
Pro. anacardii duprei  .......................................................A..TT  1916
Kallimoides rumia      .......................................................TA.CG  1918
Araschina levana       .......................................................AG...  1916
Junonia stygia         .......................................................AACGT  1916
A. jatrophae saturata  .......................................................AA...  1916
Mallika jacksoni       .......................................................AA.TT  1916
Helicoverpa zea        .........................................................A.A  1917
Helicoverpa armigera   .........................................................A.A  1917
Chrysodeixis includens ............................................................  1830
Chrysodeixis eriosoma  ............................................................  1830
Chrysodeixis chalcites ............................................................  1830
Coeliades ramanatek    .........................................................G..  1916
Meroptera pravella     .........................................................ACA  1916
Salamis anteva         .........................................................ACG  1916
Precis andremiaja      .......................................................TA.CA  1916

Consensus              CGTTACWCGCGCGTGCSTTRCGAARATGKTRTTA-AYVSNRAAAAAA-AATACA------  1968
Papilio xuthus         ATA.TATTTTATTAATCG.AAA.TA..AA.A..TACCGAAA.T..TCC.CCTTTCTT---  1974
Pro. anacardii duprei  ACGACGCTT.TGTGTTG.AATCT.A.C.C.AAA.G.CACCA.....TG..A..-------  1969
Kallimoides rumia      TA.CGGA...T.TC..GAGGG...G..ATAACG.G.CGCTT..T.G.C.CA.A.AAA---  1975
Araschina levana       GTA.TAT.AATATC.AA.GATTGCG...A.A.G.A.TACGAT.-------------A---  1960
Junonia stygia         .TC.T.T.T.A..AAGCCGT.TTCTCG.CAGAGT-GTGTAG...CG.CG.ACGCTAA---  1972
A. jatrophae saturata  GA.A..CGAACTA.AACACG.TCGG..AT.G...-.GCGCT..T.G.T.TCTATCTC---  1972
Mallika jacksoni       ATG...GACTCA..TTTA.AAACTCGA.A.TG..T.TAAAG...CG.TT.ATAGACA---  1973
Helicoverpa zea        ..CA.GA....AC..GC.CG...CGCGCG.G...T.ACGTG..C..T-...C..------  1970
Helicoverpa armigera   ..CA.GA....AC..GC.CG...CGCGCG.G...T.ACGTA..C..T-...C..------  1970
Chrysodeixis includens ....TTCAA..T.C..TCGCGC.TACG.GAAAC-----------G.T-G.....------  1872
Chrysodeixis eriosoma  ....G.G.....ACA.GCCG....A-------C-----------G.T-G.....------  1865
Chrysodeixis chalcites ....G.G......AA.GCCG....A-------C-----------G.T-G.....------  1865
Coeliades ramanatek    ..AG.GTTAAATC.CGA..AAA..T.CATAGA..ATGTA-C.TCTG.C.CA...ACTTGC  1975
Meroptera pravella     .ACG..A.A..A.ACTT..AGTT.CG.TG.TG.TGTTGTATGTGT..-....ATCCATAG  1975
Salamis anteva         TA...TAATAA..C..AC.CG.CCCTG.T.CGAGACACGTG.T.TT.TG..TAC------  1970
Precis andremiaja      TCG.TAT..T.....G..CGT.GTG.CCGCG.CTCCACGCG.CCTCG.....CCGCTC  1976

Consensus              --T--------A---CATCT-CACAAATGAACATATNAAACDN-TCGWACA-AAMKACRA  2012
Papilio xuthus         -----------T-TG.G.GG-TGTGTG..TGTG.G.GTGTGTGTGT.TGTGT..AT.AA.  2021
Pro. anacardii duprei  -----------G-AC.....-.GA.TT.CG..TCGAG.CC.GCAA..T..G-..CTCAC.  2015
Kallimoides rumia      --C------AC.-CT..C.A-TC...GA..GA.GG.G....GAT.TCG.AT-.CTTCGA.  2024
Araschina levana       --.------AG.-TAT.A.A-..A..C.T.......CC.CGTGA...T.A.-..TATATT  2009
Junonia stygia         --.------AG.-CA..AAA-..A...C.....ACAAG.T.CAA...A..G-..TTT.G.  2021
A. jatrophae saturata  --G------AA.-CA...AA-T.A..TC....T.---.CGAAAT...T..G-..ATT.G.  2018
Mallika jacksoni       --C------GA.-TC..AAA-..A..-G..T.TC---G.CGAACGATAC.G-.CTAT.GG  2018
Helicoverpa zea        -CA------------.CA..ATG.GC...C.T....TGC.TC--G..C.T.TCCAG.GG.  2015
Helicoverpa armigera   -CA------------..C.ACT.G.-----------------------------GGAC.  1987
Chrysodeixis includens -T.------------...G.A.T.C..AC.CA...ACG..TG--.TAC...TTCGA..Y.  1917
Chrysodeixis eriosoma  -T.------------T..G.A.C.CG.AC..AC.T.T.T.TA--AAAT.G.G.CCG.TAC  1910
Chrysodeixis chalcites -T.------------T..G.A.C.CG.AC..AC.T.T.T.TA--AAAT.G.G.CCG.TAC  1910
Coeliades ramanatek    AAAATATTATTCGAG.G...TAC.C.C.C.TTT.GGACGC.GGG...AT.GG..CG.TC.  2035
Meroptera pravella     ACA-------T.AAC.TATGTGTGCT...---T-----------G--A.G.T.CAGT.G.  2012
Salamis anteva         -GG-------T.ACG.TAA.AG......A---C..CT.--.TAC.--ACT.CT.CT..T.  2015
Precis andremiaja      GAG-------G.CGG.G.ACAACGCT...---...GAC--ATACA--ACA.CG.AA..A.  2022

Consensus              CRMATACGMRCN-GKACGC-CG--CNTTGAAACRCCTTCGN------CGTGCTCCGGS--  2060
Araschina levana       AAC.CG.ACG.AC.C....A..--.A.GC.CG.-----ACGCACGCA..C..C.ATCGGG  2074
Pro. anacardii duprei  .GC.CGTACGGC------------.GCGACG..GAG....TTGCGCG..C..G...CGTA  2063
Kallimoides rumia      GGAGA.TCGAGAAAAG.T.T..GCGCA.ATCCAATAG...TCGTCGA..-----------  2073
```

```
Papilio xuthus         TAAT..TTATAT--------------A.A..C.ATTAC.CTGGACGGT.G..GT..TGTA  2055
Junonia stygia         GAC..CGTGT.GA.ATTCGA.TCG.-A.CTCG.A..GCG.CAGCA-----..AG...CGC  2075
A. jatrophae saturata  ACG..CGCCTAAC.T.TTGT..ACGT...TCGAA.A.AT.TACGACG..A....ATCCTA  2078
Mallika jacksoni       .GCTC...CA.AC.CG.A.T.TTCGAG.T..C.GTG.C.ATACGAAAA.GA.C.AA.---  2075
Helicoverpa zea        .AC.G.GTCGT--.G....-T.TG.G.....TAG..GA-----------..A.T...T--  2059
Helicoverpa armigera   .AG.GT..AA.--.G....-T.TG.G.....TAG..GA-----------..A.T...T--  2031
Chrysodeixis includens TAG.G...--------------------------------------------CG...C--  1931
Chrysodeixis eriosoma  GGA.CG..AG.--.TGTAA-GA--GC..T....AGT..-----------.A.G.GC.C--  1952
Chrysodeixis chalcites GGA.CG..AG.--.TGTAA-GA--GC.......AGT..-----------.A.G.GC.C--  1952
Coeliades ramanatek    .GC.G..ATCATGAG.GTA-ACGC.C.C.TC.TAG..C..TACA---A.ACG.AGACTGG  2091
Meroptera pravella     .CGG..AAAATCC.C.G..-..CG-------T.G.A...--------.A.AGC.GTATAC  2057
Salamis anteva         .TAT..TATATAACT...T-..AC------G..GA.GA..ATGATCA..AA..T.TCGAA  2068
Precis andremiaja      ACAG.T..TCTTGCGCGCG-..CG.C.C.....G.GCG..CCTCGAC...AA.TATCGAA  2081

Consensus              ---GAAGAWCGTACVGCTCGCGCGTCGTAACKT--TC--------GCCCGG-AAYG----  2102
Papilio xuthus         ACAA.CC.AA.CGG------G....TC...TACAA.A--------AGTA.AA..T.----  2117
Pro. anacardii duprei  TCCA..ACGAAA.GA..GA.....TT.C..G.CGAGA--------.AA..A-C.CA----  2111
Kallimoides rumia      -AC..C.TA..C..AC.ATAGAGACGAGT.TTC--G.G-------CGGG.T-TTC.----  2118
Araschina levana       GGGA....GT..TTGCACACG..AG..GT------------------G.AA-.CAC----  2092
Junonia stygia         GAC..C..GAT.CGC.AC.T..TA..CGCG.G---------------TGAA-..C.----  2115
A. jatrophae saturata  TCCA.C..AA...AACAA...A...T.ACG.GC--..G-------C..G.T-CTCC----  2124
Mallika jacksoni       ---C.C.TA..C..GCGCGTAA..AT.GC.ACG--GAA-------A...T.-CTAT----  2118
Helicoverpa zea        ---.TT.TT...CGC.....TC......T.TG.GT..TCGC----.T.GC.TC.T.----  2108
Helicoverpa armigera   ---.TT.TT...CGC.....TC......T.TG.GT..TCGC----.T.GC.TC.T.----  2080
Chrysodeixis includens ---TGTCGT...G.GT......TCG.....GT.GA..ACAC----..TTC.TTTT-----  1979
Chrysodeixis eriosoma  ---.CT..T...T.GT......TCG....TGT.-------------.ATTTTGT.C-----  1992
Chrysodeixis chalcites ---.CT..T...T.GT......TCG....TGT.-------------.ATTTTGT.C-----  1992
Coeliades ramanatek    TT-...ATC..C..CA.GA.TC..GA.CGC.GACG.TGCGCGCGCA.G.T.A..TAATAT  2150
Meroptera pravella     TT-.T.TGCG.-TTA.AG..A...CTT.T..C.CCCTTCG-----.GG...A.CT.YGCT  2110
Salamis anteva         TT-CG..CC.C-.TC.A...AT..AG--.GATACG.A--------.A....T..GAATCC  2116
Precis andremiaja      TC-...A.A.T-.TAA.GAAACAA..--..AAACCATT-------A...T.G.CG.TGGA  2130

Consensus              --------N-NA-NT--N-NTTTTNNN-NGTN-N--N-----N--K----TC----GATA  2130
Papilio xuthus         --------AGG.GG.GTCAC.AA.CCGGT.ACATCTCC--CAGTCCCGGTC.----....  2163
Pro. anacardii duprei  --------GAG.GG.TCCCCG...AGATC..CCCTTCG--TATTGTATCG..----...T  2157
Kallimoides rumia      --------TTTT-A.--TTT..CGGAAGC..TGTTTCGTGCGACCGTACG..----....  2163
Araschina levana       --------TCTCTTC--CAC.ACACGCCCC.TCC-----CCATCGGAAGGAT----C..T  2133
Junonia stygia         --------CAACGGC--GCGAG.ACGCGC..TCC-----GCATCGGACACGT----WKSR  2156
A. jatrophae saturata  --------TTA.GA.--CCCG...CGTA-------------TTCGTTTAT..----...T  2157
Mallika jacksoni       --------TTA.GA.--CGA.C..CGAAC..TTA-----TATCGTGAACG..----..AT  2159
Helicoverpa zea        ----------------------------------CGC--------ACGACA.GC--AGA.  2124
Helicoverpa armigera   ----------------------------------CGC--------ACGACA.GC--AGA.  2096
Chrysodeixis includens ------------------------------------------------------------  1979
Chrysodeixis eriosoma  ------------------------------------------------------------  1992
Chrysodeixis chalcites ------------------------------------------------------------  1992
Coeliades ramanatek    GAAGAATATAT.AAATTTAA..A.TTTATA.ATTAACGCGGTTTTCGGAA..GTAC.C..  2210
Meroptera pravella     CTTTCTAAGAT.TA.ATATT....TTAAAT.TTTAATTTTTTTTTTT--A.--ACAA.A.  2166
Salamis anteva         GCGCCGCGAGT.TC.CTTTG..CCGATGG..GATCGC--------C----------AG.C  2158
Precis andremiaja      YCWSYGGAAGG.TCATTAACG..ATCATC..TATCGTGCGTGCGTT----------CG..  2180

Consensus              RTGTACGTTTCTAYAAT---CATNNAANANNNATCCGCA-NTCCCGGAGTGKTACGTCG-  2185
Papilio xuthus         A.A.GATGGG.GCT.TA---..AAA..T.AAA..A.CA.-----AACC.GTT..GAAA.T  2215
Pro. anacardii duprei  A..ATAT...A..TG-.---...AA..T.TCAC--GT..-----.TTG.C.T.CGT...T  2206
Kallimoides rumia      G..AT-----A.TTT..---...AA..T.TCGGCGTCGTCCC.A.---------------  2200
Araschina levana       AACGTA..ACG..TT..---.--------------------------------.TA...A  2159
Junonia stygia         .WWATGA.A.T.TAT..---...AA..T.TCACGT.C.GCCCGG...C.CCCGT.....C  2213
```

```
A. jatrophae saturata  A..ATAT...A..-T..---.G.AA..T.TCATA.GT..CT.GG...CTC.C---....T  2210
Mallika jacksoni       A..ATAT...A.TTT..---...AA..--TATCG.GT..CT.GG..A.A.TC..TT...T  2214
Helicoverpa zea        GG....AGA..ACA..A---------------.....G--....C.....TCCGC-AC-  2165
Helicoverpa armigera   GG....ACAAAACAC.AAA-------------.....G--....C.....TCTGCAT.-  2140
Chrysodeixis includens -AA....G..TA.C.TAAA-------------.......-AA..GT...AAG.GTA.TA-  2023
Chrysodeixis eriosoma  -.A....G..TA.C.TAAA-------------.......-AA..GT.....G...T.G--  2035
Chrysodeixis chalcites -.A....G..TA.C.TAAA-------------.......-AA..GT.....G...T.G--  2035
Coeliades ramanatek    TG.CG.CCGC..TT.T.CGTG.CCTGTA.CAACAGTTA.CG..TG..--C.G.G.....-  2267
Meroptera pravella     T.A..AACCA...CC.CAGT.GACCGGT.AAA.......GGC.G..TC.CAT.CGCATA-  2225
Salamis anteva         GC......G...CC.C.ACGTG.TCGTG.TAAG.T..GG-A.AT..WSKWMG.T..GTC-  2216
Precis andremiaja      G...C..CG...CC.CGCGA.C.CG..T.CA-CCG.T.G-AGGA...C..ACA...CTA-  2237

Consensus              -----CGTGACGTTAAAMAAAAACCAA----ACGTCGCG--CGMCGCG--G---------  2223
Papilio xuthus         GTGAA.ACA...A----------.G.CG---...A..AC--GACGA..AC.ACGACGACG  2260
Pro. anacardii duprei  -----...........A.....T...T---C-......--..CAC..AGAGGTGCCCGG  2255
Kallimoides rumia      --GGGG.C.........A.T..G..C.T---........--..CGC.CAAA---------  2244
Araschina levana       ATG---A.TG..A.G.TATG...T------------------------------------  2180
Junonia stygia         CGTGT............CC.T.TGTCGC---G..CAC.------------.---------  2249
A. jatrophae saturata  CGT-----.........A.....AA..A---.AAAAT.T--TTATA---A.---------  2248
Mallika jacksoni       CGT-----.........C.....T.CTA---TA......--T.CAC----.---------  2251
Helicoverpa zea        ---GG..C.TTCG.T..C..TC.AAC.---C.T...C.TTG.AA....GT.GGA-TAATA  2218
Helicoverpa armigera   ---CA..C.TTCG.T..C..TC.AAC.---CGA...C.TTG.AA....GT.GGA-TGATA  2193
Chrysodeixis includens ---TA..C.TTCG.T..C.TGC...GT---CT.....ACGATTA....GT.TTC-GTCGA  2076
Chrysodeixis eriosoma  -------.ACGCG.TCGTT..TC..GT---CT.....ACGATTA....GT.TTC-GTAGA  2084
Chrysodeixis chalcites -------.ACGCG.TCGTT..TC..GT---CT.....ACGATTA....GT.TTC-GTAGA  2084
Coeliades ramanatek    ---TA....TTC.G...AG.T...A..AACA..A.T...TA..CGT..GA.AGTTCTATG  2324
Meroptera pravella     ---GC...ATAC..GT-----.TG.GGT----TAGA...AG..CTTT-------------  2260
Salamis anteva         ---GT..........-----..AA..AAAA.AAAAA.ATG..T...-------------  2255
Precis andremiaja      ---TGATA...A-..C-----...A.CGAAA..AAACA.TT..T.--------------  2273

Consensus              ------TW----------TTGCGCGCGVG--TA--N-------------CWCT-------  2243
Papilio xuthus         ACGTTG.T----------G.TGTT.TTGTTG.---T-------------ATTATT-----  2289
Pro. anacardii duprei  CCGTTAAT----------.AT...ATTATATC.TAA-------------.T.GAT-----  2287
Kallimoides rumia      ------CA----------CC.......GCCG..CAT-------------TTTAAA-----  2270
Araschina levana       -----------------------A..ATAA..GAT-------------AT---------  2194
Junonia stygia         ------.T----------CG.T..C..GCCG..ATT-------------AT.GAA-----  2275
A. jatrophae saturata  ------AT----------..TAA.AAAAAAA..TTT------------------------  2268
Mallika jacksoni       ------.A----------AC.T..C..GCCG..CGT------------------------  2271
Helicoverpa zea        TCATTT.CTTTTTTATACA.A.A.A.AT.TA..TAAAATACAACA----.A.A-------  2267
Helicoverpa armigera   TCATTT.ATTTTTATACA.ACAAACACACGTGTATATAAAATACA----.A..-------  2242
Chrysodeixis includens CGGGGT.TCTGTTGA------------------------AAAAT----------------  2096
Chrysodeixis eriosoma  CGAGATAATTTATAT------------------------ATTTTT----AA..-------  2109
Chrysodeixis chalcites CGAGATAATTTATAT------------------------ATTTTT----AA..-------  2109
Coeliades ramanatek    TTGAGCCGCGACATATGC..A.....CC.AAC.TAAATTGAAACGAACA.AA.ATCTAAC  2384
Meroptera pravella     ----TACCTCCCTTCGGGGC.GAACT.C.--------------------.T..TTCTAA-  2295
Salamis anteva         ----GCGCACCGTTCGCGG...C..ACC.--------------------TAA.TATCGA-  2290
Precis andremiaja      ------------------.........C.--------------------.G.CTC--GA-  2292

Consensus              ----------N-----ATTM-T------TATWTATATATATTATTTAATTAARMATGAAT  2281
Papilio xuthus         GTTGTTG--CTGTTCGC----------C.TCTA.G.C..T..T...TT..TTTT.CAT.C  2337
Pro. anacardii duprei  CGAGTCG--ATCGTCCG.ATC.CGTGTG...CACG..C..CG.CGACGA.G.AATC...A  2345
Kallimoides rumia      CGGTCAA--TTTTTTTT..TT.AATATA.T.T.T.T.T....GGA...AG..AA.AA..A  2328
Araschina levana       ----------AACACA.AACT.A----AC..A.CC.CG.GA.CG.A..AAT.TAT.T...  2240
Junonia stygia         TCAT------AAACAC.A.CT.GTGAGTGTGA...CGCCG..CAACGCGCGCGATATC..  2329
A. jatrophae saturata  ----------TAAATTGA.TT.AT---AA..CG...ATATCGT.C.C.AC..GA.C..T.  2315
Mallika jacksoni       ----------TATGTT.A.CA.AT---C...A....AT--AA.GA.G....TGTTAT..A  2316
```

```
Helicoverpa zea          ---------C------.CAAG.------CTATATC..G..AC.C.AG....CGCGAAC.G  2306
Helicoverpa armigera     ---------C------.CAAG.------C.AT.TA..G..AC.C.AG....CGC.AAC.G  2281
Chrysodeixis includens   ----------------------------.T.T.T..ACAT.C..A..C.GT.AC...GG.  2128
Chrysodeixis eriosoma    ---------A------T.AAA-------A.AA......C.....A..C.GT.AC...GG.  2147
Chrysodeixis chalcites   ---------A------T.AAAA------A..A......C..A..A..C.GT.AC...GG.  2148
Coeliades ramanatek      CTATGTGACGACCGAT..GCG.CGTGAGCGAGC.CC..ACCGTGCG.GCG.TCGTC.CG.  2444
Meroptera pravella       ---GA----TATAT--.-.T--------.T.T.T.TA.AT..T..A....TTTTT.T.TA  2337
Salamis anteva           ---ATTTATTCTACAT...A--------...A...GGG.GG.GA..TT..G.TG...TG.  2339
Precis andremiaja        ---AACGCGCGCG---CC.C--------G.CCGTA..TATCG.A.CG.AA..CT..A.CG  2338

Consensus                WAATAAATAAAAACCATTACCCTGGACGGTGGATCACTTGGCTCGCGGGTCGATGAAGAA  2341
Papilio xuthus           AT.....A.G....A.............................................  2397
Pro. anacardii duprei    TC.C..CA.C..................................................  2405
Kallimoides rumia        A..ATTCAC...................................................  2388
Araschina levana         T.T..T...T..................................................  2300
Junonia stygia           GC.AC.CC....................................................  2389
A. jatrophae saturata    AT.A.TCGT...................................................  2375
Mallika jacksoni         C.T.TC..CG..................................................  2376
Helicoverpa zea          CGG...T.T.....T.............................................  2366
Helicoverpa armigera     CGG...T.T.....T.............................................  2341
Chrysodeixis includens   T.....T.......T.............................................  2188
Chrysodeixis eriosoma    T--A.T..T.....T.............................................  2205
Chrysodeixis chalcites   T--A.T..T.....T.............................................  2206
Coeliades ramanatek      GTTGTC...T....A..C..........................................  2504
Meroptera pravella       C..A..T..T......C...........................................  2397
Salamis anteva           A...C..C....................................................  2399
Precis andremiaja        A..C..TC....................................................  2398

Consensus                CGCAGTTAACTGCGCGTCATAGTGTGAACTGCAGGACACATTTGAACATCGACATTTCGA  2401
Papilio xuthus           ............................................................  2457
Pro. anacardii duprei    ............................................................  2465
Kallimoides rumia        ............................................................  2448
Araschina levana         ............................................................  2360
Junonia stygia           ............................................................  2449
A. jatrophae saturata    ............................................................  2435
Mallika jacksoni         ............................................................  2436
Helicoverpa zea          ............................................................  2426
Helicoverpa armigera     ............................................................  2401
Chrysodeixis includens   ............................................................  2248
Chrysodeixis eriosoma    ............................................................  2265
Chrysodeixis chalcites   ............................................................  2266
Coeliades ramanatek      ............................................................  2564
Meroptera pravella       ............................................................  2457
Salamis anteva           ............................................................  2459
Precis andremiaja        ............................................................  2458

Consensus                ACGCACATTGCGGTCCGTGGAGAAACATCCAGGACCACTCCTGTCTGAGGGCCGGCTGTA  2461
Papilio xuthus           .........................T.................................T  2517
Pro. anacardii duprei    ............................................................  2525
Kallimoides rumia        ............................................................  2508
Araschina levana         ............................................................  2420
Junonia stygia           ............................................................  2509
A. jatrophae saturata    ............................................................  2495
Mallika jacksoni         ............................................................  2496
Helicoverpa zea          .......................C....................................  2486
Helicoverpa armigera     .......................C....................................  2461
```

```
Chrysodeixis includens .......................C....................................  2308
Chrysodeixis eriosoma  .......................C....................................  2325
Chrysodeixis chalcites .......................C....................................  2326
Coeliades ramanatek    .......................T....................................  2624
Meroptera pravella     ................C......C......G...........................C.  2517
Salamis anteva         ............................................................  2519
Precis andremiaja      ............................................................  2518

Consensus              TAAAAATATAWATCACACTGTACSAAGTCTNGTYGGAGASTAYADHGTATCGGTYGCGGY  2521
Papilio xuthus         A...T...A.A..........T.TTCCA.CAT.T.A.TTG.GATGA.AGAACAATTGA--  2575
Pro. anacardii duprei  ..........T..........G.GC.CGTAT..GC.TACA..TGGC.GTGTCCGC.TTTC  2585
Kallimoides rumia      .....G....T............GTC...ATCGA.CGATC..C.TGTG.CG.TCTC..CG  2568
Araschina levana       .....T....A..........T.ACGTGT.AACCAC.CGAG.ACATA.GA....TT.C.T  2480
Junonia stygia         ..........T............G.GTGGCG..A.T.CGCCGCTCC...CATA.GA...C  2569
A. jatrophae saturata  ..........T............GCTT.A.GCGC.TTA.A.TA.TTT.T.---.C.TATA  2552
Mallika jacksoni       ..........T............G..AAA.AA.TT..TTT.TCGTACATA---.GA...T  2553
Helicoverpa zea        .....CA.C.ATG.CACA.TGCGCG..CGCGCGC.C.C.CG.TTAACAC...TGT.T.T-  2545
Helicoverpa armigera   .....CA.C.ATG.CACA.TGCGCG..CGCGCGC.C.C.CG.TTAACAC...TGT.T.TG  2521
Chrysodeixis includens ....GTA.ACATG.CACA.TGCGC.C....C..TC....CG.GCG--C..AT.AC.GT.C  2366
Chrysodeixis eriosoma  ....GTA.ACATG.CACA.TGCGC.G....C..CTAC..GACT.GC.C..TT.AC.GT.T  2385
Chrysodeixis chalcites ....GTA.ACATG.CACA.TGCGC.G....C..CT.C..GACG.GC.C..TT.AC.GT.T  2386
Coeliades ramanatek    ......C.A.T.C........T.A...CGCTCGTC.C..TG.ACAT..GA....TC..TC  2684
Meroptera pravella     ......C.A.G.C.......C..GT.CAA.ATAT.T.T.T.GT.TGT.GCATA.GA...T  2577
Salamis anteva         ..........T............ACT..GCC.CTC.CTCG.TCGTTCGT...T.C.TTCG  2579
Precis andremiaja      ....T.....C.C..........CGCAC.GTTCGCTC.CGCTCGAGAG.G..CGGCGC.C  2578

Consensus              TTCCGCGGTTGCG----NNRCGTTTCTGGGTC---N-------RCCGC--N-----N--C  2558
Papilio xuthus         CGGTT.C....ATATAACAAAAA----------------------------------AAA  2601
Pro. anacardii duprei  GCG.T..C.C...A-----------------------------------------CTCG.  2604
Kallimoides rumia      AGAG.AACGA...----------A.T.C.CG.-----------GTTA.GTTCTCG----.  2603
Araschina levana       ...TC..TA..T.CAT---G...A.A.----A-----------AT----ATATCTTA--T  2516
Junonia stygia         G.......-------------..CG..T.CGA-----------CT...------------  2593
A. jatrophae saturata  .GAA...T.ACAAATGACGG..---------------------------------TCCG.  2579
Mallika jacksoni       .....TT.A.CGTGTTCGAG...CCA.C.CGA-----------CGA..GATAACGATCA.  2602
Helicoverpa zea        -CG.....CG...CACATGA..G.....C...AACGGGCGCGCA....GTTT------G.  2598
Helicoverpa armigera   .CG.....CG...CACATGA..G.....C...AACGGGCGCG-C....GTCT------G.  2574
Chrysodeixis includens .C.GT.....CGTCACGTTCGA..GGGT.C.T--CGCGTTCGCG.GT.GCTCTCGATCTT  2424
Chrysodeixis eriosoma  .....TC.A.C..TCTCGTA..A.AAGC..G.GGCGTTCGCGCG....CCTCAACGTCGT  2445
Chrysodeixis chalcites .....TC.A.C..TCTCGTA..A.A.GC..G.GGCGTTCGCGCG....CCTCAACGTCGT  2446
Coeliades ramanatek    GAT.TGT..GT------CGATAGG..C.TT.AAAAATTTTATTATTTTCTATTA--C---  2733
Meroptera pravella     .C.AA.CAC--------ACG.TCGCG..T...AGGTCCGTTTAAATA------T--A---  2618
Salamis anteva         ...GTTC..--------TCGTT.G.GC.T..GAGAGTTTTTTCA.AC------T--C---  2620
Precis andremiaja      -----AC.A--------GCG..CG.ACACA.GACGGCGTCCGCG.GA------C--C---  2614

Consensus              GGTCCGTT-------CAAATRTCCGTTCAAANATAGCACGCT-NNCCG---TTHGR-TNT  2606
Papilio xuthus         ........-------T....A.TAA..T.T.T...TATATA.ACA.AC---ACAAACACA  2651
Pro. anacardii duprei  ........-------.....A.AACGAA.CCG..GCA....GTTCAGT---ACTCG-.A.  2653
Kallimoides rumia      ........-------.....A.TATA.ACT.TTG.T.GTT.G-AAG..-----------.  2644
Araschina levana       ATAT...A-------T.C--A.GT.CATCGGCGGT..TTA.G-GT...---..TAA-ATA  2562
Junonia stygia         ........-------.....A.A..AATT.CCGAT..T.TA.---G..---..TAC-G-.  2638
A. jatrophae saturata  .TAA...A-------TTGG.A.ATCGAATC------G.T...---AT.---.GTAC-G-.  2618
Mallika jacksoni       ........-------.....A.ATCGATCC------G.T..G---A..---------C-G  2636
Helicoverpa zea        ...GG..GCTCGTCGGCGT.G..........T....T...G.TG-------..GTAT.GC  2651
Helicoverpa armigera   ...GG..GCTCGTCGGCGT.G..........T....T...G.TG-------..GTAT.GC  2627
Chrysodeixis includens .YG.GACGGATATTT.G.CGG..........A...A.G.T..ATA.GATAGA.A.GACA.  2484
Chrysodeixis eriosoma  .AGTGACGGATCAAT.G.CGG..........A...A.G.T..ATA.GATTGA.A.GACA.  2505
```

```
Chrysodeixis chalcites .AGTGACGGATCAAT.G.CGG.........A...A.G.T..ATA.GATTGA.A.GACA.  2506
Coeliades ramanatek    -A..AC.A--CGCGTAGT..G.GTA----GCGTA.TTC..GG--CATCCGTG.C.AT.TC  2784
Meroptera pravella     ---TA.A.--CGTGTA.G..CG.T.----.C---.CACG.G.GGCT..CCTCAC.GC.CG  2666
Salamis anteva         -ACGA.CA--CGACGACG.CGA.GA----CGACG.CG...ACGACGACGGCG.CTTCGTC  2673
Precis andremiaja      -...GCCA--CCG-----------------------..TCGCTT.CGGT..CTTC.C.  2646

Consensus              GTGTATGTAC-CGTGTGCRTCGC---GTGTGCTCT----------T--A-A-N-N--GCG  2645
Papilio xuthus         AACG.GA..T-..ATC..AAAA---TA.A.A..A.TATGCGAT-------------C.G.  2694
Pro. anacardii duprei  ..A..C....-.CGTC..GA.A.GTC.C...TCA-----ACG--------------C...  2693
Kallimoides rumia      T.C.G.....-GT.AC..G..AA--C...G.AG---------------CG.GTGTTGTT.  2686
Araschina levana       T....AA...-GA....TG.ATGTTTA...A.A..TGCGTGCTTG.--.T.TATTTC..T  2619
Junonia stygia         A.........-TCGTGT.G.GT.--GACACCG..--------GTG.GTCTGCACACGCGT  2687
A. jatrophae saturata  ..........-GTGT..TC----------ACGCG.TACGACGTCG.GT.ATATATACT..  2667
Mallika jacksoni       A..CG.....-...A...G.GTA--C...AAACGGGATTACGTCGAGT.T.CCGTGGA..  2693
Helicoverpa zea        .......C.TT.....CGAA...GCT.......GCGCGCCGCCC.TA.-.AAAG--...  2708
Helicoverpa armigera   .......C.TT.....CGAA...GCT.......GCGCGCCGCCC.TA.-.AAAG--...  2684
Chrysodeixis includens T...CGAACGG.T....GAA...GCGT.AA.AA.A-------CCG.AA.-.AGTGATCRA  2536
Chrysodeixis eriosoma  T...CGAACGA.T...AAA....TAATGCGTTGT.-------AAT.AA.-.CAATAACGT  2557
Chrysodeixis chalcites T...CGAACGA.T...AAA....TAGTGCGTTGT.-------AAT.AA.-.CAATAACGT  2558
Coeliades ramanatek    ..TCG.CGTACGT.CGC.GCGAG---...GA...ATCG---------------------  2819
Meroptera pravella     CAACCGAAG--..AC.CTTCG.A-----C.A..G.CAT---------------------  2697
Salamis anteva         ..AC..A.GACG....TGTC...---....CG..GCCG---------------------  2708
Precis andremiaja      A.CCGA.A.GAA..CC.GTGA..---.-CC..CGGTGC---------------------  2680

Consensus              TTCGTGCGTMCGTCGCTCCG--GAC--A-AWN-ATCGCGACGT-GAGG------TCGAYY  2692
Papilio xuthus         GAA.G....C.C.G.GGGAT--.CG----CGACTCTT......AA.AA------....CA  2742
Pro. anacardii duprei  .C.......A.......GA----G.----.T--T...T....AC...A------G..GTG  2737
Kallimoides rumia      ..GTCA.A.CGCA...A.AC--ACA----CG--.K...---..RTY..------Y..T.T  2729
Araschina levana       ..TTGCTC.--.CGATATT.--ATA----TT--.CGTGC..A.G.C.A------G..TGT  2663
Junonia stygia         .G.......A.......T..--AG.----CT--CC.......AC...A------G..TTC  2733
A. jatrophae saturata  .A.......A.......GA----G.----CT--TC..T..A.AC...A------G..TTC  2711
Mallika jacksoni       ...T.....A......CTA----TA----TA--CG.....GT-----------A-----  2726
Helicoverpa zea        .G..C....GTACGCG.G..CG...GC.C.AAGG.A...G...ACGT.AGTGTG.AT.CC  2768
Helicoverpa armigera   .G..C....GTACGCG.G..CG...GC.C.AAGG.A...G...ACGT.AGTGTG.AT.CC  2744
Chrysodeixis includens CGACACGC.TTACACA.AGACG...GT.C.ATG.......-------.TATGCG.AT.AC  2589
Chrysodeixis eriosoma  A.ATGCG----T.TA...GACG...GT.C.AAG.......-------.GATGCG.TT.AT  2606
Chrysodeixis chalcites A.ATGCG----T.TA...GACG...GT.C.AAG.......-------.GATGCG.TT.AT  2607
Coeliades ramanatek    ----A.TTACGTGTTGA...TGCG.GACCCTTCGGT.T.CGTGT...AATATTT.AT.--  2873
Meroptera pravella     ----.....A..GTATA..AGA..GTG.-GTTCGGTCGAC...C..C.G-----------  2741
Salamis anteva         ----....ACTCG..G....TTC.AAA.-TATT.-------------CATTSRWYRCCGA  2750
Precis andremiaja      ----.....CGCG..G....TTC.AAT.-TTACGATAAACAY.S.MT.MTCGTKMN.CGT  2735

Consensus              CGTNTC-G-ATGCN-AGNGTTTCATGC-CGTTCGCGACCCTTCGGTGTRATN-NATACCA  2747
Papilio xuthus         ---GAGG.C....GCT--TC...GCTA-AC.AG......G...CTAA.A..C-CCGCGT.  2795
Pro. anacardii duprei  .TCG-----....GAC.T...CG.A..-GCGCG.TACG.G....CGT.G..C-AC.CGAC  2790
Kallimoides rumia      ...ACGAAGT.A.GAT.C.A.CG.A.A-...--.T.TGAACGAC.ACGACAC-A.G..GC  2785
Araschina levana       ...A..TTATACG----------------------------------AA..T-T..G.G.  2688
Junonia stygia         ...C..C.TCCTT------.CGTTSMK-.TC--.AYGW..G...AC.GG.AG-CG.T.G.  2783
A. jatrophae saturata  ...C..TTCG.C.------....G.AG-ATA--.AT.G.G..AAT--.G..T---.TGAC  2757
Mallika jacksoni       ...T..TCGT.TA------..CG.C..-...--.ATCG.GCGAC.--.-------GTA..  2768
Helicoverpa zea        TACG.AA.T.G.TGT.-TAC....C..T.........T........GT.ACA......  2827
Helicoverpa armigera   TACGCGA.T.G.TAT.-T.C....C..T.........T........GT.ACA......  2803
Chrysodeixis includens GC.C..TCA.GTTAG..ACGCGTT...G..A..............C.A.A---TA....  2646
Chrysodeixis eriosoma  GC.--TCTCG.CAAG..ACGCGTT...G..A..............C.A.A---TA....  2661
Chrysodeixis chalcites GC.--TCTCG.CAAG..AAGCGTT...G..A..............C.A.A---TA....  2662
Coeliades ramanatek    ---------------.TAT.....AATGCGCTAA.G-------.G.--CGAACG.CA.G  2909
```

```
Meroptera pravella     ---------------T.G....C.CACG.C....A.--------CC.A-.GCGAC.CTTC  2778
Salamis anteva         A.---.-.-.A..GAC.A.ACA.G..GTACG.T.---------------.CATCT...AC  2790
Precis andremiaja      ..CG..-.-CA..GGT.C.A.CGCGT.T..C.....C-------TCACG.GGTC..CATC  2786

Consensus              GAGA---T----AYGCGTCTAGDCGTGCTTMTATCGTGT----------------NGAKT  2784
Papilio xuthus         C.C.--------CC.----------------..---.A.------------GTTGT.CG.  2816
Pro. anacardii duprei  .C..--------CA...GTAC.G..A-------------------------CATCGTGTA  2817
Kallimoides rumia      .CC.--------CCA.T.T.C.AG.ACACACC.CACACG------------GACGACGTC  2825
Araschina levana       A.T.--------.ATGCGTAT.TATCAA..G..CGTGTG------------ACGACATAA  2728
Junonia stygia         TGCG--------.C.GTG.GC.AAAGCGCGCG..C..---------------AC.CTA  2820
A. jatrophae saturata  T.T.--------TC.TTCGAT.A.A.CTC.CG.GAT...------------GTCGTT.T.  2797
Mallika jacksoni       T.TC--------GTATAA.GTATTTGCG..CGT.ATC.G------------TGTAGA.TC  2808
Helicoverpa zea        ...GGGG.TGCG.T....G...C....T..A.C.T....CGTCCGT-AAAGCGTGA..CG  2886
Helicoverpa armigera   ...GGGG.TGCG.T....G...C....T..A.C.T....CGTCCGCTTTAGCGTGA..CG  2863
Chrysodeixis includens .C.GGGG.CGAG.GC--.....GTC...G.A......A.TCGACATAA--T---------  2693
Chrysodeixis eriosoma  ....GGG.CGAG.AC--..A..GTC..AA.A......A.ATCGACATAATA---------  2710
Chrysodeixis chalcites ....GGG.CGAG.AC--..A..GTC..AA.G......A.ATCGACATAATA---------  2711
Coeliades ramanatek    AG..AGC.TTCGCT.A..T.C.CGTC..AGCGTAT....----------------C.TG.  2953
Meroptera pravella     .GACTAC.GTCATT....ACG.TATAC.AGAG------------------------.G.  2813
Salamis anteva         .CATCTC.CACC.CA..CACGCA..CA.GCACGCACGCA----------------C.CGC  2834
Precis andremiaja      ...TGTG.ATAACGA..--...T...A..GCGGA..C.C----------------TACG.  2828

Consensus              STAKATAKWKGACAGTGTCTGTKTAYGTTTAAACRWTYAGTAGGCGGACTCGACGTCCGA  2844
Papilio xuthus         G.GTG.GTGT.TGT....G...G..T..G...TATT.A......................  2876
Pro. anacardii duprei  CACGC.CGCGCT.GCATCGCTCGATAT.A.....GAGT......................  2877
Kallimoides rumia      G.CG.ACGAGA..GA..GAA.CGCGC.CGCG.G.GCGCG.....................  2885
Araschina levana       AG.C..CGTAT..TC.AA.GCGT.TC..GGC.GAGTCA......................  2788
Junonia stygia         CGGGTACGGT....TC..AGACGCGCA.CG...AAAAT......................  2880
A. jatrophae saturata  A..T...TATT.ATTAA.TAA.A..C..A..T.ACGGCC.....................  2857
Mallika jacksoni       GA.T.A.TAAT.ACT.CGT.TAT..ATA...TT.GAAA......................  2868
Helicoverpa zea        CC.GCG.ATGC.A.C.CA..TGCG.GTGCA.CG.CA.T......................  2946
Helicoverpa armigera   CC.GCG.ATGC.A.C.CA..TGCGTGCAACG.C.AA.C......................  2923
Chrysodeixis includens ------------.GTGTC.GC.A..TCG....GGGTCG......................  2741
Chrysodeixis eriosoma  ------------.C.....C.ATA.TCG...G.GAC.T......................  2758
Chrysodeixis chalcites ------------.C.....C.ATA.TCG...G.GAC.T......................  2759
Coeliades ramanatek    TATAGA.AAAT.T.TAAATATATATACAA..T.TATAC......................  3013
Meroptera pravella     GAGTTCGGTC...C..CGAC.GTGGGT..CTCT.CG.GC.....................  2873
Salamis anteva         T.GTG.GTTT.TGT....G...G.GT..GGCGTTTTAG......................  2894
Precis andremiaja      A.TA..TTAC.T...C.CAGC.G..C.ACG.CGACGAC......................  2888

Consensus              AGAGCGCGTCGACGCCGACGTCGTAACGGGGCGT-N---------------------GA  2881
Papilio xuthus         .......A...GT..GACGT.GC.GCT.TTTGC.TT---------------------TT  2914
Pro. anacardii duprei  ...................C..G.......-------------------------..  2911
Kallimoides rumia      ...................C..G.......-------------------------..  2919
Araschina levana       ......................G....ATGAAAA---------------------AT  2826
Junonia stygia         ...................C..G.......-------------------------..  2914
A. jatrophae saturata  ...................C..A......TT.A-----------------------..  2893
Mallika jacksoni       ..T................G..A.......-------------------------..  2902
Helicoverpa zea        .......A.........T......TCTC.TT...GTTCA---------------CGCACG  2991
Helicoverpa armigera   .......A.........T......TCTC.TT...GTTCA---------------CGCACG  2968
Chrysodeixis includens .......A.........TA.....TCGT.C.A..CTAGCCGCGTTCAACGCG---TTTT.  2798
Chrysodeixis eriosoma  .......A.........TA.....TCAT.C.A..CTAGCCGCGTTCTGAACGCGTAACT.  2818
Chrysodeixis chalcites .......A.........TA.....TCAT.C.A..CTAGCCGCGTTCTGAACGCGTAACT.  2819
Coeliades ramanatek    ..T...T..........T..C...CGTC.T.ACACACGCGAGTGTGTT-----------C  3062
Meroptera pravella     ......TA......T..C.T...ACG..TC.T..GTGTGTGTAATGCA-----------C  2922
Salamis anteva         ...................C..G-.AC...GCG-------------------------.  2928
```

```
Precis andremiaja         ....................C..GCGAC...GAG-------------------------.  2923

Consensus                 CTCGTCTCGGCGAAAGCGGCGCCGTCGCGCTGACGGATATCGCGTCTGCC-CGTTTTTTT  2940
Papilio xuthus            TG.TG.GGT.GCGGC.AA...T...T.A......................---.CGA...  2971
Pro. anacardii duprei     .C............................C...................T.....-...  2970
Kallimoides rumia         .C....C......................TC...................T.....-...  2978
Araschina levana          .........A...................T....................T....--...  2884
Junonia stygia            .C...........................TC...................T.....-...  2973
A. jatrophae saturata     .C.......A...................TC...................T.....-...  2952
Mallika jacksoni          .C..CT...A.....T..............C...................T.........  2962
Helicoverpa zea           G.ACG.AGTT...TT...T..T...AA......................--.TCA.....  3049
Helicoverpa armigera      G.ACG.AGTT...TT...T..T...AA......................--.TCA.....  3026
Chrysodeixis includens    T..TCG.GTC...TT...T..T...AA......................--.TCA.....  2856
Chrysodeixis eriosoma     T..TCG.GTC...TT...T..T...AA......................--.TCA.....  2876
Chrysodeixis chalcites    T..TCG.GTC...TT...T..T...AA......................--.TCA.....  2877
Coeliades ramanatek       GA..AT.AC.GTTGTA.....T...AT..................--CTG-.C.CGA...  3119
Meroptera pravella        AA.ACGCGCT..TTT...C......A................A..--CTG-.C.C.CA..  2979
Salamis anteva            .............................TC...................-TCG......  2987
Precis andremiaja         .C.....................T.....TC...................-TCG..C...  2982

Consensus                 TTATCGTTGGCCTCAGATCAGGGAGGATCACCCGCCGAATTTAAGCATATTAGTAAGCGG  3000
Papilio xuthus            ............................................................  3031
Pro. anacardii duprei     ............................................................  3030
Kallimoides rumia         ............................................................  3038
Araschina levana          ............................................................  2944
Junonia stygia            ............................................................  3033
A. jatrophae saturata     ............................................................  3012
Mallika jacksoni          ............................................................  3022
Helicoverpa zea           ............................................................  3109
Helicoverpa armigera      ............................................................  3086
Chrysodeixis includens    ............................................................  2916
Chrysodeixis eriosoma     ............................................................  2936
Chrysodeixis chalcites    ............................................................  2937
Coeliades ramanatek       ............................................................  3179
Meroptera pravella        ............................................................  3039
Salamis anteva            ............................................................  3047
Precis andremiaja         ............................................................  3042

Consensus                 AGGAAAAGAAACTAACCAGGATTTCCTTAGTAGCGGCGAGCGAACAGGAAAG-AGCCCAG  3059
Papilio xuthus            ................A.................................TT-.......  3090
Pro. anacardii duprei     ...................................................C-.......  3089
Kallimoides rumia         ..........................C........................C-.......  3097
Araschina levana          ...................................................T-.......  3003
Junonia stygia            ...................................................C-.......  3092
A. jatrophae saturata     ..................................................T.-.......  3071
Mallika jacksoni          ...................................................C-.......  3081
Helicoverpa zea           ..................................................T.A.......  3169
Helicoverpa armigera      ..................................................T.A.......  3146
Chrysodeixis includens    ....................................................A.......  2976
Chrysodeixis eriosoma     ....................................................A.......  2996
Chrysodeixis chalcites    ....................................................A.......  2997
Coeliades ramanatek       ................A..................................T-.......  3238
Meroptera pravella        ................T.................................T.A.......  3099
Salamis anteva            ...................................................C-.......  3106
Precis andremiaja         ..........................C.......................CA-.......  3101
```

```
Consensus               CACTGAATCCCGCGGTTGTAACGATCGCGGGAGATGTGGTGTTCGGGAGGTWCCGCTTTC  3119
Papilio xuthus          ................C...T...A..........................T........  3150
Pro. anacardii duprei   ...................................................AT.......  3149
Kallimoides rumia       ...................................................AT.......  3157
Araschina levana        ...................................................AT.......  3063
Junonia stygia          ...................................................A........  3152
A. jatrophae saturata   ...................................................AT.......  3131
Mallika jacksoni        ...................................................AT.......  3141
Helicoverpa zea         .............C.....TCA.GCG.........................T........  3229
Helicoverpa armigera    .............C.....TCA.GCG.........................T........  3206
Chrysodeixis includens  .............C.....TCA.GCG.........................T........  3036
Chrysodeixis eriosoma   .............C.....TCA.GCG.........................T........  3056
Chrysodeixis chalcites  .............C.....TCA.GCG.........................T........  3057
Coeliades ramanatek     .............TT.C..CT....T.........................T........  3298
Meroptera pravella      .G.C............C..C...G........C..................T........  3159
Salamis anteva          ...................................................AT.......  3166
Precis andremiaja       .................C.C...............................AT.......  3161

Consensus               TCGTCGTCGCCACTCCTGTCCAAGTTCGTCTTGAACGGGGCCGTTTTCCCGTAGAGGGTG  3179
Papilio xuthus          .........AT.................................................  3210
Pro. anacardii duprei   ............................................................  3209
Kallimoides rumia       ............................................................  3217
Araschina levana        ............................................................  3123
Junonia stygia          ............................................................  3212
A. jatrophae saturata   ............................................................  3191
Mallika jacksoni        ...........G................................................  3201
Helicoverpa zea         ......CG.T.G.......................................C........  3289
Helicoverpa armigera    ......CG.T.G.......................................C........  3266
Chrysodeixis includens  ......CGAT.G................................................  3096
Chrysodeixis eriosoma   ......CGAT.G................................................  3116
Chrysodeixis chalcites  ......CGAT.G................................................  3117
Coeliades ramanatek     ...CG....T..................................................  3358
Meroptera pravella      .........AG.................................................  3219
Salamis anteva          ............................................................  3226
Precis andremiaja       ............................................................  3221

Consensus               CCAGGCCCGTAGCGACGGAGCGAGACGGCGAGAGGKACTCTCCTCAGAGTCGGGTTGCTT  3239
Papilio xuthus          ....................G.C.G..........G..A.....A...............  3270
Pro. anacardii duprei   ...................................T........................  3269
Kallimoides rumia       ...................................T..G.....................  3277
Araschina levana        ...................................T..C.....................  3183
Junonia stygia          ...................................T........................  3272
A. jatrophae saturata   ...................................T..A.....................  3251
Mallika jacksoni        ...................................T..A.....................  3261
Helicoverpa zea         ....................GACCGT.........G........T...............  3349
Helicoverpa armigera    ....................GACCGT.........G........T...............  3326
Chrysodeixis includens  ....................GATTG..........G........T...............  3156
Chrysodeixis eriosoma   ....................GATTG..........G........T...............  3176
Chrysodeixis chalcites  ....................GATTG..........G........T...............  3177
Coeliades ramanatek     ......................C..TC........G........................  3418
Meroptera pravella      ............T.......GTC.G..........G........................  3279
Salamis anteva          ...................................T........................  3286
Precis andremiaja       ...................................T........................  3281

Consensus               GAGAGTGCAGCCCTAAGTGGGTGGTAAACTCCATCTAAGGCTAAATATTACCGCGAGACC  3299
Papilio xuthus          ............................................................  3330
```

```
Pro. anacardii duprei     ............................................................  3329
Kallimoides rumia         ............................................................  3337
Araschina levana          ............................................................  3243
Junonia stygia            ............................................................  3332
A. jatrophae saturata     ............................................................  3311
Mallika jacksoni          ............................................................  3321
Helicoverpa zea           ............................................................  3409
Helicoverpa armigera      ............................................................  3386
Chrysodeixis includens    ............................................................  3216
Chrysodeixis eriosoma     ............................................................  3236
Chrysodeixis chalcites    ............................................................  3237
Coeliades ramanatek       ............................................................  3478
Meroptera pravella        ............................................................  3339
Salamis anteva            ............................................................  3346
Precis andremiaja         .................C..........................................  3341

Consensus                 GATAGCGAACAAGTACCGTGAGGGAAAGTTGAAAAGAACTTTGAAGAGAGAGTTCAAGAG  3359
Papilio xuthus            ............................................................  3390
Pro. anacardii duprei     ............................................................  3389
Kallimoides rumia         ............................................................  3397
Araschina levana          ............................................................  3303
Junonia stygia            ............................................................  3392
A. jatrophae saturata     ............................................................  3371
Mallika jacksoni          ............................................................  3381
Helicoverpa zea           ............................................................  3469
Helicoverpa armigera      ............................................................  3446
Chrysodeixis includens    ............................................................  3276
Chrysodeixis eriosoma     ............................................................  3296
Chrysodeixis chalcites    ............................................................  3297
Coeliades ramanatek       ............................................................  3538
Meroptera pravella        ............................................................  3399
Salamis anteva            ............................................................  3406
Precis andremiaja         ............................................................  3401

Consensus                 TACGTGAAACCGTTCAGGGGTAAACCTGCGAAACTCGAATGAACGAACGGAGAGATTCAT  3419
Papilio xuthus            ............................................................  3450
Pro. anacardii duprei     ............................................................  3449
Kallimoides rumia         ............................................................  3457
Araschina levana          ............................................................  3363
Junonia stygia            ............................................................  3452
A. jatrophae saturata     ............................................................  3431
Mallika jacksoni          ............................................................  3441
Helicoverpa zea           ............................................................  3529
Helicoverpa armigera      ............................................................  3506
Chrysodeixis includens    ............................................................  3336
Chrysodeixis eriosoma     ............................................................  3356
Chrysodeixis chalcites    ............................................................  3357
Coeliades ramanatek       ............................................................  3598
Meroptera pravella        ............................................................  3459
Salamis anteva            ............................................................  3466
Precis andremiaja         ............................................................  3461

Consensus                 CGTCATTCSACGGCGTACGGGCGCGCGCCTCGATGTCGCASACCTCCCCTCG---GGGNG  3476
Papilio xuthus            ........CG........TCTAT.A...TCT....AG..GTC.G....TGTT--AAA.G.  3508
Pro. anacardii duprei     .....C..G...............................CG..........--C...T.  3507
Kallimoides rumia         ........G...............................C...........--C...T.  3515
```

```
Araschina levana          ........G................T...........T..T...........--T...T.  3421
Junonia stygia            ........G...............................CT..........--C...T.  3510
A. jatrophae saturata     ........G...............................C...........--C...T.  3489
Mallika jacksoni          ........G...............................C...........--C...T.  3499
Helicoverpa zea           ........CT..........A..................CGGTT..GATCG.--------  3581
Helicoverpa armigera      ........CT..........A..................CGGTT..GATCG.--------  3558
Chrysodeixis includens    ........CT..........A..................CG.T...GATCG.--------  3388
Chrysodeixis eriosoma     ........CC..........A..................CG.T...GATCG.--------  3408
Chrysodeixis chalcites    ........CC..........A..................CG.T...GATCG.--------  3409
Coeliades ramanatek       ........CG.........T..................TCGGTT.T--------------  3644
Meroptera pravella        ...T....CT.....C..TT......T..CGATGTGTT.GGTTTCGAT.-----------  3508
Salamis anteva            ........G...............................C...........--C...T.  3524
Precis andremiaja         ........G..............................GC..TCG...CTCGCG..TGC  3521

Consensus                 -G----GNGCGGCACGGGTCGCGTCCGTCGACGTCCGTSGACGGCGTGCACTTCTCTCTY  3531
Papilio xuthus            GATAGC.G..TT......C..ATCGA........T..CG....................T  3568
Pro. anacardii duprei     C.GGTG.T.........................C....C....................C  3567
Kallimoides rumia         C.GGTC.C..............................C....................C  3575
Araschina levana          T.--TC.T.G...........T............T...C....................C  3479
Junonia stygia            C.GGTA.C..............................C....................C  3570
A. jatrophae saturata     C.GTTC.T..............................C....................C  3549
Mallika jacksoni          C.TT--.C..............................C....................C  3557
Helicoverpa zea           --------TA...........................CG....................T  3633
Helicoverpa armigera      --------TA...........................CG....................T  3610
Chrysodeixis includens    --------C............................AG.......C............T  3440
Chrysodeixis eriosoma     --------CT...........................AG....................T  3460
Chrysodeixis chalcites    --------CT...........................AG....................T  3461
Coeliades ramanatek       -TAATC.T...A...........CA............CG....................T  3703
Meroptera pravella        --------.T.CGCAC...GCGTGT..GTAG......GG....................T  3560
Salamis anteva            C.GGCC.T..............................C....................C  3584
Precis andremiaja         GTTTAA.T..............................C....................C  3581

Consensus                 AGT-AATACATCGCGACCCGTTCGATGTCGGTCTAAGCGCCGTCCGGGAGTCCAGTCGTC  3590
Papilio xuthus            ...-...G...................................T......C...T.....  3627
Pro. anacardii duprei     ...-......................................C...............C.  3626
Kallimoides rumia         ...-........................................................  3634
Araschina levana          ...-.......................................T...A........T..T  3538
Junonia stygia            ...-.......................................................A  3629
A. jatrophae saturata     ...-.......................................T................  3608
Mallika jacksoni          ...-....................................................A...  3616
Helicoverpa zea           ...A.......................................A......C..C..T.C.  3693
Helicoverpa armigera      ...A.......................................A......C..C..T.C.  3670
Chrysodeixis includens    ...A.......................................A......C..C..T.C.  3500
Chrysodeixis eriosoma     ...A.......................................A......C..C..T.C.  3520
Chrysodeixis chalcites    ...A.......................................A......C..C..T.C.  3521
Coeliades ramanatek       ...-....................GA............TT.....T.......C......  3762
Meroptera pravella        ...-.C.G.......................C..........CTT.....C...T.....  3619
Salamis anteva            ...-.................................................R.Y....  3643
Precis andremiaja         ...-.................................................GCSYRG.  3640

Consensus                 CCTTTCGCGGGGGGCTAGTRGCSCGWGANNN-CG--------GRCCGACCG----TCGGA  3637
Papilio xuthus            A.CCCGTTAAAA..G..ACG.TTG.ACCGTGA..GTTTTTTGTG.......GCCG.....  3687
Pro. anacardii duprei     GTG......CAC.....T.G.AC..T.G----..G------T.G.......GCCG.....  3676
Kallimoides rumia         GTG......CAC.....T.G.AC..T..----..G------T.G.......GCCG.....  3684
Araschina levana          GTG...A..C.CTA..GC.G.AC..T..----..G------T.G.......GCCG.....  3588
Junonia stygia            GTG......CACT....T.G.AC..T..----..G------T.G.......GCCG.....  3679
```

```
A. jatrophae saturata  GTG......CAC.....T.G.AC..T..----..G------T.G.......GCCG.....  3658
Mallika jacksoni       TTG......CAC.....T.G.AC..T..----..G------T.G.......GCCG.....  3666
Helicoverpa zea        ....CACG....T.A.G.GAC.G..ACGGTGG.C--------.A...G...----.....  3741
Helicoverpa armigera   ....CACG....T.A.G.GAC.G..ACGGTGG.C--------.A...G...----.....  3718
Chrysodeixis includens ....CACG....TAG.G.GAC.G..ACGGTGG.C--------.A...G...----.....  3548
Chrysodeixis eriosoma  ....CACG....TAG.G.GAC.G..ACGGTGG.C--------.A...G...----.....  3568
Chrysodeixis chalcites ....CACG....TAG.G.GAC.G..ACGGTGG.C--------.A...G...----.....  3569
Coeliades ramanatek    .AACGAAA..C..A.G.T.G.ACA.C..CGTT..--------CCGACCGGCTATAC....  3814
Meroptera pravella     ...C...G....C.GGT.GAC.TT.C.GTGGC..--------.C.G.CTG-----.....  3666
Salamis anteva         GGA----...TA.TAC..AAT.GTAC..ATTTTA--------TA.GA.------------  3679
Precis andremiaja      SG.SGGA...TA.TAC.AAAT.GTAC..A--TAA--------TA.GA.------------  3678

Consensus              CGGTANNA----------CWKTT-ATACGAATCGCGCACGCGCCTCRCGCGCGTCCGGCC  3686
Papilio xuthus         .....GA.G---------------.C.............--.GCATA............  3730
Pro. anacardii duprei  .....GT.CAAAATCGTA.TTCA-....................G............  3735
Kallimoides rumia      .....GT.CAAAATCGTA.TTCA-...................AG............  3743
Araschina levana       .....GT.CAAAATCGTA.TAA.A.................T...A............  3648
Junonia stygia         .....GT.CAAAATCGTA.TTAAA....................G............  3739
A. jatrophae saturata  .....GT.CAAAATCGTA.TTCA-.................T...A............  3717
Mallika jacksoni       .....GT.CAAAATCGTA.TTAA-...................AG............  3725
Helicoverpa zea        ...---------------TAG..CTG...............TT.ATGA...........  3786
Helicoverpa armigera   ...---------------TAG..CTG...............TT.ATGA...........  3763
Chrysodeixis includens ...---------------TAG..CTG...............TT.TAGA...........  3593
Chrysodeixis eriosoma  ...---------------TAG..CTG...............TT.TAGA...........  3613
Chrysodeixis chalcites ...---------------TAG..CTG...............TT.TAGA...........  3614
Coeliades ramanatek    .....TTCA---------AACA.G...G........-ACGCGT..ATA...........  3864
Meroptera pravella     .....TA.--------------------CTG.GAAC.G...A.GCTTTAA....T.....  3706
Salamis anteva         ------------------------------..............G............  3708
Precis andremiaja      ------------------------------..............G............  3707

Consensus              CGACGCAAGCGAACGTCGTANTAWGTCGATGTCCTGCCCGAGTGCGGACGTNGGTGCGGC  3746
Papilio xuthus         .........AT----AACGTCGTTA....G........AT........A.C........  3786
Pro. anacardii duprei  ...........G..C....CC.A..........................C.AC.....  3795
Kallimoides rumia      ..............C....TC.A...............CCGT----------------  3787
Araschina levana       ................T..CGT---..................----------------  3689
Junonia stygia         ..............C....TC.A.................C.----------------  3783
A. jatrophae saturata  ..............C....TA.A.................CGT----------------  3761
Mallika jacksoni       ..............C....TA.A....C..............----------------  3769
Helicoverpa zea        .........TC.......---..TCCAACGTCTAC...TC...........A........  3843
Helicoverpa armigera   .........TC.......---..TCCAACGTCTAC...TC...........A........  3820
Chrysodeixis includens .........TC.......---..TCCAACGTCTAC...AC...........A........  3650
Chrysodeixis eriosoma  .........TC.......---..TCCAACGTCTAC...AC...........A........  3670
Chrysodeixis chalcites .........TC.......---..TCCAACGTCTAC...AC...........A........  3671
Coeliades ramanatek    ..----.C..A.GACAACGTCGTCA....C........TAT..........T........  3920
Meroptera pravella     ....----.A.GGT.GTGTAGTC.....A........TA..C......T.T..C..T..  3762
Salamis anteva         ..............C....TC.C...........................C.AC.....  3768
Precis andremiaja      ..............----GCAACA.................C........C.AC.....  3763

Consensus              GCGTCTGTNGTCGCNGCCGTGCTGTCTCGGACTGTGCG-CGTCTCCGTCTGCGATGATTC  3805
Papilio xuthus         ..A.....T.....T.......A.........C.....CG-....A.............  3845
Pro. anacardii duprei  ..TG....C.....A.................C.....-...A................  3854
Kallimoides rumia      --------------------..................CG-.A................  3826
Araschina levana       --------------------CGGA-.GTC....AG...CT.CTGT..............  3728
Junonia stygia         --------------------CGGACG-TC...KSK...CKKMTSN....K.........  3822
A. jatrophae saturata  --------------------.T................CGTATCT..............  3801
Mallika jacksoni       --------------------CGGA-.ATC...GCG...CT.CTGT....GCA.CC.....  3808
```

```
Helicoverpa zea            .......CT.....C.......................-......T..............  3902
Helicoverpa armigera       .......CT.....C.......................-......T..............  3879
Chrysodeixis includens     .......CA.....C......T....A...........-......T..............  3709
Chrysodeixis eriosoma      ........C.....A......T....A...........-......T..............  3729
Chrysodeixis chalcites     ........C.....A......T....A...........-......T..............  3730
Coeliades ramanatek        ........T.....C.......A.........A.....-......T..............  3979
Meroptera pravella         ...C....T.....T.--CC.TG..T...A........-...T..G..............  3819
Salamis anteva             ..TG....C.....A......TA...............-...A.................  3827
Precis andremiaja          .TTG....C.....A.................C.....-...A.................  3822

Consensus                  AKTTTCGGGCACTCGCAGGACCCGTCTTGAAACACGGACCAAGGAGTCTAGCATGTGTGC  3865
Papilio xuthus             .G...........................T..............................  3905
Pro. anacardii duprei      .T..........................................................  3914
Kallimoides rumia          .T..........................................................  3886
Araschina levana           .T..........................................................  3788
Junonia stygia             .T.......-------MRSW.S.RK.W.................................  3875
A. jatrophae saturata      .T..........................................................  3861
Mallika jacksoni           .T..........................................................  3868
Helicoverpa zea            .G......................................................A...  3962
Helicoverpa armigera       .G......................................................A...  3939
Chrysodeixis includens     .G......................................................A...  3769
Chrysodeixis eriosoma      .G......................................................A...  3789
Chrysodeixis chalcites     .G......................................................A...  3790
Coeliades ramanatek        .G..........................................................  4039
Meroptera pravella         .G..........................................................  3879
Salamis anteva             .T...........................................KKYKW..RW..CATT  3887
Precis andremiaja          .T............................................KYKM.MG.CAT-TG  3881

Consensus                  GAGTCATTGAGATA-----N-ATAAAACTGAAAGGCGCAACGAAAGTGAAGGCGCGCGCT  3919
Papilio xuthus             ...........T..TGTTAAA.-----..........T......................  3960
Pro. anacardii duprei      .............TTTTATAC.......................................  3974
Kallimoides rumia          .............TTTTATAC.......................................  3946
Araschina levana           ..............TTTATAC.......................................  3848
Junonia stygia             .............TTTTATAC..G....................................  3935
A. jatrophae saturata      ..............TTTATAC.......................................  3921
Mallika jacksoni           .............TATTTTAC.......................................  3928
Helicoverpa zea            ..............-------..T....................................  4015
Helicoverpa armigera       ..............-------..T....................................  3992
Chrysodeixis includens     ..............-------.......................................  3822
Chrysodeixis eriosoma      ..............-------.......................................  3842
Chrysodeixis chalcites     ..............-------.......................................  3843
Coeliades ramanatek        T...........CTAT---AC.................................T.....  4096
Meroptera pravella         .............--------..C............A.......................  3931
Salamis anteva             ...ATT..TTA.C--------..G....................................  3939
Precis andremiaja          AGA.TCA.TTA.C--------.CG....................................  3933

Consensus                  ARAC--CCGCGCGCTCAGGGAGGATGGAGCGTCGGTCTCGATCGATCTCTCGCACTCCCG  3977
Papilio xuthus             CGT.GCG...T.AGGG..---.ATG.ATAT..ATA.T.AT..AT..A.............  4017
Pro. anacardii duprei      .A..--......................................................  4032
Kallimoides rumia          .A..--...........................................C..........  4004
Araschina levana           TA..ACA......................AT.............................  3908
Junonia stygia             .A..--A.....................................................  3993
A. jatrophae saturata      .A..--......................................................  3979
Mallika jacksoni           .A..--............................A.........................  3986
Helicoverpa zea            TG----............................A...A.G...................  4071
Helicoverpa armigera       TG----............................A...A.G...................  4048
```

```
Chrysodeixis includens .G----.....T......................A...A.G...................  3878
Chrysodeixis eriosoma  .G----.....T......................A...A.G...................  3898
Chrysodeixis chalcites .G----.....T......................A...A.G...................  3899
Coeliades ramanatek    TG----T...............................T.....................  4152
Meroptera pravella     TG----T....T...................C........G...GC..............  3987
Salamis anteva         .A..--......................................................  3997
Precis andremiaja      .A..--A..........................................C..........  3991

Consensus              AGGCGTCTCGTTTCCAATCCGTGAATGCAGGCGCGCTCTGAGCACAGATGCTGGGACCCG  4037
Papilio xuthus         ..................TA.......T................G...............  4077
Pro. anacardii duprei  ............................................................  4092
Kallimoides rumia      ...........................................R................  4064
Araschina levana       ............................................................  3968
Junonia stygia         ............................................................  4053
A. jatrophae saturata  ............................................................  4039
Mallika jacksoni       ............................................................  4046
Helicoverpa zea        ............................................T.A.............  4131
Helicoverpa armigera   ............................................T.A.............  4108
Chrysodeixis includens ............................................T.A.............  3938
Chrysodeixis eriosoma  ............................................T.A.............  3958
Chrysodeixis chalcites ............................................T.A.............  3959
Coeliades ramanatek    ............................................................  4212
Meroptera pravella     ...........................T...............G................  4047
Salamis anteva         ............................................................  4057
Precis andremiaja      ............................................................  4051

Consensus              AAAGATGGTGAACTATGCCTGGTCAGGTCGAAGTCAGGGGAAACCCTGATGGAGGACCGT  4097
Papilio xuthus         ............................................................  4137
Pro. anacardii duprei  ............................................................  4152
Kallimoides rumia      ............................................................  4124
Araschina levana       ............................................................  4028
Junonia stygia         ............................................................  4113
A. jatrophae saturata  ............................................................  4099
Mallika jacksoni       ............................................................  4106
Helicoverpa zea        ............................................................  4191
Helicoverpa armigera   ............................................................  4168
Chrysodeixis includens ............................................................  3998
Chrysodeixis eriosoma  ............................................................  4018
Chrysodeixis chalcites ............................................................  4019
Coeliades ramanatek    ............................................................  4272
Meroptera pravella     ............................................................  4107
Salamis anteva         ............................................................  4117
Precis andremiaja      ............................................................  4111

Consensus              AGCGATTCTGACGTGCAAATCGATCGTCGGAACTGGGTATAGGGGCGAAAGACTAATCGA  4157
Papilio xuthus         ............................................................  4197
Pro. anacardii duprei  ............................................................  4212
Kallimoides rumia      ............................................................  4184
Araschina levana       ............................................................  4088
Junonia stygia         ............................................................  4173
A. jatrophae saturata  ............................................................  4159
Mallika jacksoni       ............................................................  4166
Helicoverpa zea        ............................................................  4251
Helicoverpa armigera   ............................................................  4228
Chrysodeixis includens ............................................................  4058
Chrysodeixis eriosoma  ............................................................  4078
```

```
Chrysodeixis chalcites ............................................................  4079
Coeliades ramanatek    ............................................................  4332
Meroptera pravella     ............................................................  4167
Salamis anteva         ............................................................  4177
Precis andremiaja      ............................................................  4171

Consensus              ACCATCTAGTAGCTGGTTCCGTCCGAAGTTTCCCTCAGGATAGCTGGCGTCGATTATNAA  4217
Papilio xuthus         .....................................................A..CG..  4257
Pro. anacardii duprei  ......................................................A..C..  4272
Kallimoides rumia      .........................................................T..  4244
Araschina levana       .........................................................T..  4148
Junonia stygia         .........................................................T..  4233
A. jatrophae saturata  .........................................................T..  4219
Mallika jacksoni       .........................................................T..  4226
Helicoverpa zea        .......................................................TG-..  4310
Helicoverpa armigera   .......................................................TG-..  4287
Chrysodeixis includens .......................................................TG-..  4117
Chrysodeixis eriosoma  .......................................................TG-..  4137
Chrysodeixis chalcites .......................................................TG-..  4138
Coeliades ramanatek    .......................................................TG-G.  4391
Meroptera pravella     .......................................................CG-..  4226
Salamis anteva         .........................................................T..  4237
Precis andremiaja      .........................................................T..  4231

Consensus              CAGTCYCATCCGGTAAAGCGAATGATTAGAGGCATTGGGGCCGAAACGACCTCAACCTAT  4277
Papilio xuthus         .....T......................................................  4317
Pro. anacardii duprei  .....C......................................................  4332
Kallimoides rumia      .....C......................................................  4304
Araschina levana       .....C......................................................  4208
Junonia stygia         .....C......................................................  4293
A. jatrophae saturata  .....C......................................................  4279
Mallika jacksoni       .....C......................................................  4286
Helicoverpa zea        .....T......................................................  4370
Helicoverpa armigera   .....T......................................................  4347
Chrysodeixis includens .....T......................................................  4177
Chrysodeixis eriosoma  .....T......................................................  4197
Chrysodeixis chalcites .....T......................................................  4198
Coeliades ramanatek    .....T......................................................  4451
Meroptera pravella     .....T......................................................  4286
Salamis anteva         .....C......................................................  4297
Precis andremiaja      .....C......................................................  4291

Consensus              TCTCAAACTTTAAATGGGTGAGAACTCCGGCTTACTCGAACGATGAAGCCGGAGATCTGA  4337
Papilio xuthus         ........................................T...................  4377
Pro. anacardii duprei  ............................................................  4392
Kallimoides rumia      ............................................................  4364
Araschina levana       ............................................................  4268
Junonia stygia         ............................................................  4353
A. jatrophae saturata  ............................................................  4339
Mallika jacksoni       ............................................................  4346
Helicoverpa zea        ......................T.....................................  4430
Helicoverpa armigera   ......................T.....................................  4407
Chrysodeixis includens ......................T.....................................  4237
Chrysodeixis eriosoma  ......................T.....................................  4257
Chrysodeixis chalcites ......................T.....................................  4258
Coeliades ramanatek    ..........................T........................A........  4511
```

```
Meroptera pravella        ........................................T...................  4346
Salamis anteva            ............................................................  4357
Precis andremiaja         ............................................................  4351

Consensus                 TGACGGTGCCAAGTGGGCCAATTTTGGTAAGCAGAACTGGCGCTGTGGGATGAACCAAAC  4397
Papilio xuthus            ............................................................  4437
Pro. anacardii duprei     ............................................................  4452
Kallimoides rumia         ............................................................  4424
Araschina levana          ............................................................  4328
Junonia stygia            ............................................................  4413
A. jatrophae saturata     ............................................................  4399
Mallika jacksoni          ............................................................  4406
Helicoverpa zea           ............................................................  4490
Helicoverpa armigera      ............................................................  4467
Chrysodeixis includens    ............................................................  4297
Chrysodeixis eriosoma     ............................................................  4317
Chrysodeixis chalcites    ............................................................  4318
Coeliades ramanatek       ............................................................  4571
Meroptera pravella        ............................................................  4406
Salamis anteva            ............................................................  4417
Precis andremiaja         ............................................................  4411

Consensus                 GTAGTGTTAAGGCGCCTAAAAAACGCTCATGGGACACCATGAAAGGCGTTGGTCGCTCAT  4457
Papilio xuthus            ............................................................  4497
Pro. anacardii duprei     ............................................................  4512
Kallimoides rumia         ............................................................  4484
Araschina levana          ............T...............................................  4388
Junonia stygia            ............................................................  4473
A. jatrophae saturata     ............................................................  4459
Mallika jacksoni          ............................................................  4466
Helicoverpa zea           ............................................................  4550
Helicoverpa armigera      ............................................................  4527
Chrysodeixis includens    ............................................................  4357
Chrysodeixis eriosoma     ............................................................  4377
Chrysodeixis chalcites    ............................................................  4378
Coeliades ramanatek       ............................................................  4631
Meroptera pravella        ............................................................  4466
Salamis anteva            ............................................................  4477
Precis andremiaja         ............................................................  4471

Consensus                 GACAGCAGGACGGTGGCCATGGAAGTCGGAATCCGCTAAGGAGTGTGCAACGACTCACCT  4517
Papilio xuthus            ............................................................  4557
Pro. anacardii duprei     ............................................................  4572
Kallimoides rumia         ................................T..............T..T.........  4544
Araschina levana          ............................................................  4448
Junonia stygia            ............................................................  4533
A. jatrophae saturata     ............................................................  4519
Mallika jacksoni          ............................................................  4526
Helicoverpa zea           ............................................................  4610
Helicoverpa armigera      ............................................................  4587
Chrysodeixis includens    ............................................................  4417
Chrysodeixis eriosoma     ............................................................  4437
Chrysodeixis chalcites    ............................................................  4438
Coeliades ramanatek       ............................................................  4691
Meroptera pravella        ............................................................  4526
Salamis anteva            ............................................................  4537
```

```
Precis andremiaja         ............................................................  4531

Consensus                 GCCGAAGCAACCAGCCCTGAAAATGGATGGCGCTGAAGCGTTTTGCCTATACACTACCGT  4577
Papilio xuthus            ............................................................  4617
Pro. anacardii duprei     ............................................................  4632
Kallimoides rumia         ............................................................  4604
Araschina levana          ............................................................  4508
Junonia stygia            ............................................................  4593
A. jatrophae saturata     ............................................................  4579
Mallika jacksoni          ............................................................  4586
Helicoverpa zea           ............................................................  4670
Helicoverpa armigera      ............................................................  4647
Chrysodeixis includens    ............................................................  4477
Chrysodeixis eriosoma     ............................................................  4497
Chrysodeixis chalcites    ............................................................  4498
Coeliades ramanatek       ............................................................  4751
Meroptera pravella        ............................................................  4586
Salamis anteva            ............................................................  4597
Precis andremiaja         ............................................................  4591

Consensus                 TACGGGCACGTGCGACGTTN---T-TACGTTTGCGTCATTATGCCGTAACGAGTAGGACG  4633
Papilio xuthus            ....T..CA.......TG.ATTT.A..TA.AAT...........................  4677
Pro. anacardii duprei     ..................-----.G...................................  4687
Kallimoides rumia         ..................-----.G...................................  4659
Araschina levana          ..................-----.G...................................  4563
Junonia stygia            ..................-----.G...................................  4648
A. jatrophae saturata     ..................-----.G...................................  4634
Mallika jacksoni          ..................-----.G...................................  4641
Helicoverpa zea           ........T..........CTT-.-----G.GA........A..................  4724
Helicoverpa armigera      ........T..........CTT-.-----G.GA........A..................  4701
Chrysodeixis includens    ........T..........CTT-.-----GYGA........A..................  4531
Chrysodeixis eriosoma     ........T..........CTT-.-----G.GA........A..................  4551
Chrysodeixis chalcites    ........T..........CTT-.-----G.GA........A..................  4552
Coeliades ramanatek       ........A.....TA..CTTT-.A.CG.A.GA...........................  4810
Meroptera pravella        ...C....T...A...TCGCC--CT.CG.GGC.T.......A..................  4644
Salamis anteva            ...................G-----...................................  4652
Precis andremiaja         ...................G-----...................................  4646

Consensus                 TGCGCGGCGGAGWGCGCAGAAGGGTCTGGGCGTGAGCCCGCTTGGAGCCTCCGTCGGTGC  4693
Papilio xuthus            ............A...................A........C..................  4737
Pro. anacardii duprei     ............T...............................................  4747
Kallimoides rumia         ............T...............................................  4719
Araschina levana          ............T...............................................  4623
Junonia stygia            ............T...............................................  4708
A. jatrophae saturata     ............T...............................................  4694
Mallika jacksoni          ............T...............................................  4701
Helicoverpa zea           ............A...............................................  4784
Helicoverpa armigera      ............A...............................................  4761
Chrysodeixis includens    ............A...............................................  4591
Chrysodeixis eriosoma     ............A...............................................  4611
Chrysodeixis chalcites    ............A...............................................  4612
Coeliades ramanatek       ..........T.A............................C.......G..........  4870
Meroptera pravella        ............A............................C..................  4704
Salamis anteva            ............T...............................................  4712
Precis andremiaja         ............T............................C..................  4706
```

```
Consensus                AGATCTTGGTGGTAGTAGCAAATACTCCAGCGAGGCCCTGGAGGACTGACGTGGAGAAGG  4753
Papilio xuthus           ............................................................  4797
Pro. anacardii duprei    ............................................................  4807
Kallimoides rumia        ............................................................  4779
Araschina levana         ............................................................  4683
Junonia stygia           ............................................................  4768
A. jatrophae saturata    ............................................................  4754
Mallika jacksoni         ............................................................  4761
Helicoverpa zea          ............................................................  4844
Helicoverpa armigera     ............................................................  4821
Chrysodeixis includens   ............................................................  4651
Chrysodeixis eriosoma    ............................................................  4671
Chrysodeixis chalcites   ............................................................  4672
Coeliades ramanatek      ............................................................  4930
Meroptera pravella       ............................................................  4764
Salamis anteva           ............................................................  4772
Precis andremiaja        ............................................................  4766

Consensus                GTTTCGCGTGAACAGTAGTTGCTCGCGAGTCAGTCGATCCTAAGCTCAAGGAGAGATCTT  4813
Papilio xuthus           ............................................................  4857
Pro. anacardii duprei    ............................................................  4867
Kallimoides rumia        ............................................................  4839
Araschina levana         ............................................................  4743
Junonia stygia           ............................................................  4828
A. jatrophae saturata    ............................................................  4814
Mallika jacksoni         ............................................................  4821
Helicoverpa zea          ......................................................A.....  4904
Helicoverpa armigera     ......................................................A.....  4881
Chrysodeixis includens   ......................................................A.....  4711
Chrysodeixis eriosoma    ......................................................A.....  4731
Chrysodeixis chalcites   ......................................................A.....  4732
Coeliades ramanatek      ............................................................  4990
Meroptera pravella       ......................................................A.....  4824
Salamis anteva           ............................................................  4832
Precis andremiaja        ...........................................................K  4826

Consensus                ATGTCGATGTGGCGTGTTTTTTTCN--------------------------------NTA  4841
Papilio xuthus           ...................A..ATTATTA-------------------TTTTT-ATTA..  4897
Pro. anacardii duprei    .......................TA---------------------------------..  4894
Kallimoides rumia        ..............GTA.GAA.AT----------------------------------A.  4865
Araschina levana         ....................C..TGTATA-------------------TTATATTATA..  4784
Junonia stygia           .......................G----------------------------------..  4854
A. jatrophae saturata    .......................-----------------------------------..  4839
Mallika jacksoni         ...................C...G----------------------------------..  4847
Helicoverpa zea          ........................AAAACGATATCATATACTTGTCTATGTGTGTTCGA.  4964
Helicoverpa armigera     ........................AAAACGATATCATATACTTGTCTATGTGTGTTCGA.  4941
Chrysodeixis includens   ........................AATGATGTATCGTTTTATT-------ATGGTATA..  4764
Chrysodeixis eriosoma    ........................AATAATGTATCATTTTATT-------ATGGTATA..  4784
Chrysodeixis chalcites   ........................AATAATGTATCGTTTTATW-------ATGGTATA..  4785
Coeliades ramanatek      ...................A...-----------------------------------A.  5015
Meroptera pravella       ...................A...T------------------------------------  4848
Salamis anteva           ...................C...GT--------------------------------A.T  4860
Precis andremiaja        W.TCTTT.A.ATT..C.C---GA.G--------------------------------A..  4851

Consensus                TTAATAAATAACGCCCTTTGAGCGAAAGGGAATCCGGTTCCTATTCCGGAACCCGGCAGC  4901
Papilio xuthus           AAG.........................................................  4957
```

```
Pro. anacardii duprei     ....CG......................................................  4954
Kallimoides rumia         .G.CC.......................................................  4925
Araschina levana          .A.C.........T....C.........................................  4844
Junonia stygia            .W.YN..W..WS................................................  4914
A. jatrophae saturata     .ATT..T.....................................................  4899
Mallika jacksoni          ..T..G......................................................  4907
Helicoverpa zea           .G-.........................................................  5023
Helicoverpa armigera      .GG.........................................................  5001
Chrysodeixis includens    A.G.........................................................  4824
Chrysodeixis eriosoma     A.G.........................................................  4844
Chrysodeixis chalcites    A.G.........................................................  4845
Coeliades ramanatek       .AT.........................................................  5075
Meroptera pravella        A.T.........................................................  4908
Salamis anteva            .A.NNNCG.GTMSSYS.K..........................................  4920
Precis andremiaja         ....CG......................................................  4911

Consensus                 GGAACCGTTTCAATAATCGTTCCCTCGTTTCTA--AAGCGAGTGTTCGACGGGGTAACCC  4959
Papilio xuthus            ..............................A..AAC........................  5017
Pro. anacardii duprei     ...............................A---.........................  5011
Kallimoides rumia         ...............................A---.........................  4982
Araschina levana          ............................G..A---.........................  4901
Junonia stygia            ...............................A---.........................  4971
A. jatrophae saturata     ...............................A---.........................  4956
Mallika jacksoni          ...............................A---.........................  4964
Helicoverpa zea           ..............................T..--C........................  5081
Helicoverpa armigera      ..............................T..--C........................  5059
Chrysodeixis includens    ..............................T..--C........................  4882
Chrysodeixis eriosoma     ..............................T..--C........................  4902
Chrysodeixis chalcites    ..............................T..--C........................  4903
Coeliades ramanatek       .............................A-..--.........................  5132
Meroptera pravella        ..............................-.T--.........................  4965
Salamis anteva            ..............................-C.--.........................  4977
Precis andremiaja         ..............................-C.--.........................  4968

Consensus                 AAAGTGGCCTGAAGACGCCGCCGAGGGGTCCGGGAAGAGTTTTCTTTTCTGCCTGAGCGT  5019
Papilio xuthus            ............................................................  5077
Pro. anacardii duprei     ............................................................  5071
Kallimoides rumia         ............................................................  5042
Araschina levana          ............................................................  4961
Junonia stygia            ............................................................  5031
A. jatrophae saturata     ............................................................  5016
Mallika jacksoni          ............................................................  5024
Helicoverpa zea           .........................A..................................  5141
Helicoverpa armigera      .........................A..................................  5119
Chrysodeixis includens    .........................A..................................  4942
Chrysodeixis eriosoma     .........................A..................................  4962
Chrysodeixis chalcites    .........................A..................................  4963
Coeliades ramanatek       ............................................................  5192
Meroptera pravella        .........................A..................................  5025
Salamis anteva            ............................................................  5037
Precis andremiaja         ............................................................  5028

Consensus                 TCGAGTTCCATGGAATCCTATAGAAGGGAGATATGGTTCGGAACGCGAAGAGCACCGCAT  5079
Papilio xuthus            ............................................................  5137
Pro. anacardii duprei     ............................................................  5131
Kallimoides rumia         ............................................................  5102
```

```
Araschina levana          ............................................................  5021
Junonia stygia            ............................................................  5091
A. jatrophae saturata     ............................................................  5076
Mallika jacksoni          ............................................................  5084
Helicoverpa zea           ............................................................  5201
Helicoverpa armigera      ............................................................  5179
Chrysodeixis includens    ............................................................  5002
Chrysodeixis eriosoma     ............................................................  5022
Chrysodeixis chalcites    ............................................................  5023
Coeliades ramanatek       ............................................................  5252
Meroptera pravella        ............................................................  5085
Salamis anteva            ............................................................  5097
Precis andremiaja         ............................................................  5088

Consensus                 TTGCGGCGGTGTCCGGATACTCTCTGCGGACCTTGAAAATTCAGGTGAGGGATGTACGTG  5139
Papilio xuthus            ............................................................  5197
Pro. anacardii duprei     ............................................................  5191
Kallimoides rumia         ............................................................  5162
Araschina levana          ............................................................  5081
Junonia stygia            ............................................................  5151
A. jatrophae saturata     ....................................................C.......  5136
Mallika jacksoni          ............................................................  5144
Helicoverpa zea           ............................................................  5261
Helicoverpa armigera      ............................................................  5239
Chrysodeixis includens    ............................................................  5062
Chrysodeixis eriosoma     ............................................................  5082
Chrysodeixis chalcites    ............................................................  5083
Coeliades ramanatek       ............................................................  5312
Meroptera pravella        ............................................................  5145
Salamis anteva            ............................................................  5157
Precis andremiaja         ............................................................  5148

Consensus                 GAGATGTCGCGCCGGTTCGTACCCATATCCGCAGCAGGTCTCCAAGGTGAAGAGCCTCTA  5199
Papilio xuthus            ............................................................  5257
Pro. anacardii duprei     ............................................................  5251
Kallimoides rumia         ............................................................  5222
Araschina levana          ............................................................  5141
Junonia stygia            ............................................................  5211
A. jatrophae saturata     ............................................................  5196
Mallika jacksoni          ............................................................  5204
Helicoverpa zea           ............................................................  5321
Helicoverpa armigera      ............................................................  5299
Chrysodeixis includens    ............................................................  5122
Chrysodeixis eriosoma     ............................................................  5142
Chrysodeixis chalcites    ............................................................  5143
Coeliades ramanatek       ............................................................  5372
Meroptera pravella        ............................................................  5205
Salamis anteva            ............................................................  5217
Precis andremiaja         ............................................................  5208

Consensus                 GTCGATAGAATAATGTAGGTAAGGGAAGTCGGCAAATTGGATCCGTAACTTCGGAATAAG  5259
Papilio xuthus            ............................................................  5317
Pro. anacardii duprei     ............................................................  5311
Kallimoides rumia         ............................................................  5282
Araschina levana          ............................................................  5201
Junonia stygia            ............................................................  5271
```

```
A. jatrophae saturata   ............................................................  5256
Mallika jacksoni        ............................................................  5264
Helicoverpa zea         ............................................................  5381
Helicoverpa armigera    ............................................................  5359
Chrysodeixis includens  ............................................................  5182
Chrysodeixis eriosoma   ............................................................  5202
Chrysodeixis chalcites  ............................................................  5203
Coeliades ramanatek     ............................................................  5432
Meroptera pravella      ............................................................  5265
Salamis anteva          ............................................................  5277
Precis andremiaja       ............................................................  5268

Consensus               GATTGGCTCTGAGGACCGGGGCGTGTCGGGTTTGGACGGGAAGCGGATGCGGCCGGTGCC  5319
Papilio xuthus          ............................................................  5377
Pro. anacardii duprei   ............................................................  5371
Kallimoides rumia       ............................................................  5342
Araschina levana        ............................................................  5261
Junonia stygia          ............................................................  5331
A. jatrophae saturata   ............................................................  5316
Mallika jacksoni        ............................................................  5324
Helicoverpa zea         ............................................................  5441
Helicoverpa armigera    ............................................................  5419
Chrysodeixis includens  ............................................................  5242
Chrysodeixis eriosoma   ............................................................  5262
Chrysodeixis chalcites  ............................................................  5263
Coeliades ramanatek     ............................................................  5492
Meroptera pravella      ............................................................  5325
Salamis anteva          ............................................................  5337
Precis andremiaja       ............................................................  5328

Consensus               GGGCCTGGTCGATGCTCGTGCGTCTCTN-GN-CG-TGC-CGT-----NN-NCTCTCGGGG  5369
Papilio xuthus          ......................CG...TTCTT.CT.CGCG.G-----TT--G.A....C.  5430
Pro. anacardii duprei   ...................T...G...CG.GG..A...G...-----TCGT.........  5426
Kallimoides rumia       ...................T......CCT.GT..A...T...-----TCGT.........  5397
Araschina levana        ............C......T.......CG.GG..TC..T...-----TCGT.........  5316
Junonia stygia          ...................T.......CG.GK..A...T...-----TCGT.........  5386
A. jatrophae saturata   ...................T.......CT.GT..A...T...-----TCGT.........  5371
Mallika jacksoni        ...................T.......CG.GT..A...T...-----TCGT.........  5379
Helicoverpa zea         .................TG.......----------------------------......  5473
Helicoverpa armigera    .................TG.......----------------------------......  5451
Chrysodeixis includens  .................TA......T----------------------------......  5274
Chrysodeixis eriosoma   .................TC......T----------------------------......  5294
Chrysodeixis chalcites  .................TC......T----------------------------......  5295
Coeliades ramanatek     ............A.....C.......----------------------------......  5524
Meroptera pravella      ...............CGTGC..GGC.CTG.TCGA-...CGTGCGTG--T-CTCTCG..AT  5381
Salamis anteva          ...................T....C..-----.CGS.RKSC.CGTTCGTCC...CG....  5392
Precis andremiaja       ..................C.TTCGKSYCTSGT..ANTGCTCGCGTTCGTCT...GG..C.  5388

Consensus               CGTGCGGGCGGAATCCGGACCCGCGTTCCGGCCTTCCGCGGATCTTCCTAGCCGTAAGGC  5429
Papilio xuthus          T...T....T.................................................T  5490
Pro. anacardii duprei   ..........................................................T.  5486
Kallimoides rumia       ............................................................  5457
Araschina levana        ...T..T.....................................................  5376
Junonia stygia          ............................................................  5446
A. jatrophae saturata   ............................................................  5431
Mallika jacksoni        ...T........................................................  5439
```

```
Helicoverpa zea        ..CTG.......................................................  5533
Helicoverpa armigera   ..CTG.......................................................  5511
Chrysodeixis includens ..C.A.......................................................  5334
Chrysodeixis eriosoma  ..C.A.......................................................  5354
Chrysodeixis chalcites ..C.A.......................................................  5355
Coeliades ramanatek    ......A.....................................................  5584
Meroptera pravella     GCGTGC.......G..............................................  5441
Salamis anteva         ............................................................  5452
Precis andremiaja      T.C.........................................................  5448

Consensus              CGYGTCGGTTTCGTCTCGTGCGCGATCGGCGCGGTTCTGTACGACCGCCGTTCAACGGTC  5489
Papilio xuthus         ..T...........T..................CGC........................  5550
Pro. anacardii duprei  ..C......................C..................................  5546
Kallimoides rumia      ..C.........................................................  5517
Araschina levana       ..C......................C..................................  5436
Junonia stygia         ..C.........................................................  5506
A. jatrophae saturata  ..C.........................................................  5491
Mallika jacksoni       ..C.........................................................  5499
Helicoverpa zea        ..T.........................................................  5593
Helicoverpa armigera   ..T.........................................................  5571
Chrysodeixis includens ..T...T..C...A..............................................  5394
Chrysodeixis eriosoma  ..T......C...A..............................................  5414
Chrysodeixis chalcites ..T......C...A..............................................  5415
Coeliades ramanatek    ..C.........................................................  5644
Meroptera pravella     ..T.........................................................  5501
Salamis anteva         ..T.........................................................  5512
Precis andremiaja      ..C.........................................................  5508

Consensus              AGCTCAGAACTGGCACGGACAAGGGGAATCCGACTGTCTAATTAAAACAAAGCATTGCGA  5549
Papilio xuthus         ............................................................  5610
Pro. anacardii duprei  ............................................................  5606
Kallimoides rumia      ............................................................  5577
Araschina levana       ............................................................  5496
Junonia stygia         ............................................................  5566
A. jatrophae saturata  ............................................................  5551
Mallika jacksoni       ............................................................  5559
Helicoverpa zea        ............................................................  5653
Helicoverpa armigera   ............................................................  5631
Chrysodeixis includens ............................................................  5454
Chrysodeixis eriosoma  ............................................................  5474
Chrysodeixis chalcites ............................................................  5475
Coeliades ramanatek    ............................................................  5704
Meroptera pravella     ............................................................  5561
Salamis anteva         ............................................................  5572
Precis andremiaja      ............................................................  5568

Consensus              TGGCCCTCGCGGGTGTTGACGCAATGTGATTTCTGCCCAGTGCTCTGAATGTCAACGTGA  5609
Papilio xuthus         .......A....................................................  5670
Pro. anacardii duprei  ............................................................  5666
Kallimoides rumia      ............................................................  5637
Araschina levana       ............................................................  5556
Junonia stygia         ............................................................  5626
A. jatrophae saturata  ............................................................  5611
Mallika jacksoni       ............................................................  5619
Helicoverpa zea        ............................................................  5713
Helicoverpa armigera   ............................................................  5691
```

```
Chrysodeixis includens ............................................................  5514
Chrysodeixis eriosoma  ............................................................  5534
Chrysodeixis chalcites ............................................................  5535
Coeliades ramanatek    ............................................................  5764
Meroptera pravella     ............................................................  5621
Salamis anteva         ............................................................  5632
Precis andremiaja      ............................................................  5628

Consensus              AGAAATTCAAGCAAGCGCGGGTAAACGGCGGGAGTAACTATGACTCTCTTAAGGTAGCCA  5669
Papilio xuthus         ............................................................  5730
Pro. anacardii duprei  ............................................................  5726
Kallimoides rumia      ............................................................  5697
Araschina levana       ............................................................  5616
Junonia stygia         ............................................................  5686
A. jatrophae saturata  ............................................................  5671
Mallika jacksoni       ............................................................  5679
Helicoverpa zea        ............................................................  5773
Helicoverpa armigera   ............................................................  5751
Chrysodeixis includens ............................................................  5574
Chrysodeixis eriosoma  ............................................................  5594
Chrysodeixis chalcites ............................................................  5595
Coeliades ramanatek    ............................................................  5824
Meroptera pravella     ............................................................  5681
Salamis anteva         ............................................................  5692
Precis andremiaja      ............................................................  5688

Consensus              AATGCCTCGTCATCTAATTAGTGACGCGCATGAATGGATTAACGAGATTCCCRCTGTCCC  5729
Papilio xuthus         ....................................................A.......  5790
Pro. anacardii duprei  ....................................................G.......  5786
Kallimoides rumia      ....................................................G.......  5757
Araschina levana       ....................................................G.......  5676
Junonia stygia         ....................................................G.......  5746
A. jatrophae saturata  ....................................................G.......  5731
Mallika jacksoni       ....................................................G.......  5739
Helicoverpa zea        ....................................................A.......  5833
Helicoverpa armigera   ....................................................A.......  5811
Chrysodeixis includens ....................................................A.......  5634
Chrysodeixis eriosoma  ....................................................A.......  5654
Chrysodeixis chalcites ....................................................A.......  5655
Coeliades ramanatek    ....................................................A.......  5884
Meroptera pravella     ....................................................A.......  5741
Salamis anteva         ....................................................G.......  5752
Precis andremiaja      ....................................................G.......  5748

Consensus              TATCTACTATCTAGCGAAACCACAGCCAAGGGAACGGGCTTGGGAGAATCAGCGGGGAAA  5789
Papilio xuthus         ............................................................  5850
Pro. anacardii duprei  ............................................................  5846
Kallimoides rumia      ............................................................  5817
Araschina levana       ............................................................  5736
Junonia stygia         ............................................................  5806
A. jatrophae saturata  ....C.......................................................  5791
Mallika jacksoni       ............................................................  5799
Helicoverpa zea        ............................................................  5893
Helicoverpa armigera   ............................................................  5871
Chrysodeixis includens ............................................................  5694
Chrysodeixis eriosoma  ............................................................  5714
```

```
Chrysodeixis chalcites   ............................................................  5715
Coeliades ramanatek      ............................................................  5944
Meroptera pravella       ............................................................  5801
Salamis anteva           ............................................................  5812
Precis andremiaja        ............................................................  5808

Consensus                GAAGACCCTGTTGAGCTTGACTCTAGTCTGGCATTGTAAGGAGACATGAGAGGTGTAGCA  5849
Papilio xuthus           ............................................................  5910
Pro. anacardii duprei    ............................................................  5906
Kallimoides rumia        ............................................................  5877
Araschina levana         ............................................................  5796
Junonia stygia           ............................................................  5866
A. jatrophae saturata    ............................................................  5851
Mallika jacksoni         ............................................................  5859
Helicoverpa zea          ............................................................  5953
Helicoverpa armigera     ............................................................  5931
Chrysodeixis includens   ............................................................  5754
Chrysodeixis eriosoma    ............................................................  5774
Chrysodeixis chalcites   ............................................................  5775
Coeliades ramanatek      ............................................................  6004
Meroptera pravella       ............................................................  5861
Salamis anteva           ............................................................  5872
Precis andremiaja        ............................................................  5868

Consensus                TAAGTGGGAGATCGTTCGC-CGGTCGTCGCTGAAAAACCACTACTTTCATTGTTTCATTA  5908
Papilio xuthus           ...................G........................................  5970
Pro. anacardii duprei    ..................--........................................  5964
Kallimoides rumia        ..................--........................................  5935
Araschina levana         ..................--........................................  5854
Junonia stygia           ..................--........................................  5924
A. jatrophae saturata    ..................--........................................  5909
Mallika jacksoni         ..................--........................................  5917
Helicoverpa zea          ...................G..A.....................................  6013
Helicoverpa armigera     ...................G..A.....................................  5991
Chrysodeixis includens   ...................G..A.....................................  5814
Chrysodeixis eriosoma    ...................G..A.....................................  5834
Chrysodeixis chalcites   ...................G..A.....................................  5835
Coeliades ramanatek      ...................--.......................................  6062
Meroptera pravella       ...................--.......................................  5919
Salamis anteva           ...................--.......................................  5930
Precis andremiaja        ...................--.......................................  5926

Consensus                CTTACTCGGTTGGGCGGAAGCGGTGCGCGGTCGATAA--TATCGGCGGGCGYACGGTGTT  5966
Papilio xuthus           ...................................TT--C...........C........  6028
Pro. anacardii duprei    ...................................C.--............T........  6022
Kallimoides rumia        ...................................T.--............T........  5993
Araschina levana         ...................................T.--............T........  5912
Junonia stygia           ...................................C.--............T........  5982
A. jatrophae saturata    .....................................--............T........  5967
Mallika jacksoni         ...................................T.--............T........  5975
Helicoverpa zea          .....................................-T-...........C........  6071
Helicoverpa armigera     .....................................-T-...........C........  6049
Chrysodeixis includens   ....................................T-CA...........C........  5873
Chrysodeixis eriosoma    ....................................T-TA...........C........  5893
Chrysodeixis chalcites   ....................................T-TA...........C........  5894
Coeliades ramanatek      ..................................CC.ACA...........C........  6122
```

```
Meroptera pravella     ..................G................T.--........A...C........  5977
Salamis anteva         ...................................C.--............T........  5988
Precis andremiaja      ...................................C.--............T........  5984

Consensus              TCGTTCCAAGCGTGCAGAGTGGCGGCGTGGCGGCAACGCTCGTCGCCTWACAACTCCCGC  6026
Papilio xuthus         ......................T................C.......GT...........  6088
Pro. anacardii duprei  ............................A.....G.............A...........  6082
Kallimoides rumia      ............................A.....G.....T.......A...........  6053
Araschina levana       ......................T.T...A....T..............A...........  5972
Junonia stygia         ............................A.....G.............A...........  6042
A. jatrophae saturata  ............................A....TG.............A...........  6027
Mallika jacksoni       ................................................A...........  6035
Helicoverpa zea        ........................A......................GT...........  6131
Helicoverpa armigera   ........................A......................GT...........  6109
Chrysodeixis includens ........................A......................GT.A.........  5933
Chrysodeixis eriosoma  ........................A......................GT.A.........  5953
Chrysodeixis chalcites ........................A......................GT.A.........  5954
Coeliades ramanatek    ........................T.......................T...........  6182
Meroptera pravella     ........................T.................G....GT...........  6037
Salamis anteva         ............................A.....G.............A...........  6048
Precis andremiaja      ............................A.....G.............A...........  6044

Consensus              GTGATCCGGTTCGAGGACACTGCCAGGCGGGGAGTTTGACTGGGCGGTACATCTGTCAA  6086
Papilio xuthus         ............................................................  6148
Pro. anacardii duprei  ............................................................  6142
Kallimoides rumia      ............................................................  6113
Araschina levana       ..........................................A.................  6032
Junonia stygia         ............................................................  6102
A. jatrophae saturata  ............................................................  6087
Mallika jacksoni       ............................................................  6095
Helicoverpa zea        ...............................T............................  6191
Helicoverpa armigera   ...............................T............................  6169
Chrysodeixis includens ...............................T............................  5993
Chrysodeixis eriosoma  ...............................T............................  6013
Chrysodeixis chalcites ...............................T............................  6014
Coeliades ramanatek    ............................................................  6242
Meroptera pravella     ............................................................  6097
Salamis anteva         ............................................................  6108
Precis andremiaja      ............................................................  6104

Consensus              AGAATAACGCAGGTGTCCTAAGGCCAGCTCAGCGAGGACAGAAACCTCGCGTGGAGCAAA  6146
Papilio xuthus         ............................................................  6208
Pro. anacardii duprei  ............................................................  6202
Kallimoides rumia      ............................................................  6173
Araschina levana       ............................................................  6092
Junonia stygia         ............................................................  6162
A. jatrophae saturata  ............................................................  6147
Mallika jacksoni       ............................................................  6155
Helicoverpa zea        ............................................................  6251
Helicoverpa armigera   ............................................................  6229
Chrysodeixis includens ............................................................  6053
Chrysodeixis eriosoma  ............................................................  6073
Chrysodeixis chalcites ............................................................  6074
Coeliades ramanatek    ............................................................  6302
Meroptera pravella     ....................................................A.......  6157
Salamis anteva         ............................................................  6168
```

```
Precis andremiaja          ............................................................  6164

Consensus                  AGGGCAAAAGCTGGCTTGATCCAGATGTTCAGTACGCATAGGGACTGCGAAAGCACGGCC  6206
Papilio xuthus             ............................................................  6268
Pro. anacardii duprei      ............................................................  6262
Kallimoides rumia          ............................................................  6233
Araschina levana           ............................................................  6152
Junonia stygia             ............................................................  6222
A. jatrophae saturata      ............................................................  6207
Mallika jacksoni           ............................................................  6215
Helicoverpa zea            ............................................................  6311
Helicoverpa armigera       ............................................................  6289
Chrysodeixis includens     ............................................................  6113
Chrysodeixis eriosoma      ............................................................  6133
Chrysodeixis chalcites     ............................................................  6134
Coeliades ramanatek        ............................................................  6362
Meroptera pravella         ............................................................  6217
Salamis anteva             ............................................................  6228
Precis andremiaja          ............................................................  6224

Consensus                  TATCGATCCTTTAGTATAAAGAGTTTTTAGCAAGAGGTGCCAGAAAAGTTACCACAGGGA  6266
Papilio xuthus             ............................................................  6328
Pro. anacardii duprei      ............................................................  6322
Kallimoides rumia          ............................................................  6293
Araschina levana           ............................................................  6212
Junonia stygia             ............................................................  6282
A. jatrophae saturata      ............................................................  6267
Mallika jacksoni           ............................................................  6275
Helicoverpa zea            ............................................................  6371
Helicoverpa armigera       ............................................................  6349
Chrysodeixis includens     ............................................................  6173
Chrysodeixis eriosoma      ............................................................  6193
Chrysodeixis chalcites     ............................................................  6194
Coeliades ramanatek        ............................................................  6422
Meroptera pravella         ............................................................  6277
Salamis anteva             ............................................................  6288
Precis andremiaja          ............................................................  6284

Consensus                  TAACTGGCTTGTGGCGGCCAAGCGTTCATAGCGACGTTGCTTTTTGATCCTTCGATGTCG  6326
Papilio xuthus             ............................................................  6388
Pro. anacardii duprei      ............................................................  6382
Kallimoides rumia          ........................................................R...  6353
Araschina levana           ......A.....................................................  6272
Junonia stygia             ............................................................  6342
A. jatrophae saturata      ............................................................  6327
Mallika jacksoni           ............................................................  6335
Helicoverpa zea            ...............A............................................  6431
Helicoverpa armigera       ...............A............................................  6409
Chrysodeixis includens     ...............A............................................  6233
Chrysodeixis eriosoma      ...............A............................................  6253
Chrysodeixis chalcites     ...............A............................................  6254
Coeliades ramanatek        ............................................................  6482
Meroptera pravella         ...............A............................................  6337
Salamis anteva             ............................................................  6348
Precis andremiaja          ............................................................  6344
```

```
Consensus                GCTCTTCCTATCATTGCGAAGCAAAATTCGCCAAGCGTTGGATTGTTCACCCATCAAAAG  6386
Papilio xuthus           ............................................................  6448
Pro. anacardii duprei    ............................................................  6442
Kallimoides rumia        ............................................................  6413
Araschina levana         ............................................................  6332
Junonia stygia           ............................................................  6402
A. jatrophae saturata    ............................................................  6387
Mallika jacksoni         ............................................................  6395
Helicoverpa zea          ............................................................  6491
Helicoverpa armigera     ............................................................  6469
Chrysodeixis includens   ............................................................  6293
Chrysodeixis eriosoma    ............................................................  6313
Chrysodeixis chalcites   ............................................................  6314
Coeliades ramanatek      ............................................................  6542
Meroptera pravella       ............................................................  6397
Salamis anteva           ............................................................  6408
Precis andremiaja        ............................................................  6404

Consensus                GGAACGTGAGCTGGGTTTAGACCGTCGTGAGACAGGTTAGTTTTACCCTACTGATGGCT-  6445
Papilio xuthus           ...........................................................-  6507
Pro. anacardii duprei    ...........................................................-  6501
Kallimoides rumia        ...........................................................-  6472
Araschina levana         ...........................................................-  6391
Junonia stygia           ...........................................................-  6461
A. jatrophae saturata    ...........................................................A  6447
Mallika jacksoni         ...........................................................-  6454
Helicoverpa zea          ...........................................................-  6550
Helicoverpa armigera     ...........................................................-  6528
Chrysodeixis includens   ...........................................................-  6352
Chrysodeixis eriosoma    ...........................................................-  6372
Chrysodeixis chalcites   ...........................................................-  6373
Coeliades ramanatek      ...........................................................-  6601
Meroptera pravella       ...........................................................-  6456
Salamis anteva           ...........................................................-  6467
Precis andremiaja        ...........................................................-  6463

Consensus                CGTCGTTGCGATAGTAATACTGCTCAGTACGAGAGGAACCGCAGTTTCGGACATTTGGTT  6505
Papilio xuthus           ............................................................  6567
Pro. anacardii duprei    ............................................................  6561
Kallimoides rumia        ............................................................  6532
Araschina levana         ............................................................  6451
Junonia stygia           ............................................................  6521
A. jatrophae saturata    ............................................................  6507
Mallika jacksoni         ............................................................  6514
Helicoverpa zea          T...........................................................  6610
Helicoverpa armigera     T...........................................................  6588
Chrysodeixis includens   T...........................................................  6412
Chrysodeixis eriosoma    T...........................................................  6432
Chrysodeixis chalcites   T...........................................................  6433
Coeliades ramanatek      ............................................................  6661
Meroptera pravella       T...........................................................  6516
Salamis anteva           ............................................................  6527
Precis andremiaja        ............................................................  6523

Consensus                CATGCACTCGGCCGAGCGGCCGGTGGTGCGAAGCTACCATCCGCGGGATTATGCCTGAAC  6565
Papilio xuthus           ............................................................  6627
```

```
Pro. anacardii duprei     ............................................................  6621
Kallimoides rumia         ............................................................  6592
Araschina levana          ............................................................  6511
Junonia stygia            ............................................................  6581
A. jatrophae saturata     ............................................................  6567
Mallika jacksoni          ............................................................  6574
Helicoverpa zea           ............................................................  6670
Helicoverpa armigera      ............................................................  6648
Chrysodeixis includens    ............................................................  6472
Chrysodeixis eriosoma     ............................................................  6492
Chrysodeixis chalcites    ............................................................  6493
Coeliades ramanatek       ............................................................  6721
Meroptera pravella        ............................................................  6576
Salamis anteva            ............................................................  6587
Precis andremiaja         ............................................................  6583

Consensus                 GCCTCTAAGGCCGAAGCCAGCCTAGCCGAATCCGGCAAGGATATGCTCACTGTGGAGCCC  6625
Papilio xuthus            ............................................T.............T.  6687
Pro. anacardii duprei     ............................................................  6681
Kallimoides rumia         ...............................................A............  6652
Araschina levana          ............................................................  6571
Junonia stygia            ............................................................  6641
A. jatrophae saturata     .................................................A..........  6627
Mallika jacksoni          ............................................................  6634
Helicoverpa zea           ............................................................  6730
Helicoverpa armigera      ............................................................  6708
Chrysodeixis includens    .-----------------------------------------------------------  6532
Chrysodeixis eriosoma     .-----------------------------------------------------------  6552
Chrysodeixis chalcites    .-----------------------------------------------------------  6553
Coeliades ramanatek       ............................................................  6781
Meroptera pravella        ............................................................  6636
Salamis anteva            ............................................................  6647
Precis andremiaja         ............................................................  6643

Consensus                 CGAGAGTCGGGAGGCTCTAAACAATGTGACTTTACTAGTCGCGCTTTATTC---GTAAGG  6682
Papilio xuthus            ............................T................GC.C..---..GC..  6744
Pro. anacardii duprei     ...................................................---......  6738
Kallimoides rumia         ...................................................---......  6709
Araschina levana          ......................G............................---......  6628
Junonia stygia            ...................................................---......  6698
A. jatrophae saturata     ...................................................---......  6684
Mallika jacksoni          ...................................................---......  6691
Helicoverpa zea           ..............................................C.CCA--C..G...  6788
Helicoverpa armigera      ..............................................C.CCA--C..G...  6766
Chrysodeixis includens    ------------------------------------------------------------  6592
Chrysodeixis eriosoma     ------------------------------------------------------------  6612
Chrysodeixis chalcites    ------------------------------------------------------------  6613
Coeliades ramanatek       .............................................C.....---...GA.  6838
Meroptera pravella        ..............................................C.C.TCGC..G...  6696
Salamis anteva            ...................................................---......  6704
Precis andremiaja         ...................................................---......  6700

Consensus                 TGCGACGTCGAAGCCCATTTGGAACGCGGCGATCGATGCGAGCGGTCTTAACACG--TGC  6740
Papilio xuthus            ..........G.........A.T.CG..T.....G...T.T....T........TA...  6804
Pro. anacardii duprei     .......................................................--...  6796
Kallimoides rumia         ...........G...........................................--...  6767
```

```
Araschina levana       .......................................................--...  6686
Junonia stygia         .......................................................--...  6756
A. jatrophae saturata  .......................................................--...  6742
Mallika jacksoni       .......................................................--...  6749
Helicoverpa zea        .......................T.....A.........TGT.C-G......G..--...  6845
Helicoverpa armigera   .......................T.....A.........TGT.C-G......G..--...  6823
Chrysodeixis includens ------------------------------------------------------------  6652
Chrysodeixis eriosoma  ------------------------------------------------------------  6672
Chrysodeixis chalcites ------------------------------------------------------------  6673
Coeliades ramanatek    .......................T.....T..........GT....T.....G..--...  6896
Meroptera pravella     .......................T.....A.....G....GT..-GT........--...  6753
Salamis anteva         .......................................................--...  6762
Precis andremiaja      .......................................................--...  6758

Consensus              ATCACGGCGCCGAAGTTTCGAATATACCTCAGTTCGATGTCGGGGCTCGGAATAGTCTGT  6800
Papilio xuthus         ...........C.G.G.......TA.G................A................  6864
Pro. anacardii duprei  ............................................................  6856
Kallimoides rumia      ......................A.....................................  6827
Araschina levana       ....T.......................................................  6746
Junonia stygia         ............................................................  6816
A. jatrophae saturata  ............................................................  6802
Mallika jacksoni       ............................................................  6809
Helicoverpa zea        ........T......G.C.....T....................................  6905
Helicoverpa armigera   ........T......G.C.....T....................................  6883
Chrysodeixis includens ------------------------------------------------------------  6712
Chrysodeixis eriosoma  ------------------------------------------------------------  6732
Chrysodeixis chalcites ------------------------------------------------------------  6733
Coeliades ramanatek    ........A......G.C.....T....................................  6956
Meroptera pravella     GC......T......G.C.....C....................................  6813
Salamis anteva         ............................................................  6822
Precis andremiaja      ............................................................  6818

Consensus              AGACGACTTACGTTCCTGGCGGGGTGTTGTGCTCGGTAGAGCAGCGTCGTGCTGCGATCT  6860
Papilio xuthus         ............................................................  6924
Pro. anacardii duprei  ............................................................  6916
Kallimoides rumia      ............................................................  6887
Araschina levana       ............................................................  6806
Junonia stygia         ............................................................  6876
A. jatrophae saturata  ............................................................  6862
Mallika jacksoni       ............................................................  6869
Helicoverpa zea        .........C..................................................  6965
Helicoverpa armigera   .........C..................................................  6943
Chrysodeixis includens ------------------------------------------------------------  6772
Chrysodeixis eriosoma  ------------------------------------------------------------  6792
Chrysodeixis chalcites ------------------------------------------------------------  6793
Coeliades ramanatek    ............................................................  7016
Meroptera pravella     .........C..................................................  6873
Salamis anteva         ............................................................  6882
Precis andremiaja      ............................................................  6878

Consensus              GTTGAGACTCAGCCCTACGCCAGGTGATTCGT                              6892
Papilio xuthus         ................................                              6956
Pro. anacardii duprei  ................................                              6948
Kallimoides rumia      ..........T.....................                              6919
Araschina levana       ................................                              6838
Junonia stygia         ................................                              6908
```

```
A. jatrophae saturata  ................................                        6894
Mallika jacksoni       ................................                        6901
Helicoverpa zea        ................................                        6997
Helicoverpa armigera   ................................                        6975
Chrysodeixis includens --------------------------------                        6473
Chrysodeixis eriosoma  --------------------------------                        6493
Chrysodeixis chalcites --------------------------------                        6494
Coeliades ramanatek    ................................                        7048
Meroptera pravella     ................................                        6905
Salamis anteva         ..........G.....................                        6914
Precis andremiaja      ................................                        6910
```

45S rDNA Forward Sequencing Primer

45S rDNA Reverse Sequencing Primer
